# Supplementary material for: Combination of Everolimus and Bortezomib Inhibits the Growth and Metastasis of Bone and Soft Tissue Sarcomas via JNK/p38/ERK MAPK and AKT Pathways
Source: Cancers (Basel). 2023 Apr 26;15(9):2468. doi: 10.3390/cancers15092468 (PMC10177427; doi:10.3390/cancers15092468)
Supplement: Supplementary file 1 [file cancers-15-02468-s001.zip › cancers-2249367-supplementary.pdf]

Western Blot data

HT1080 treatment 6 h, 12 h

Treatment time 6 h

- 1. Everolimus 0 μM
- 2. Everolimus 5 μM
- 3. Everolimus 10 μM
- 4. Everolimus 20 μM
- 5. Everolimus 0 μM Bortezomib 5nM
- 6. Everolimus 5 μM Bortezomib 5nM
- 7. Everolimus 10 μM Bortezomib 5nM
- 8. Everolimus 20 μM Bortezomib 5nM

Treatment time 12 h

- 9. Everolimus 0 μM
- 10. Everolimus 5 μM
- 11. Everolimus 10 μM
- 12. Everolimus 20 μM
- 13. Everolimus 0 μM Bortezomib 5nM
- 14. Everolimus 5 μM Bortezomib 5nM
- 15. Everolimus 10 μM Bortezomib 5nM
- 16. Everolimus 20 μM Bortezomib 5nM

HT1080 6 h 12 h treatment β actin

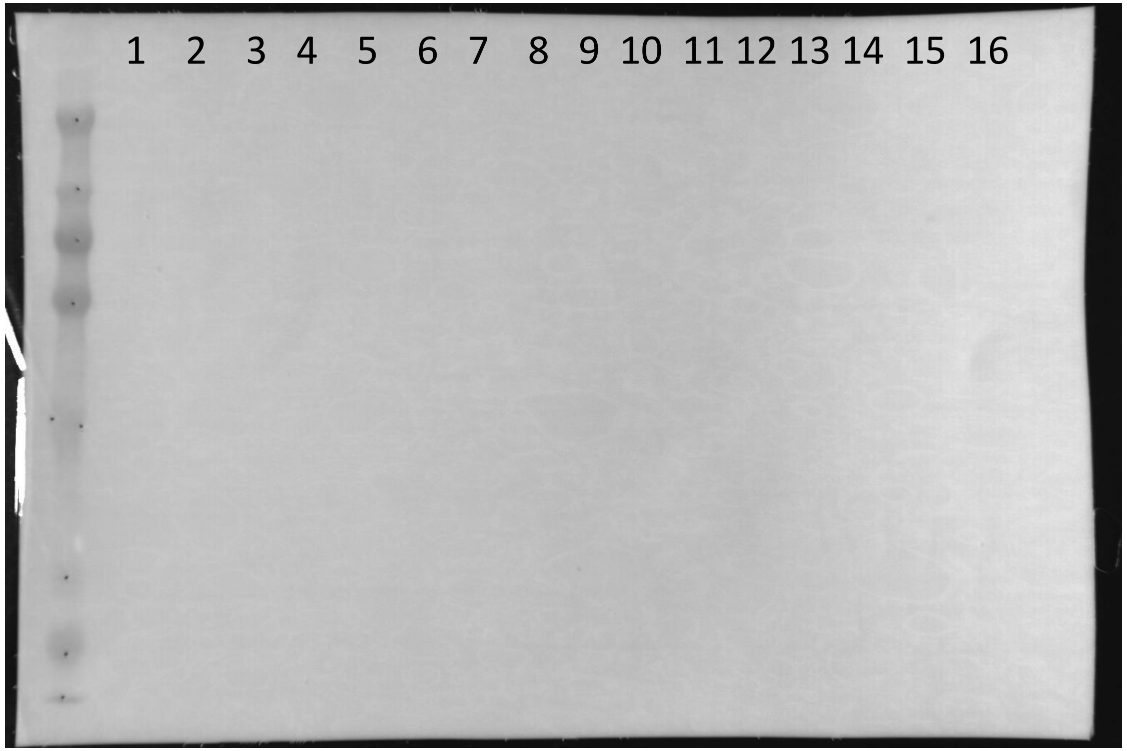

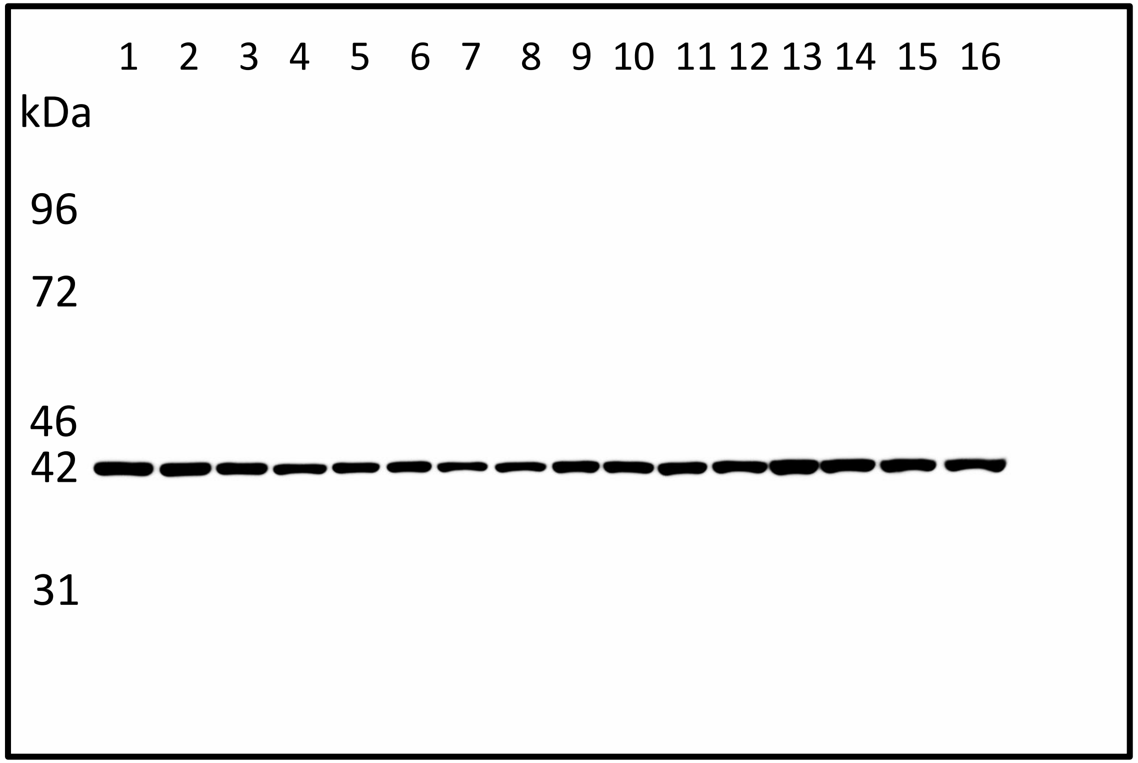

100:77:78:64:54:42:50:59:60:69:71:67:74:79:69:62

HT1080 6 h 12 h treatment BIK

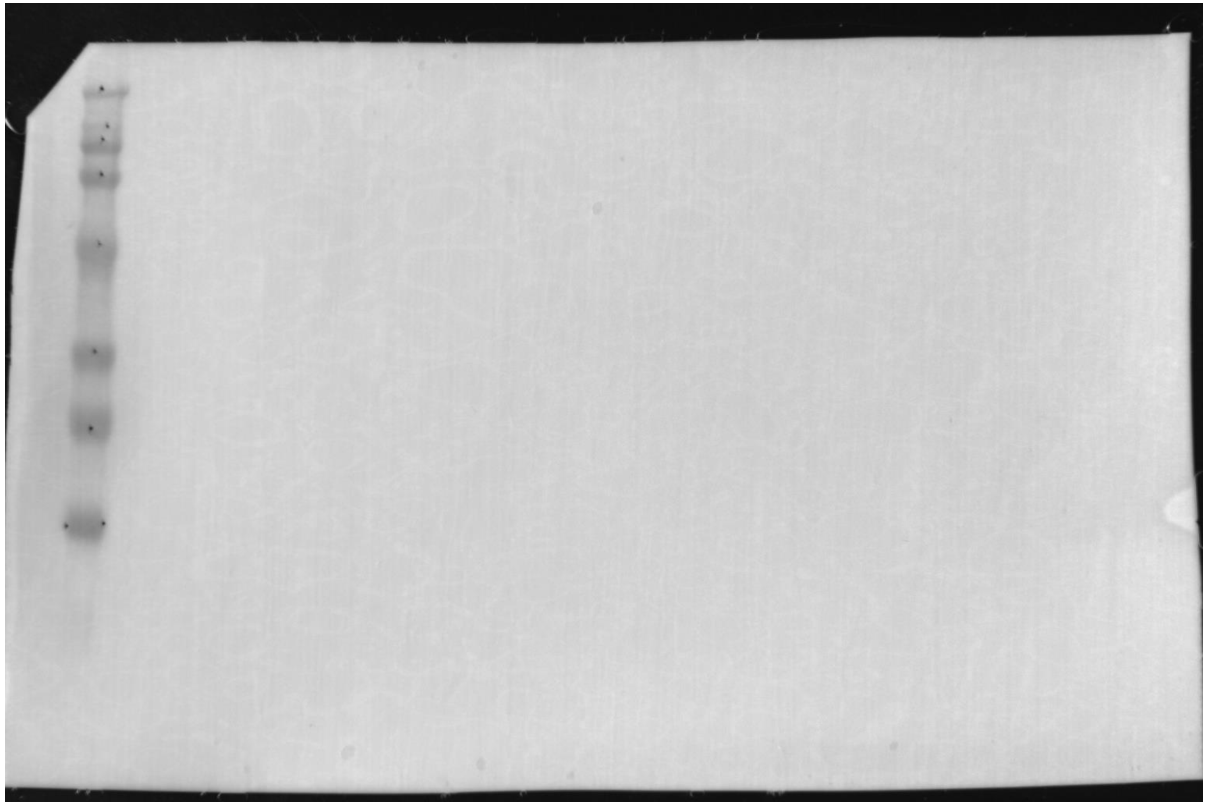

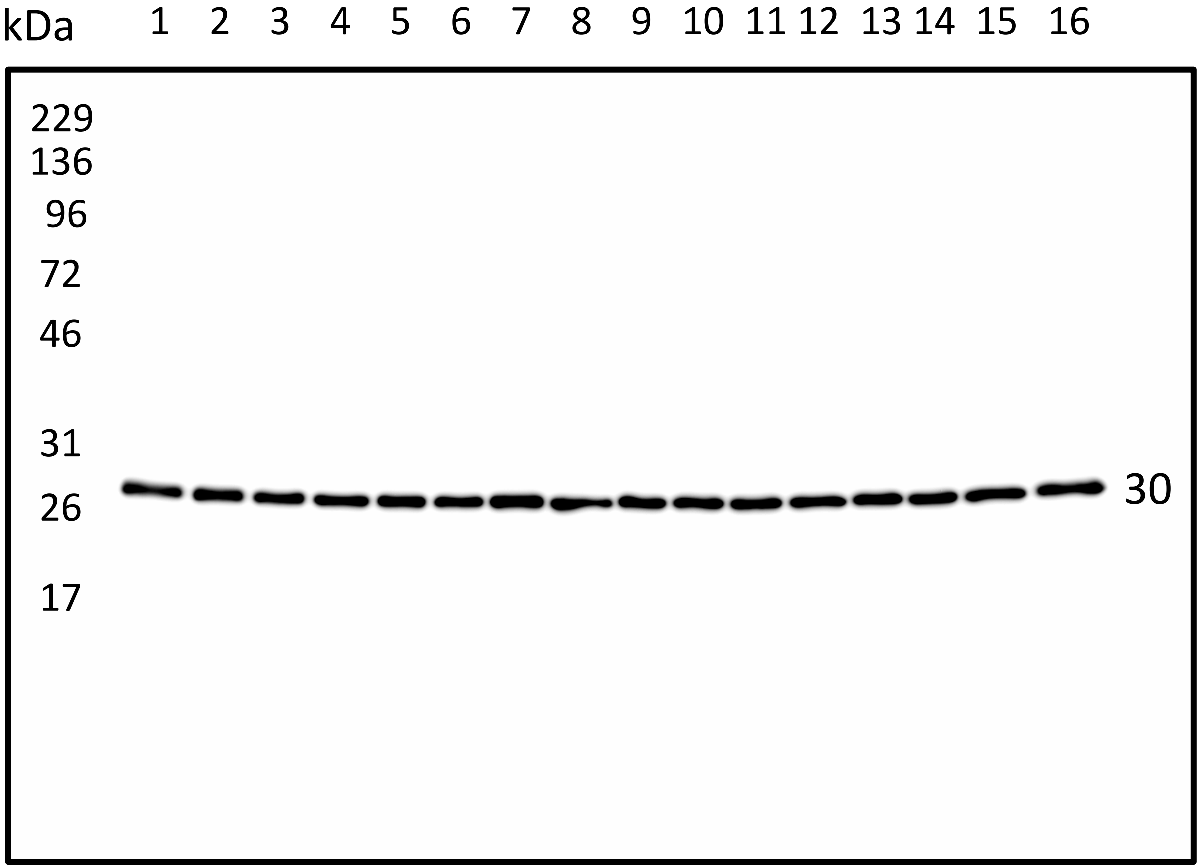

100:99:94:136:90:82:150:112:76:88:86:119:99:90:124:158

HT1080 6 h 12 h treatment cleaved caspase 9

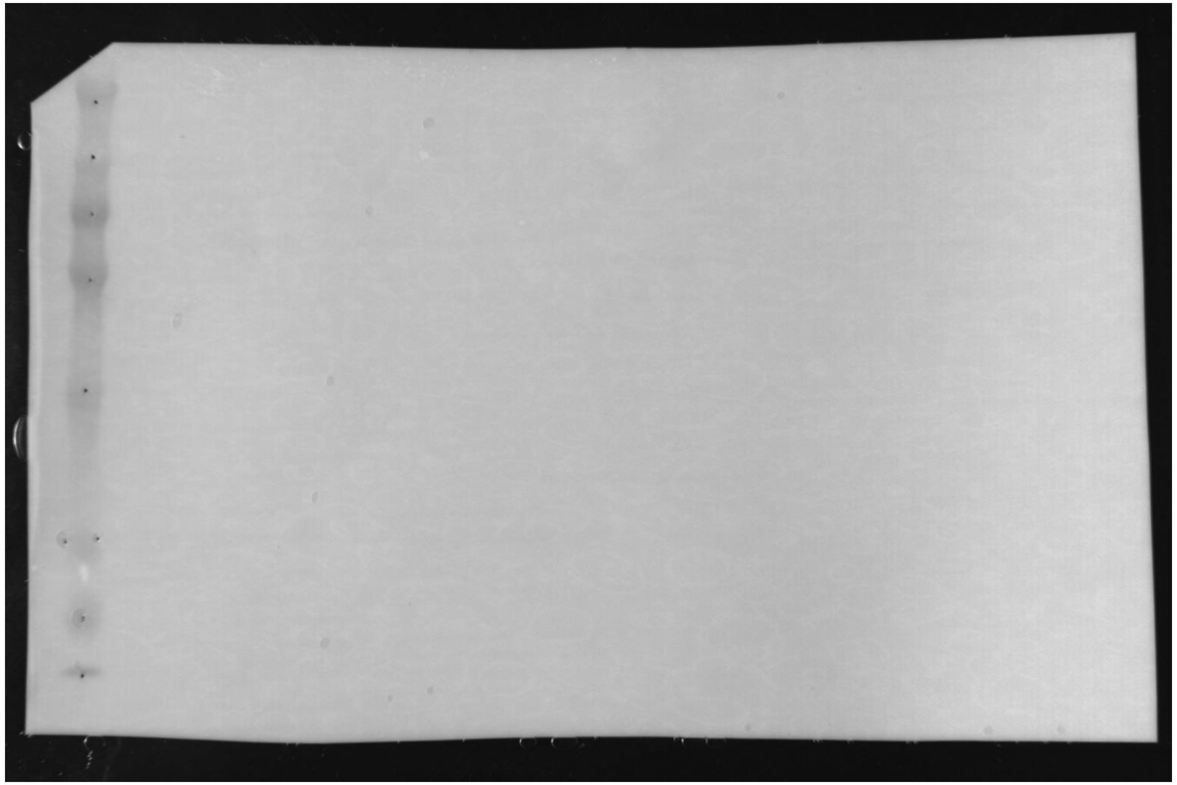

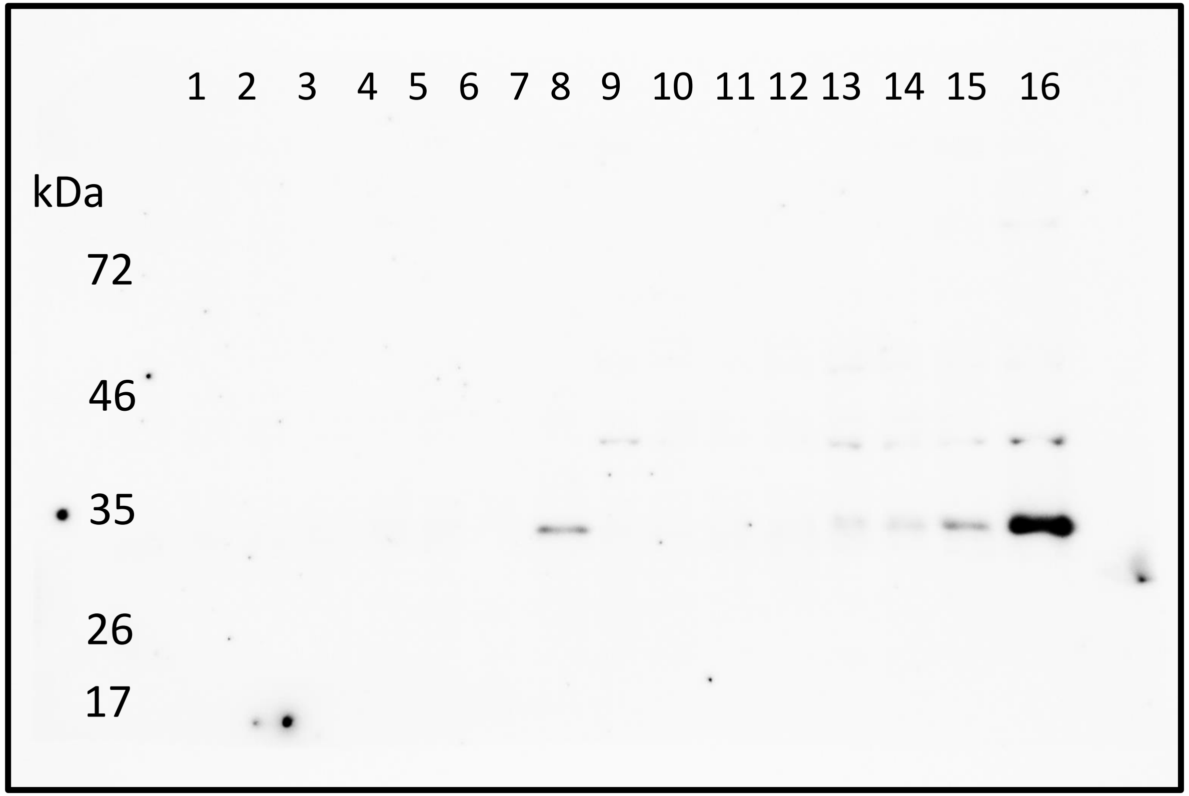

0:0:0:0:0:0:0:4:0:0:0:0:0:1:4:100

HT1080 6 h 12 h treatment cleaved caspase 8

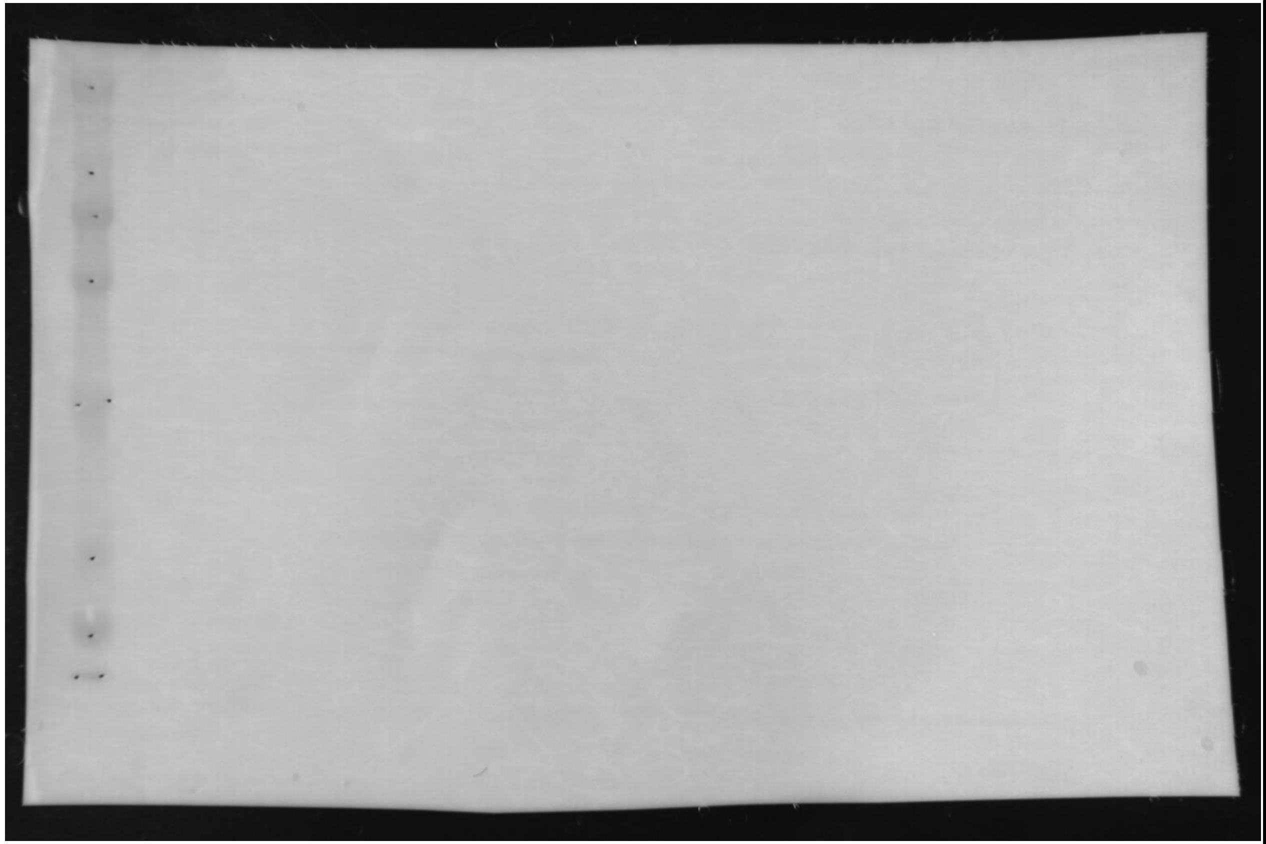

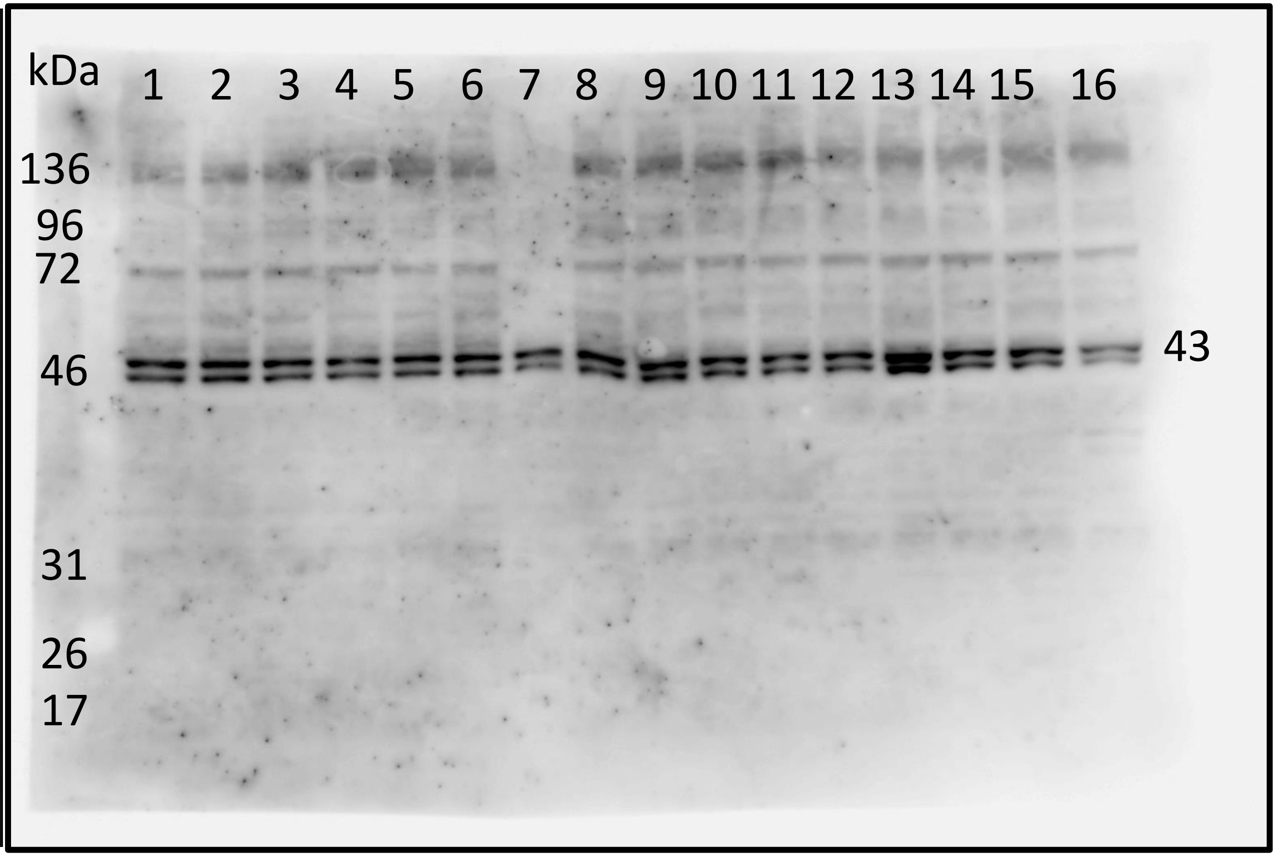

43kDa: 100:103:83:72:69:75:50:77:99:76:55:65:125:78:66:26

HT1080 6 h 12 h treatment cleaved caspase 3

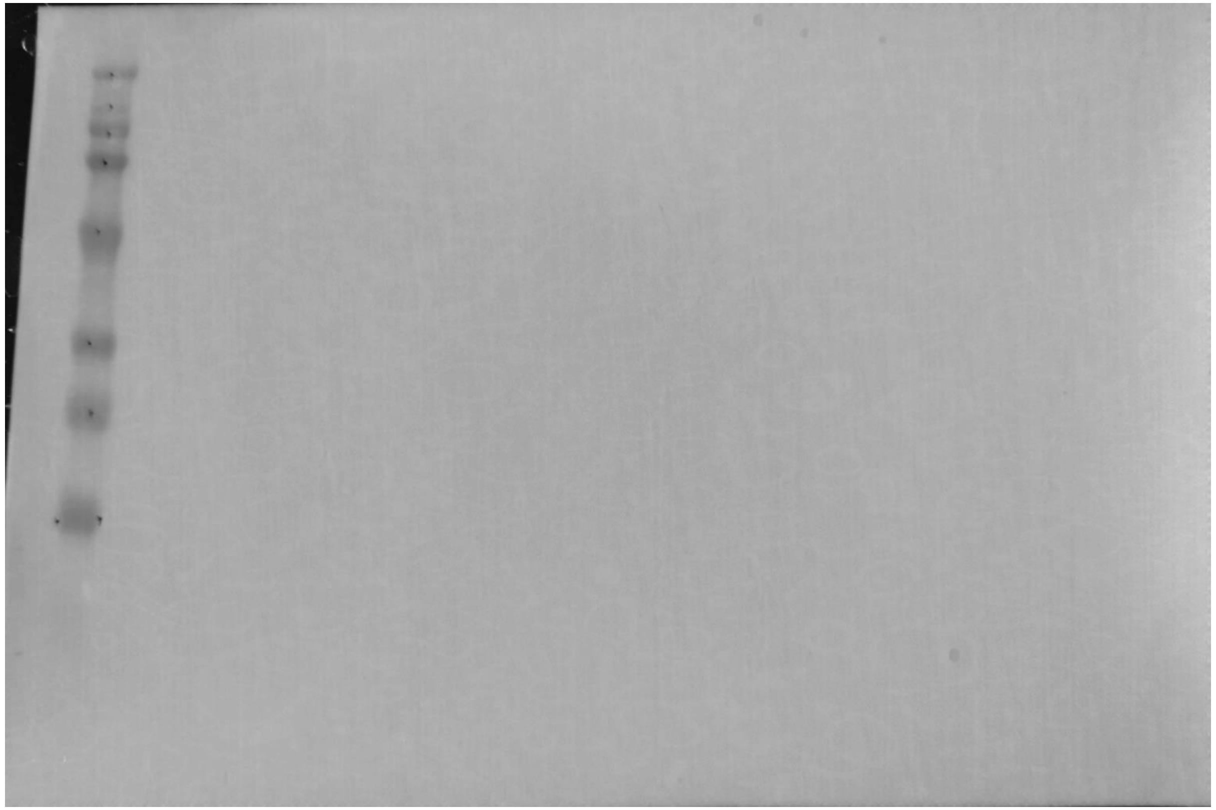

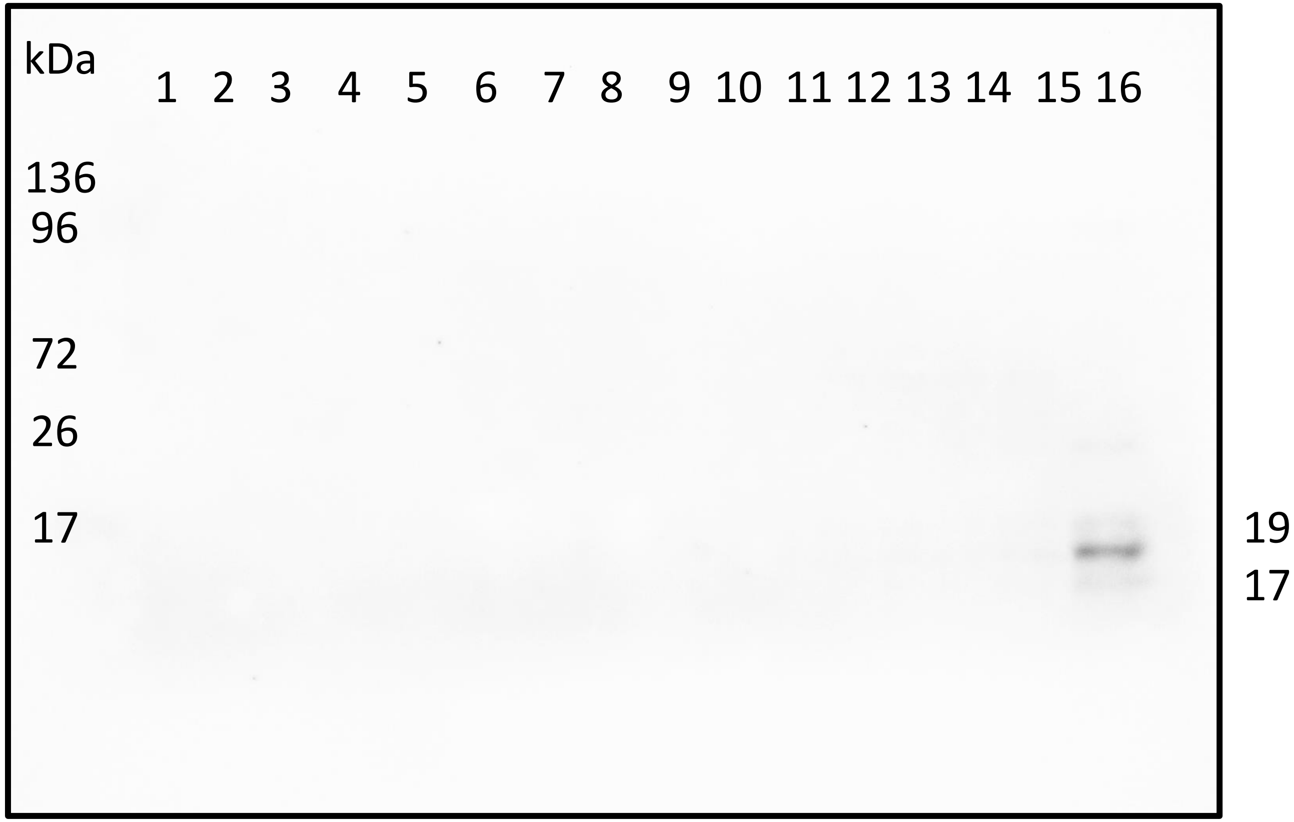

0:0:0:0:0:0:0:0:0:0:0:0:0:6:7:100

HT1080 6 h 12 h treatment cleaved PARP

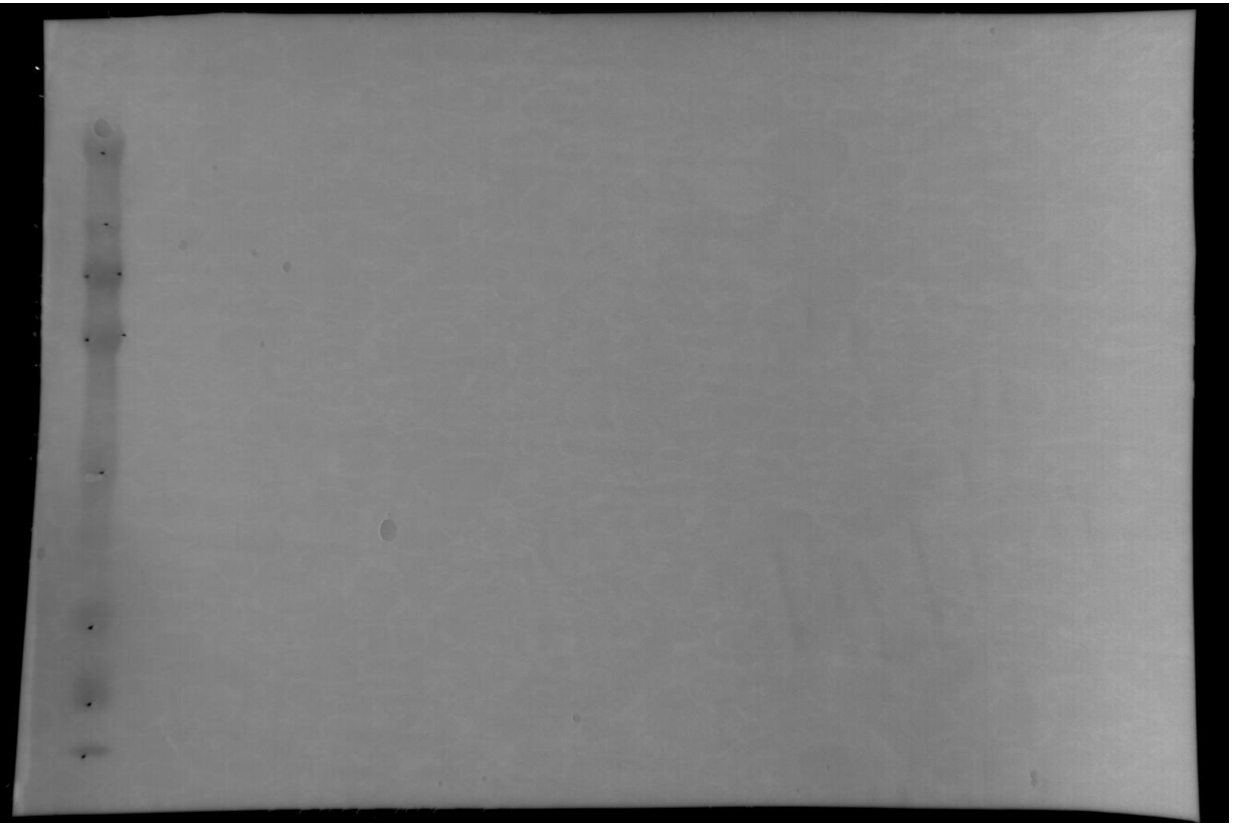

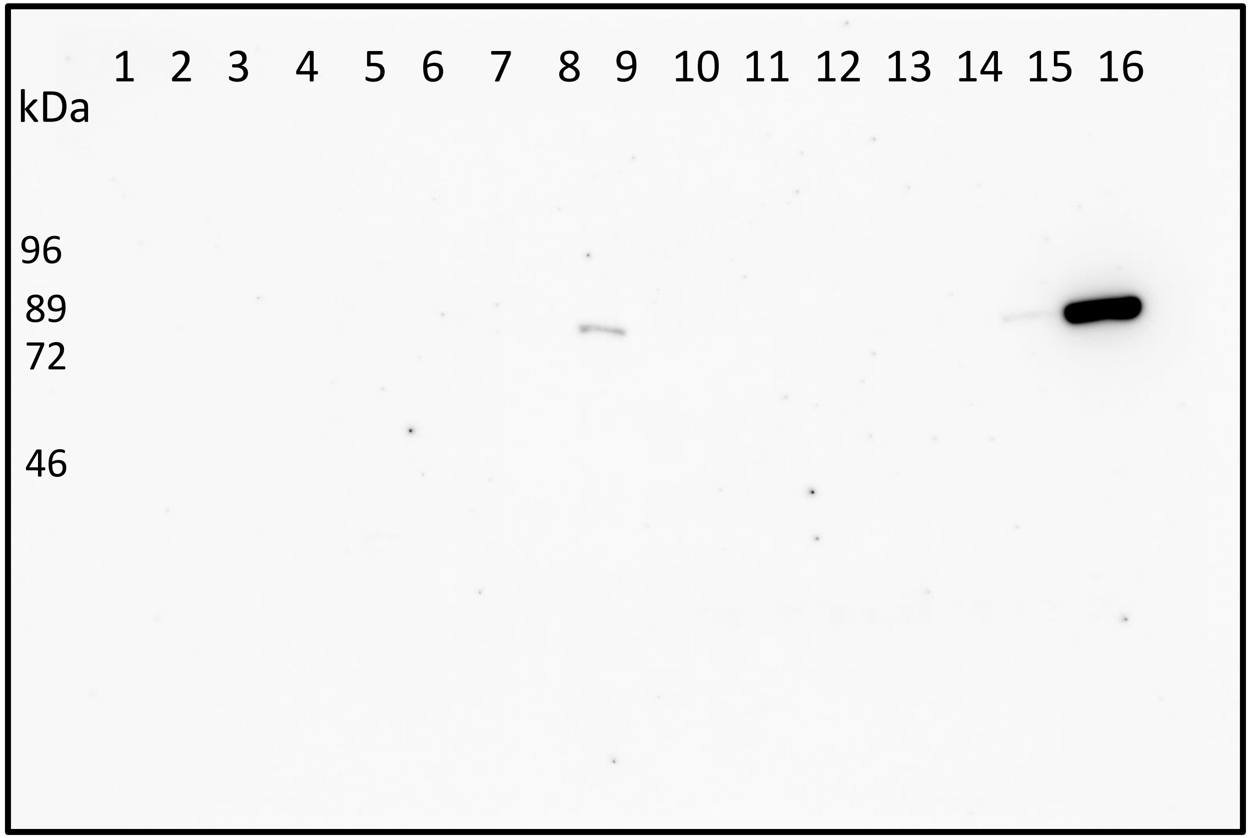

0:0:0:0:0:0:0:1:0:0:0:0:0:0:1:100

HT1080 6 h 12 h treatment p-ERK

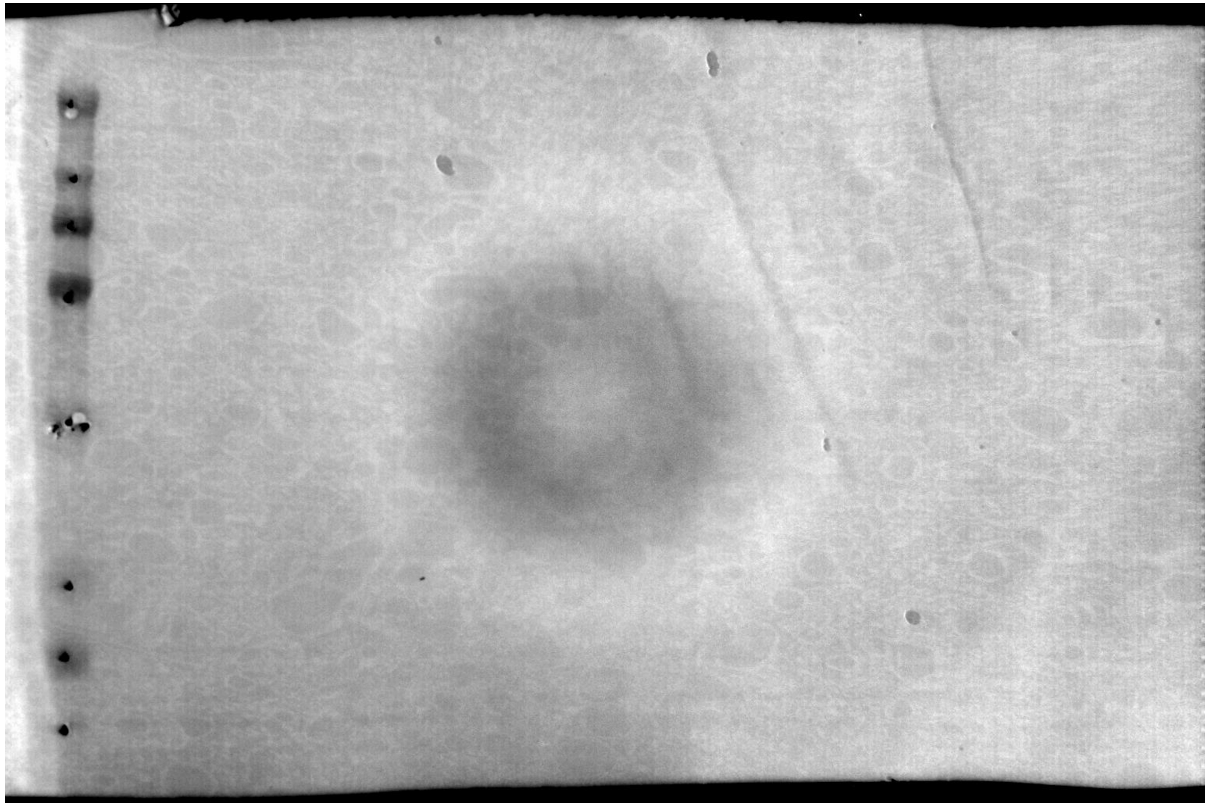

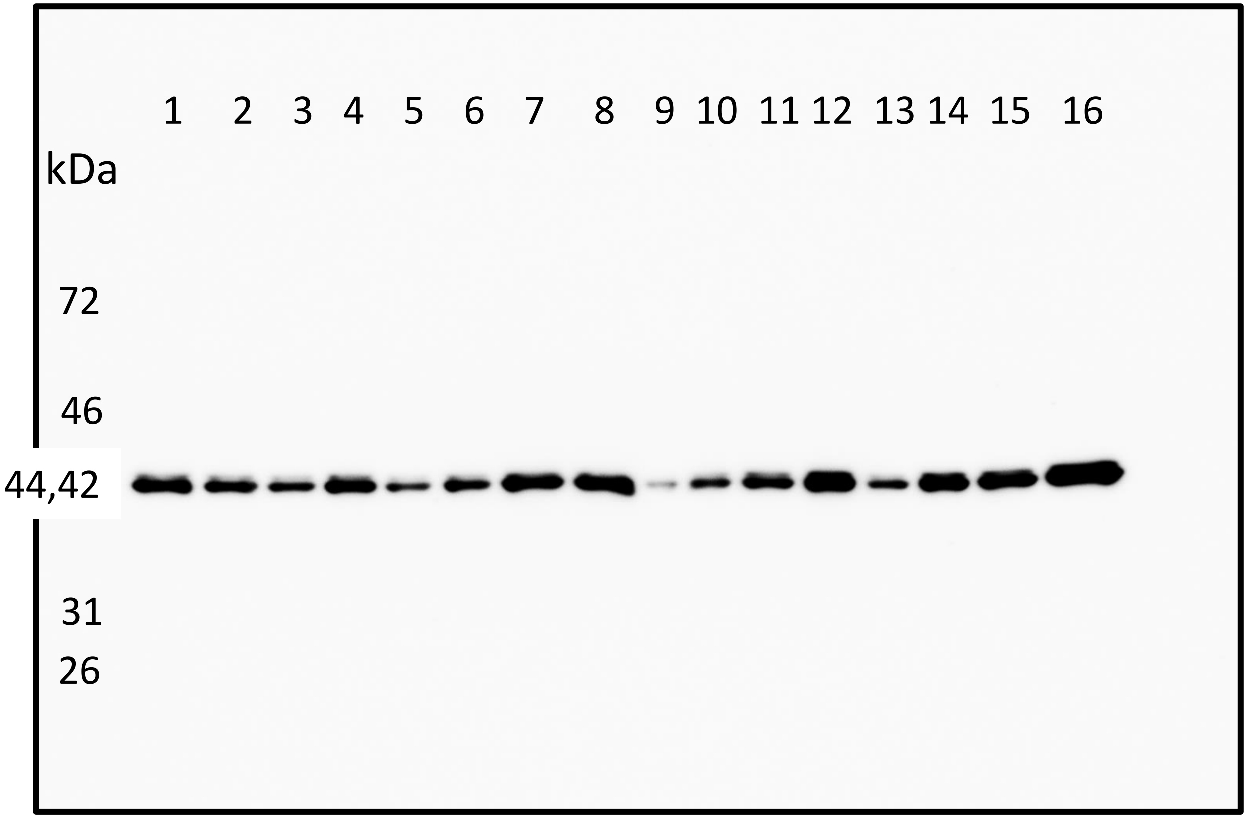

100:51:30:65:17:36:78:111:3:21:49:114:21:71:111:181

HT1080 6 h 12 h treatment ERK

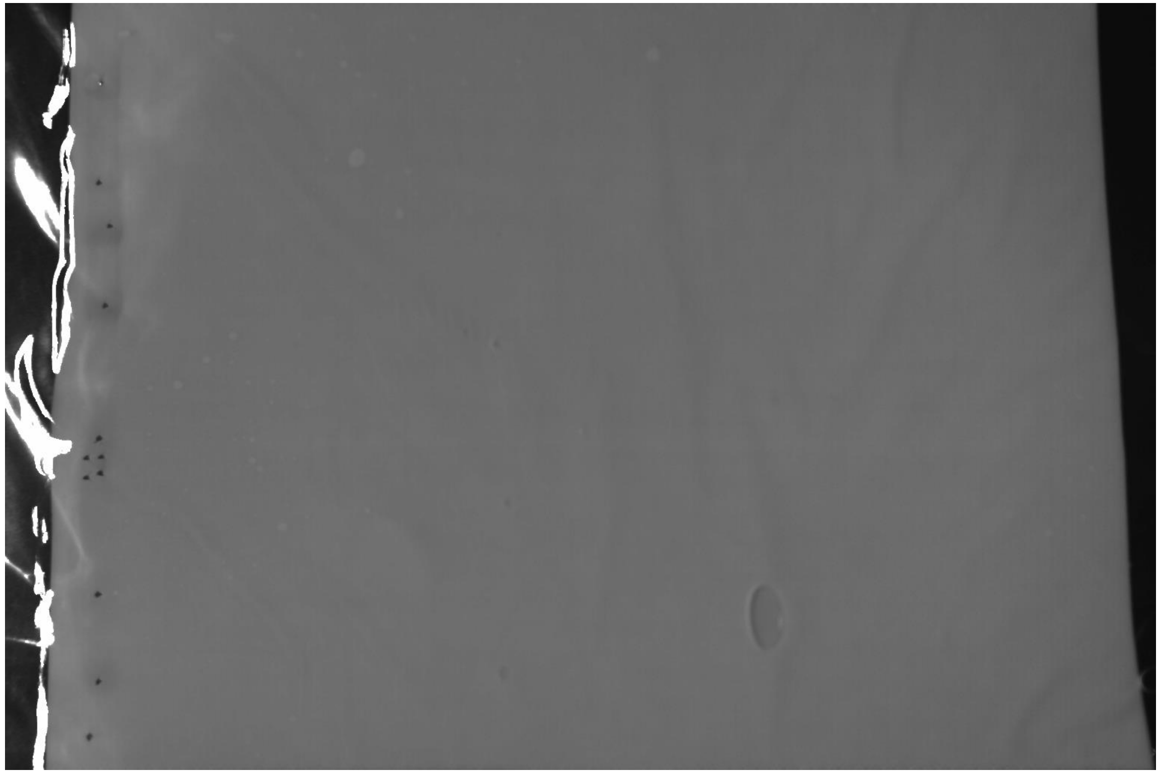

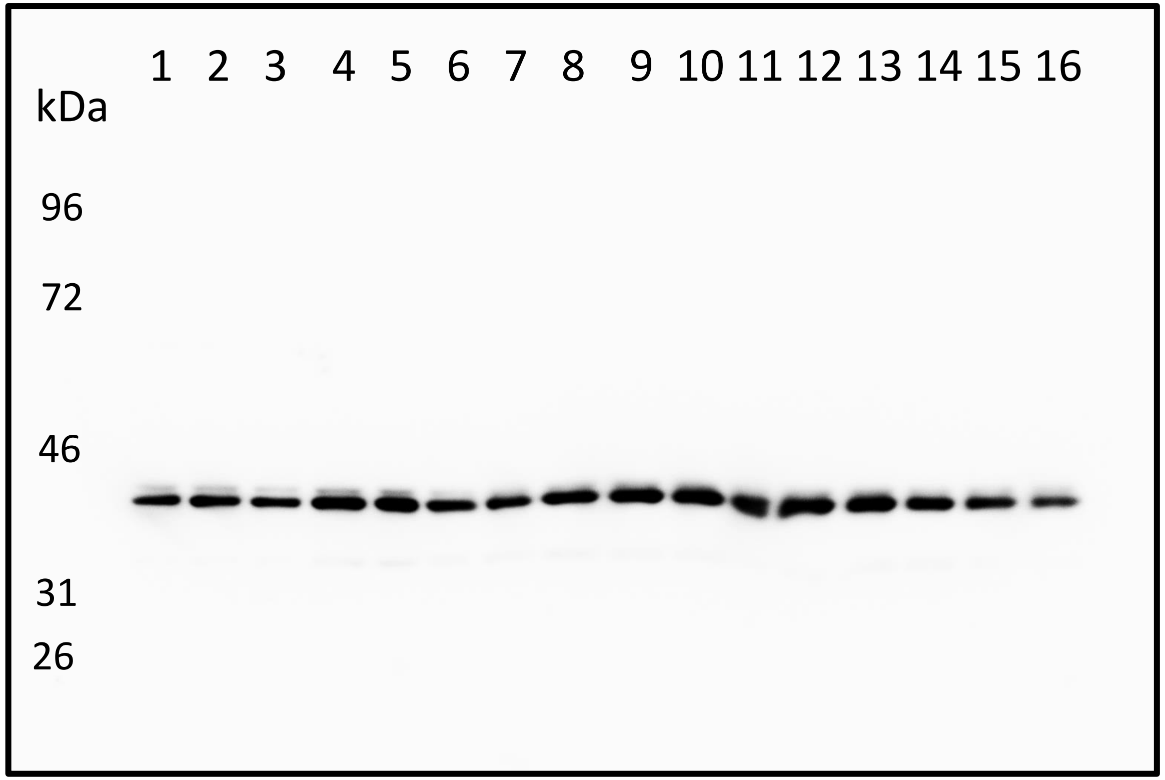

100:159:104:256:137:117:94:204:248:214:90:229:202:146:110:63

HT1080 6 h 12 h treatment p-JNK

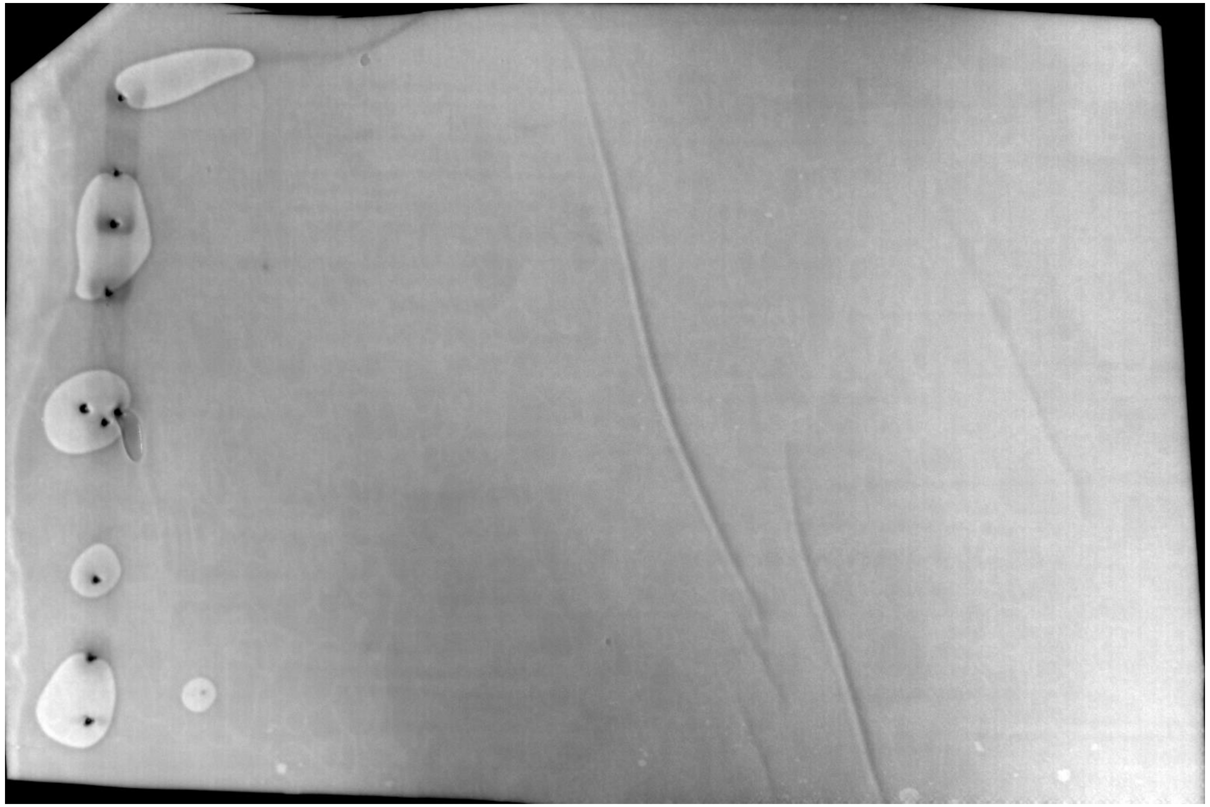

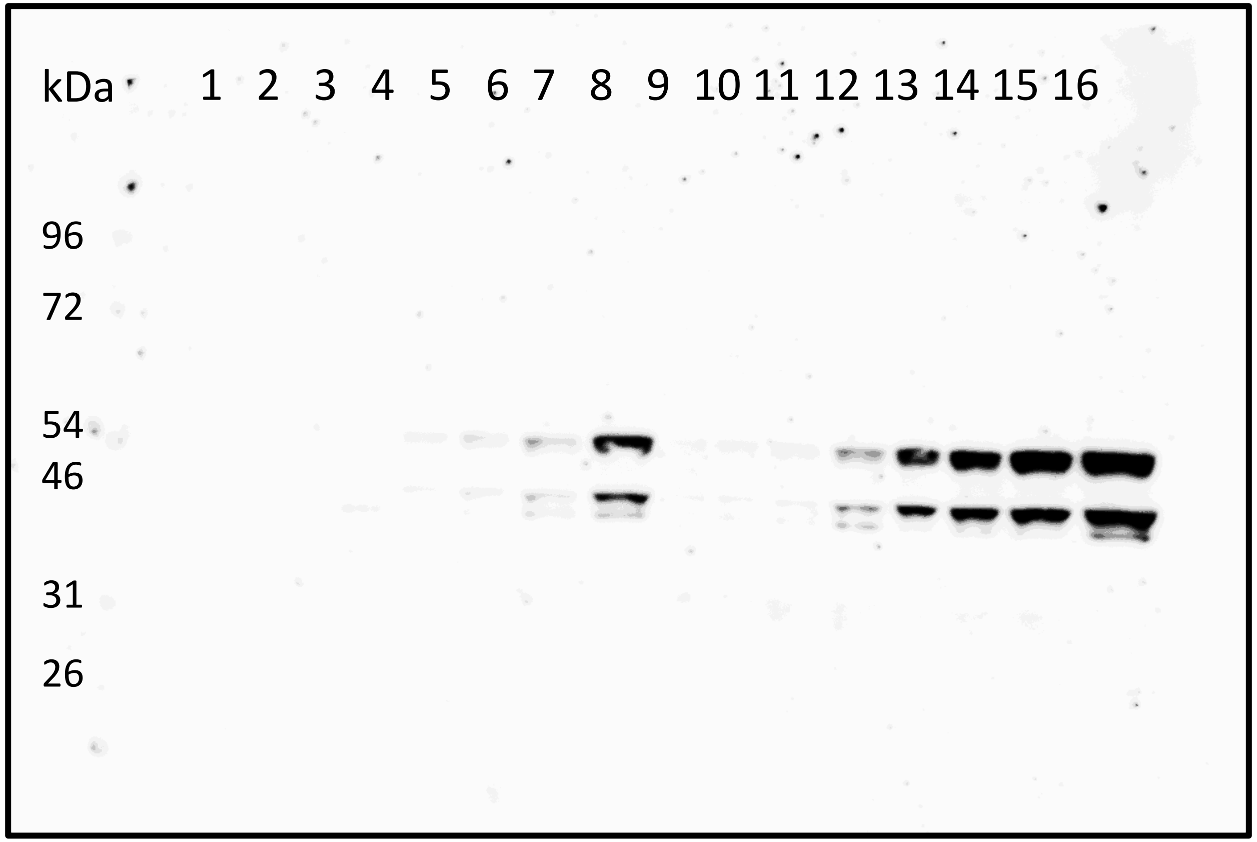

54kDa: 0:0:0:0:0:0:1:29:0:0:0:3:19:58:87:100

46kDa: 0:0:0:0:0:0:1:15:0:0:0:3:12:28:49:70

HT1080 6 h 12 h treatment JNK

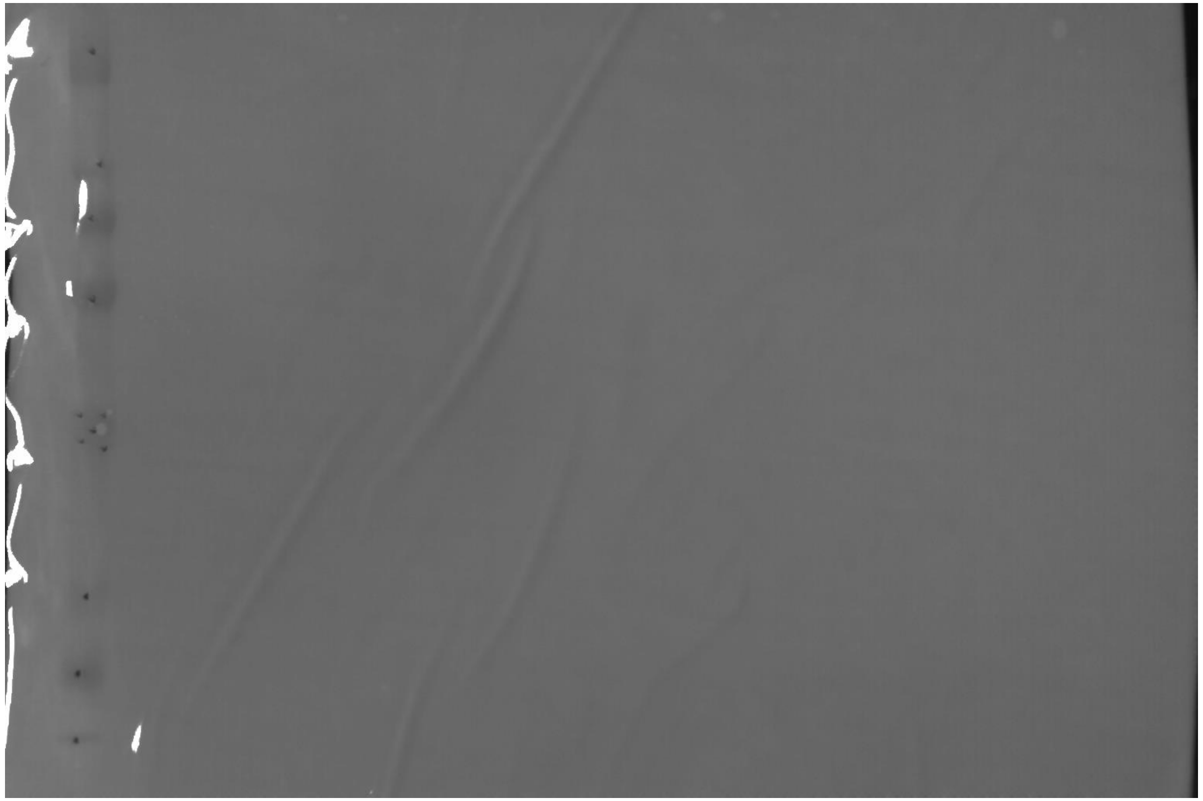

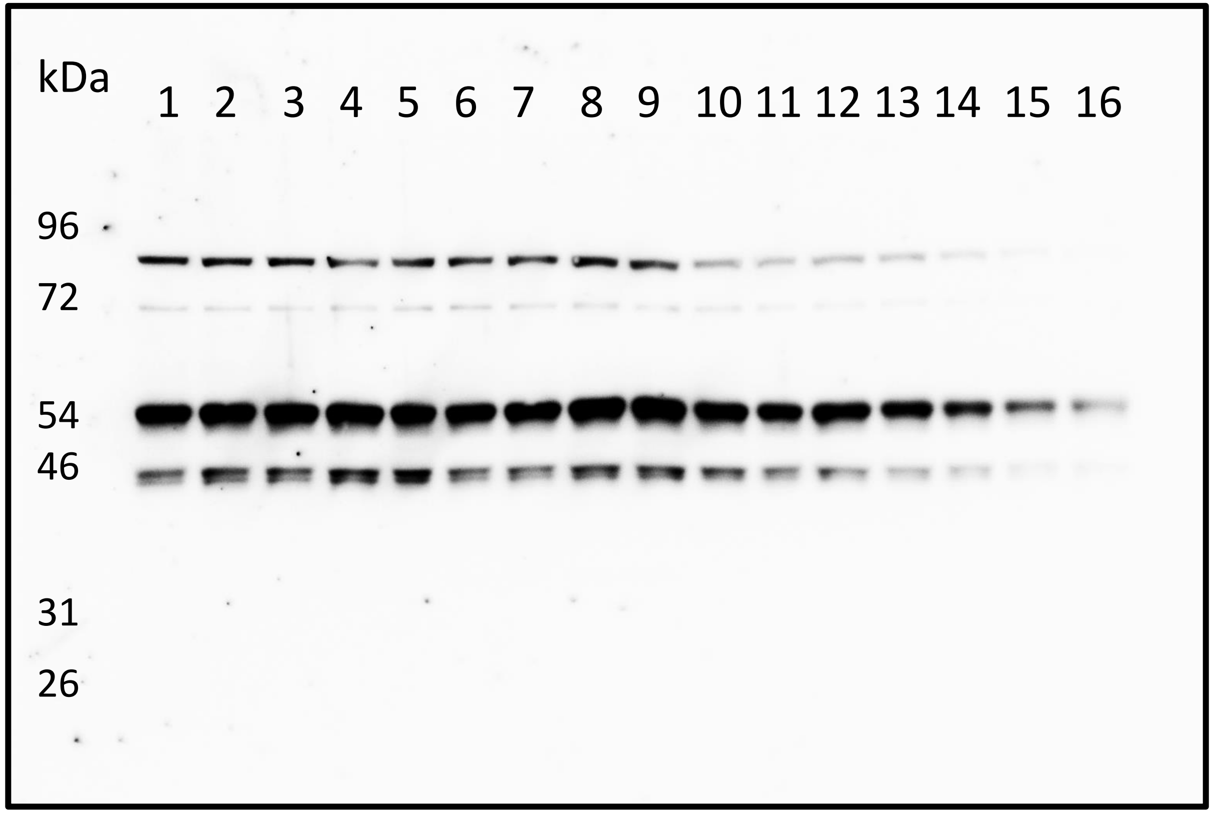

54kDa:100:111:83:114:70:63:90:108:119:93:51:80:49:29:12:4

46kDa:13:22:17:28:23:11:10:18:17:12:6:6:2:1:0:0

HT1080 6 h 12 h treatment p-p38

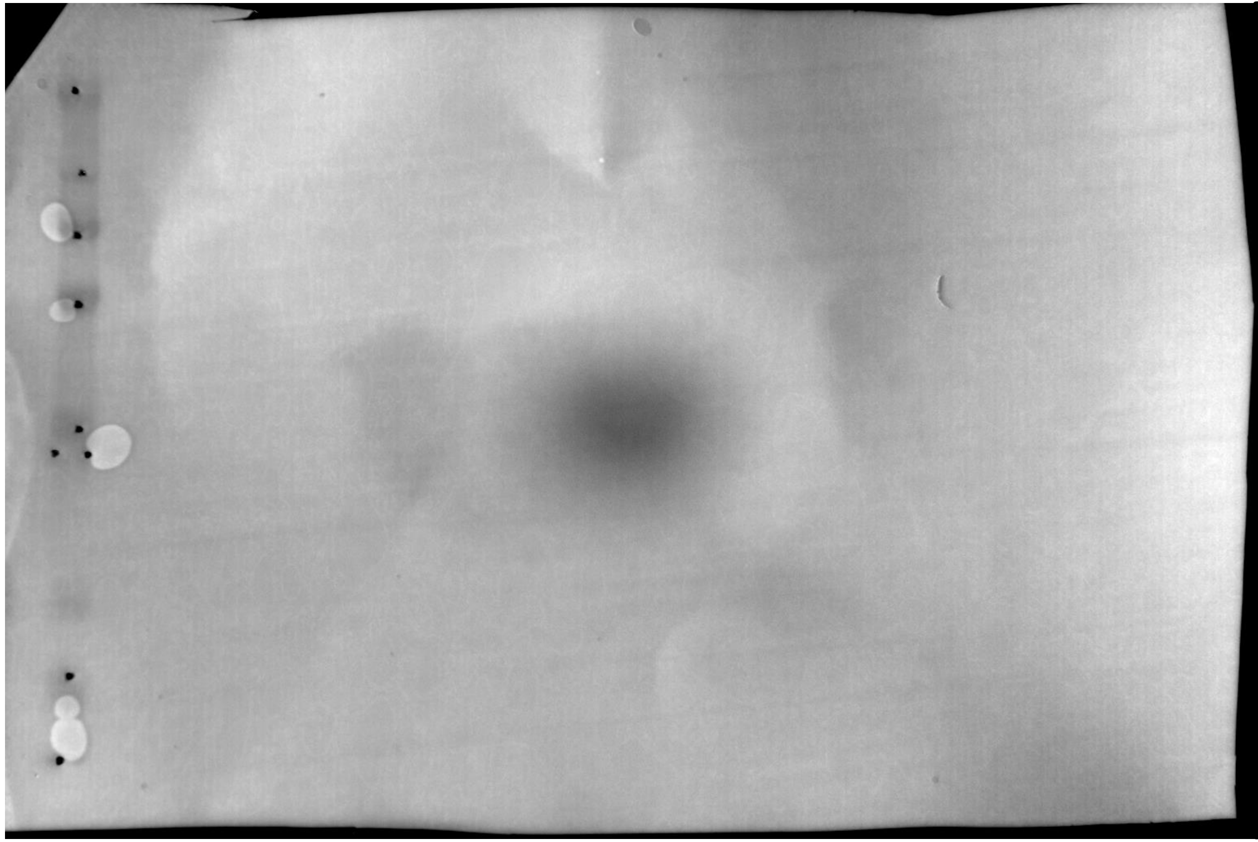

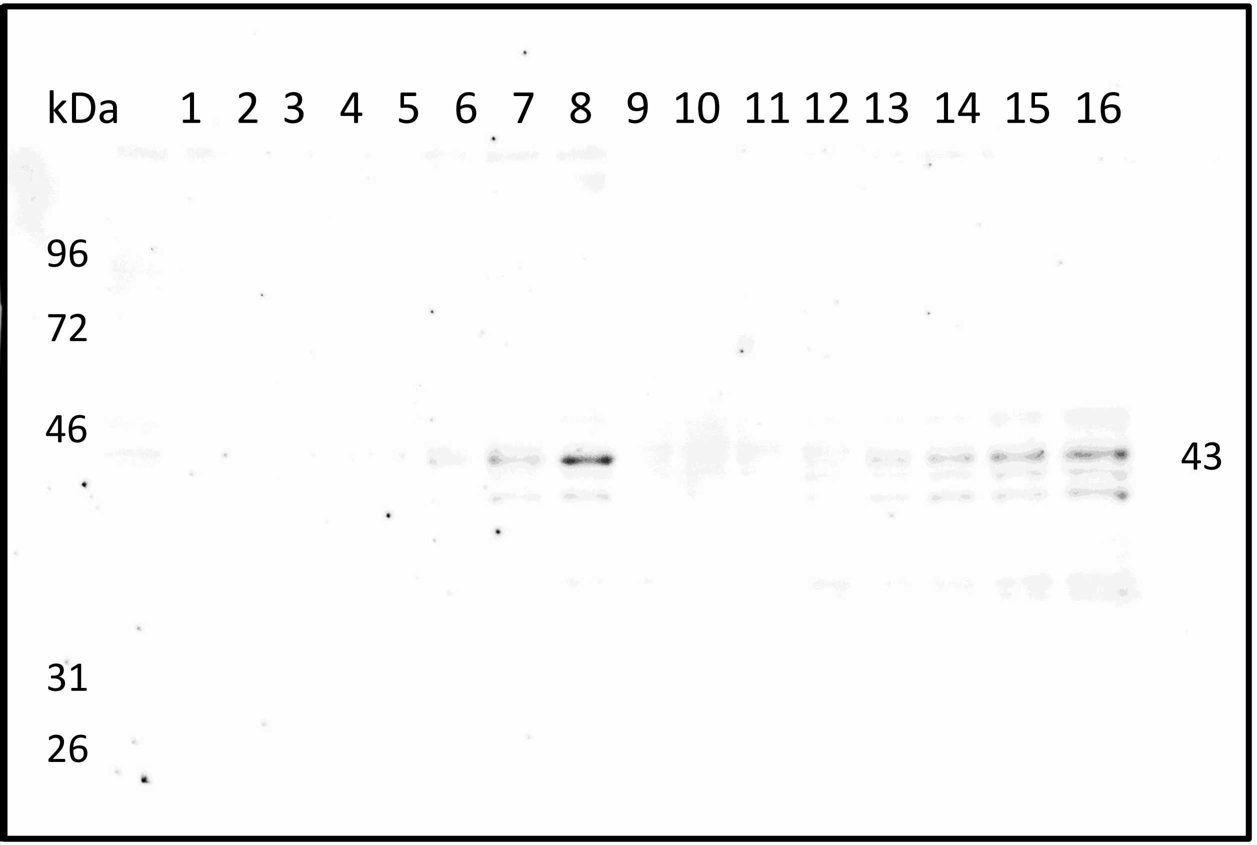

43kDa:0:2:0:0:0:15:50:171:13:39:12:8:17:26:51:100

HT1080 6 h 12 h treatment p38

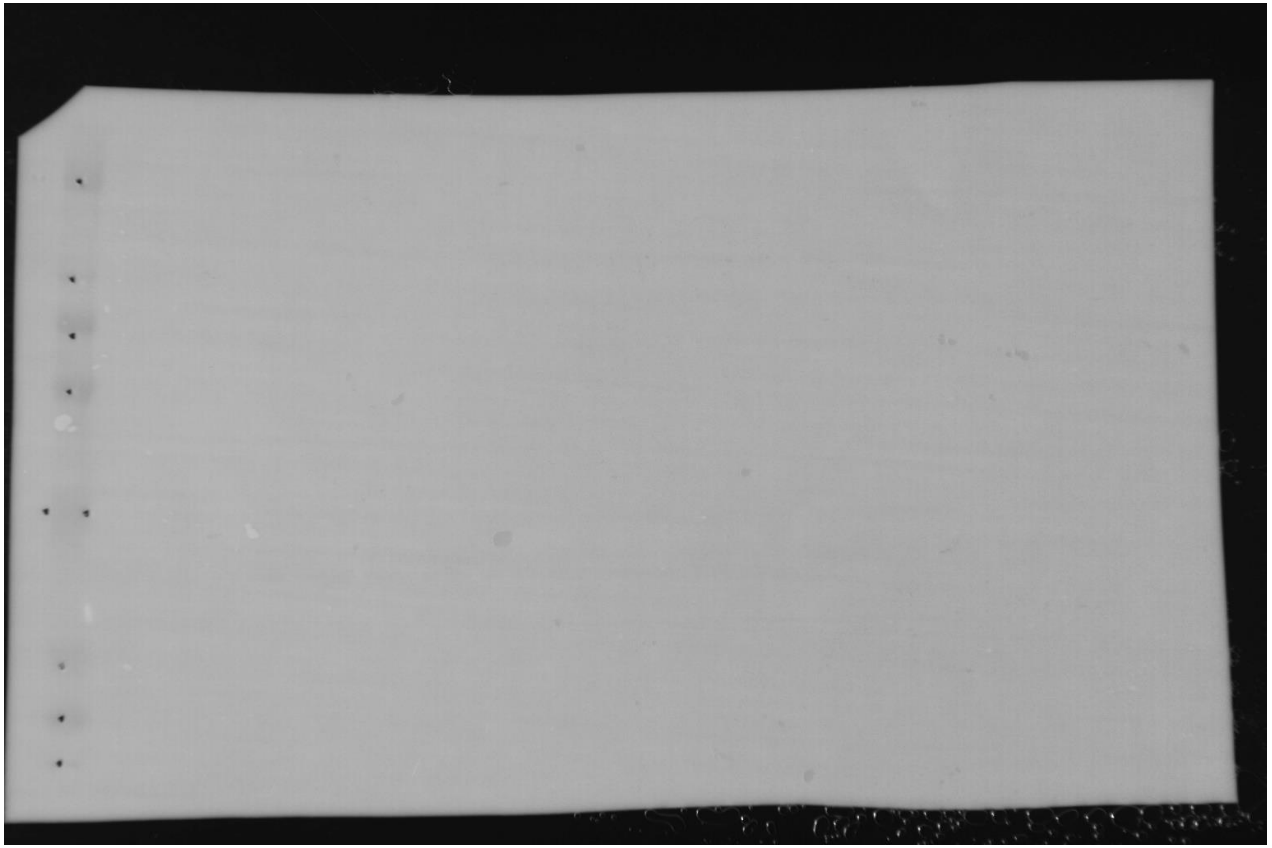

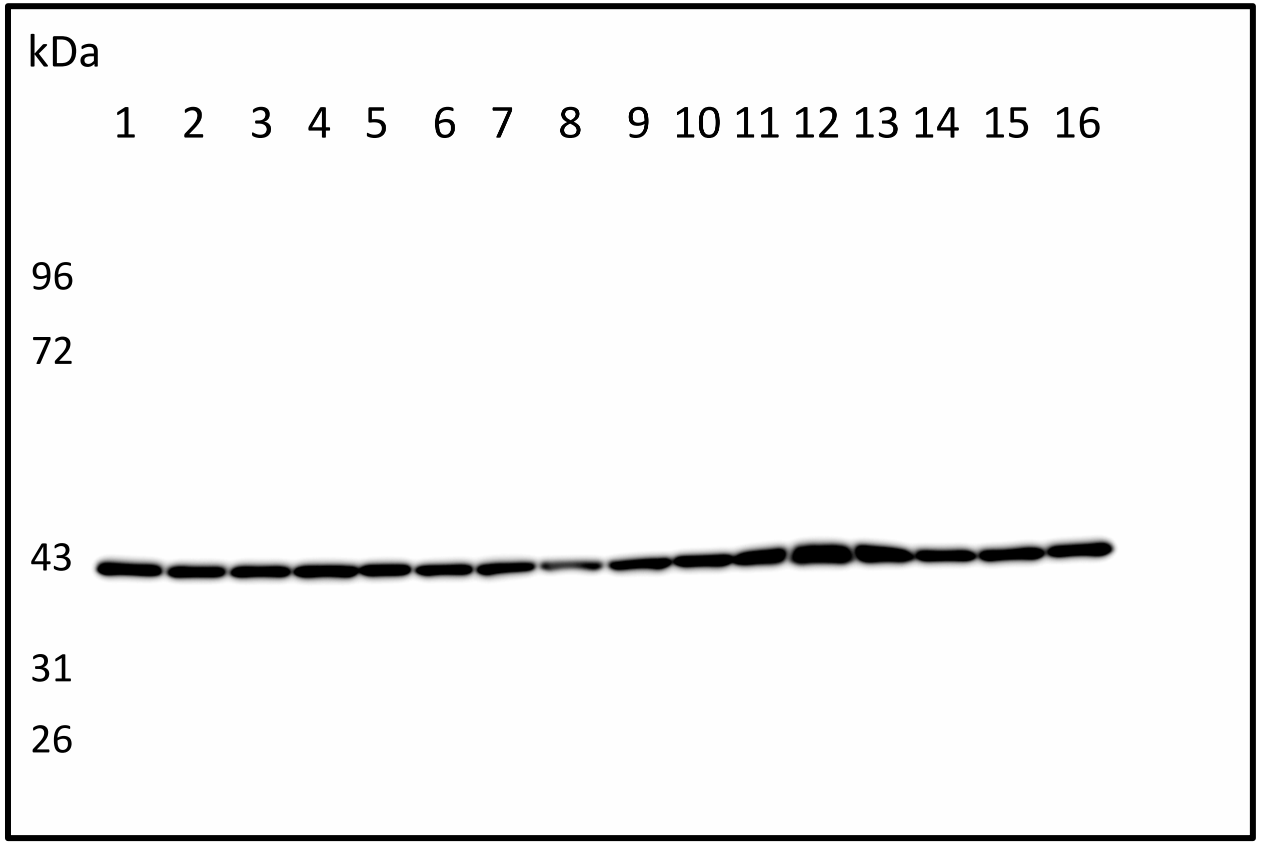

43kDa:100:77:71:101:80:61:66:24:75:88:90:111:98:89:92:93

HT1080 6 h 12 h treatment MYC

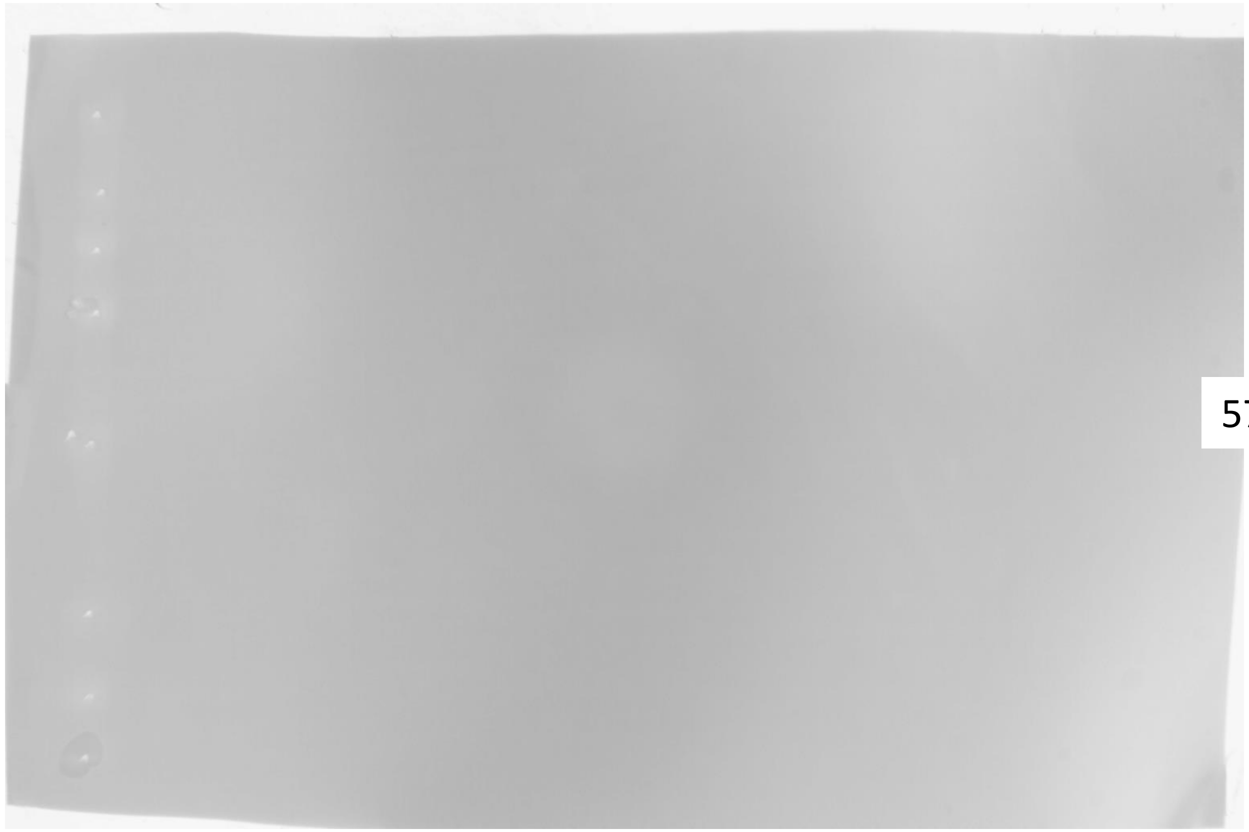

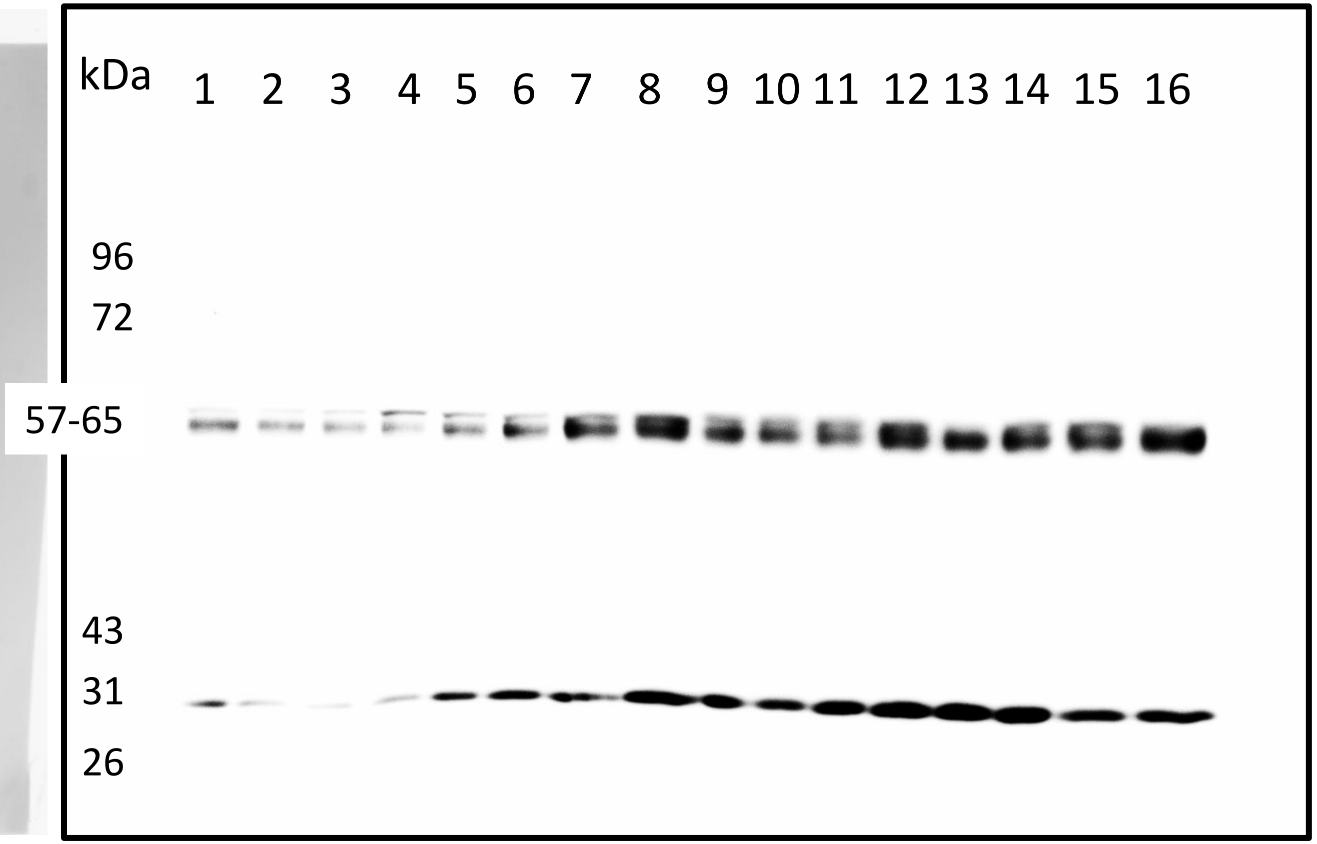

57-65kDa:100:43:31:56:93:166:412:696:327:278:295:700:458:524:601:928

HT1080 6 h 12 h treatment Survivin

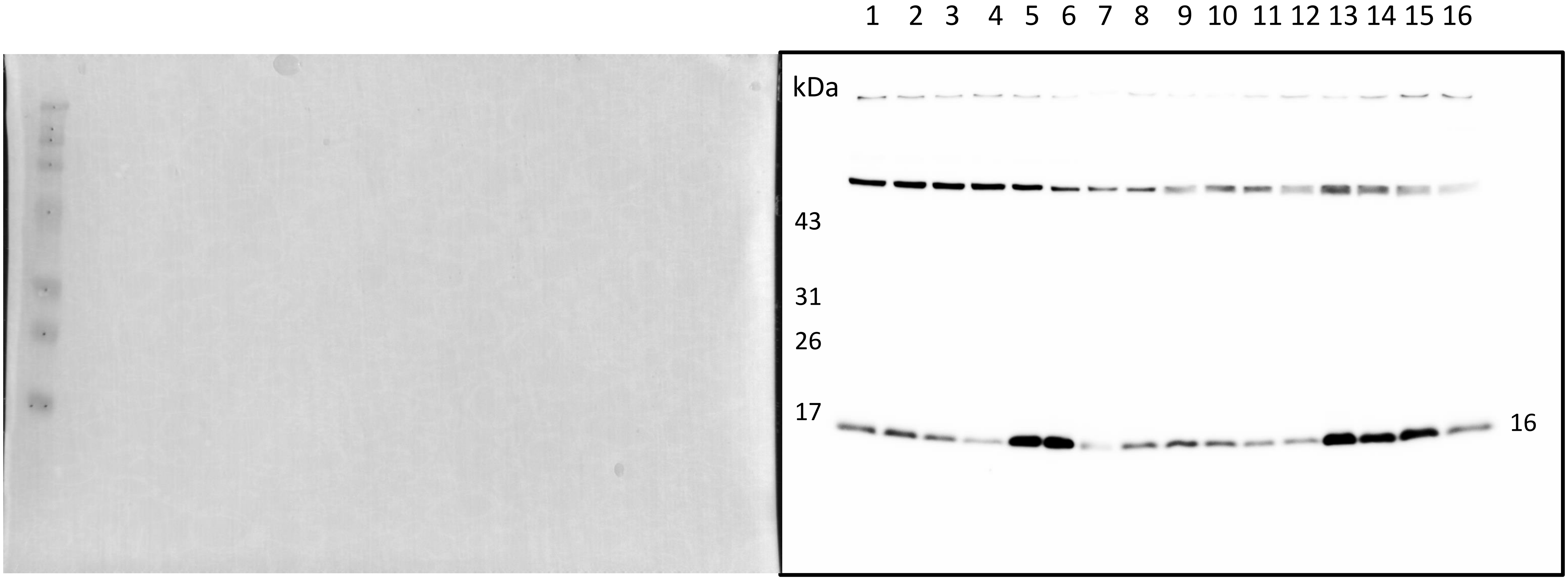

16kDa:100:143:82:28:610:652:21:85:124:87:44:47:631:571:492:84

HT1080 6 h 12 h treatment MCL-1

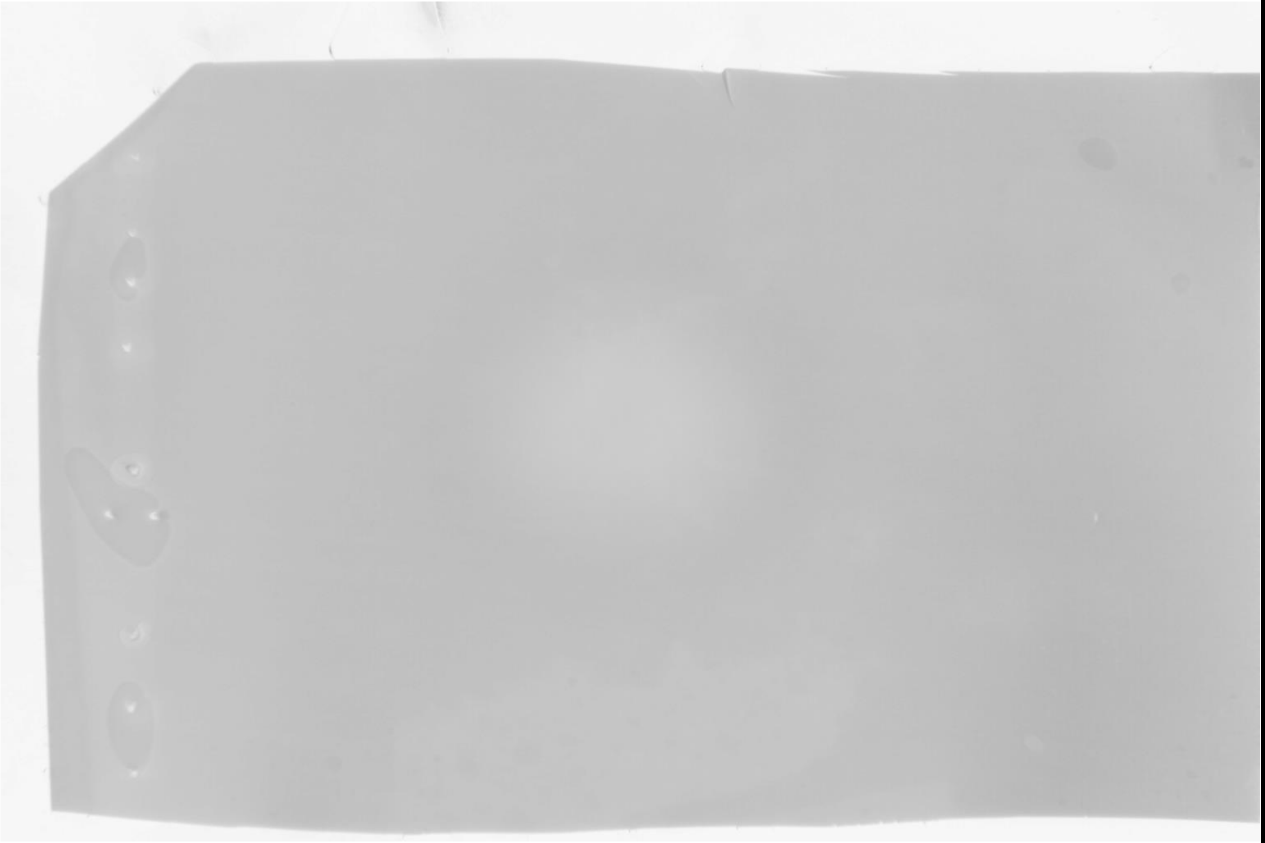

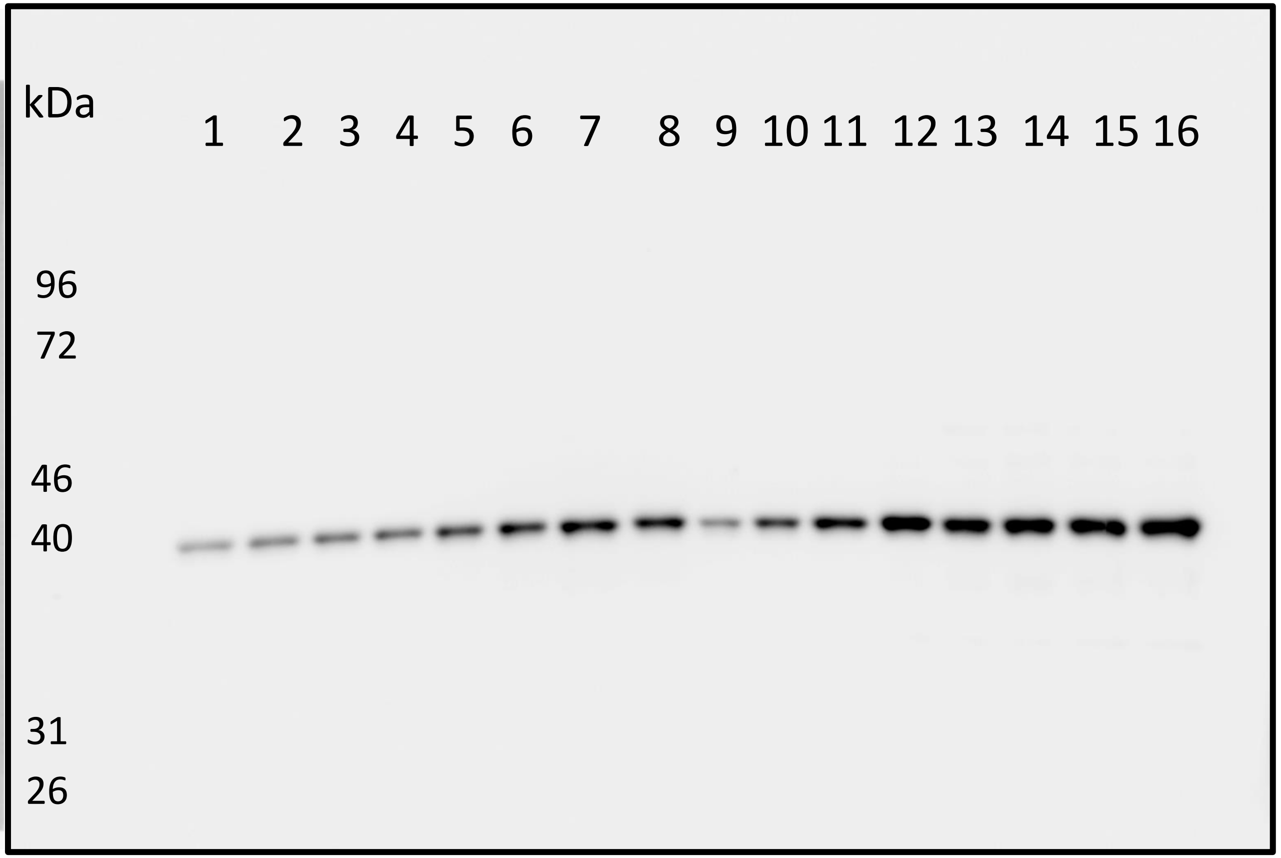

40kDa:5:8:9:11:16:21:33:27:7:17:39:62:51:57:70:100

HT1080 6 h 12 h treatment p-AKT

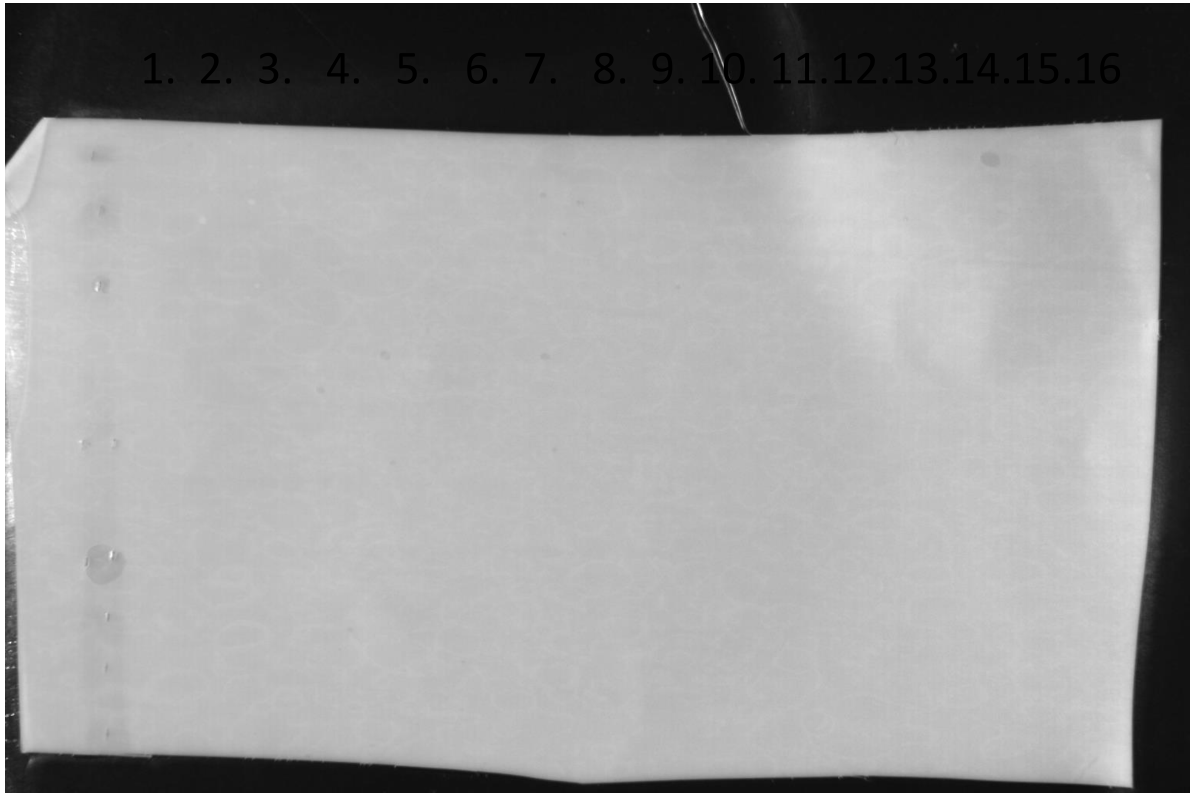

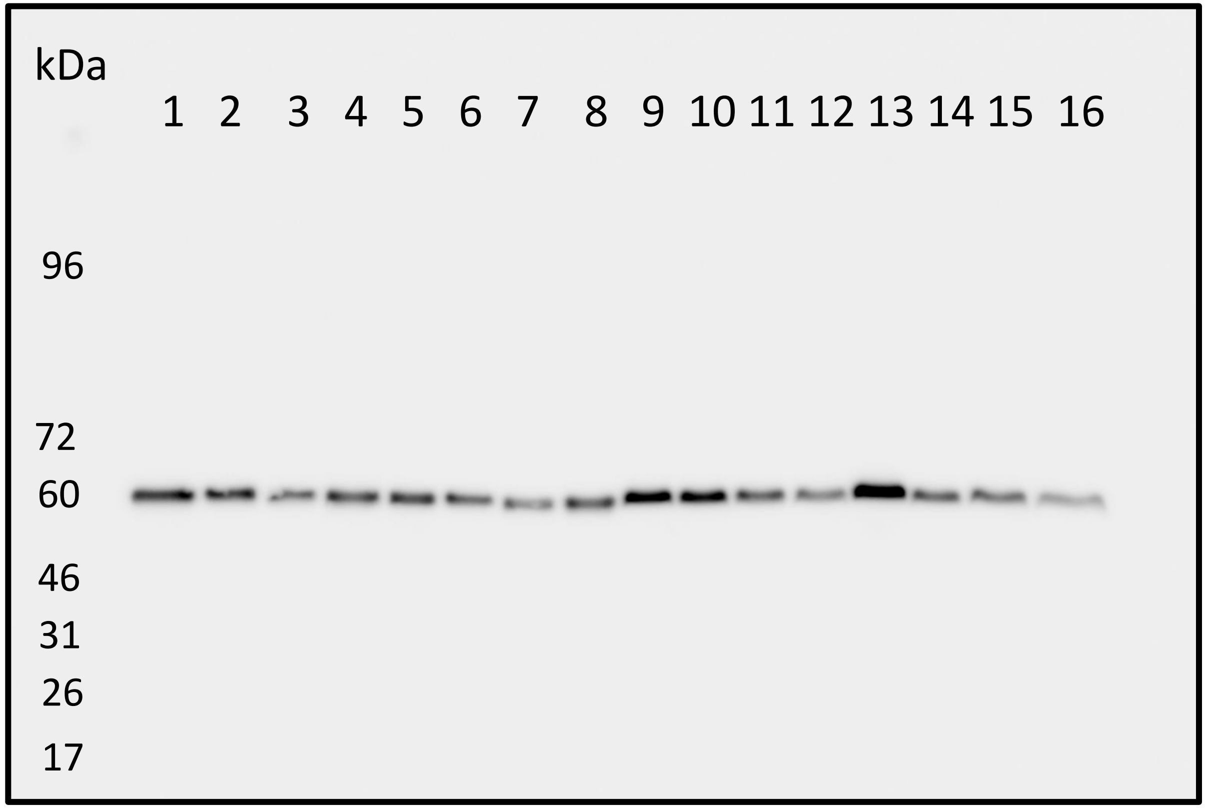

60kDa:100:78:38:64:64:49:35:60:140:120:58:40:215:54:46:25

HT1080 6 h 12 h treatment AKT

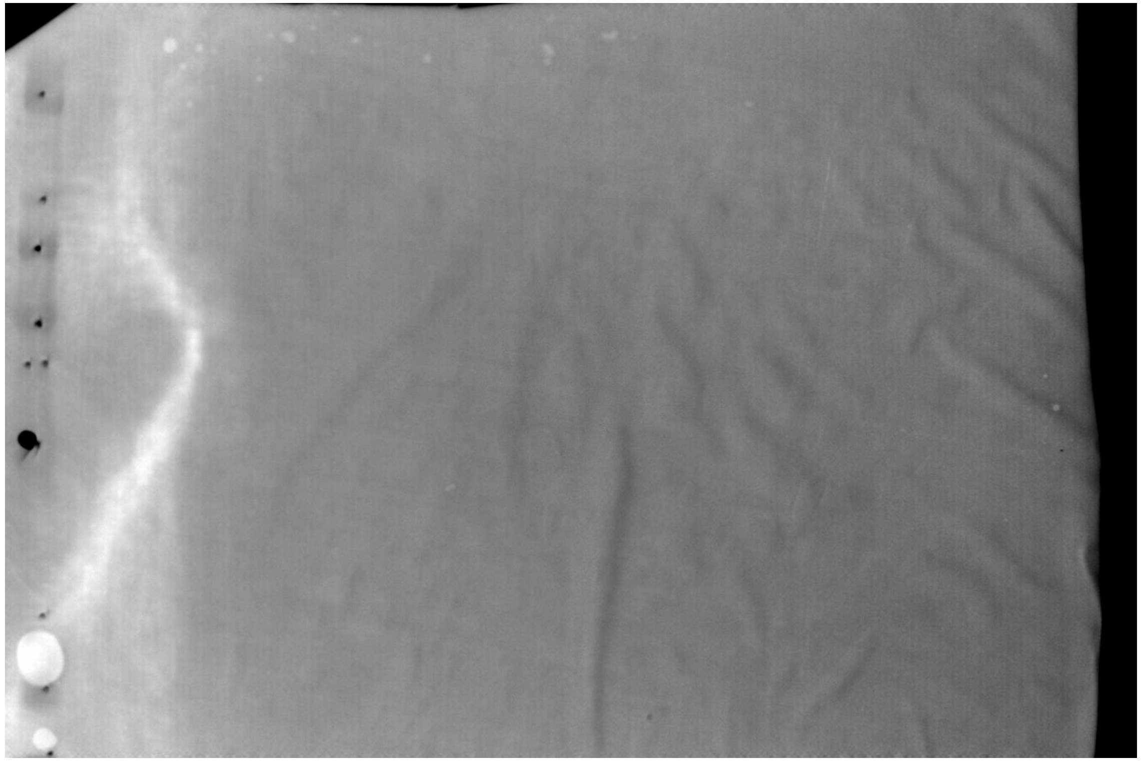

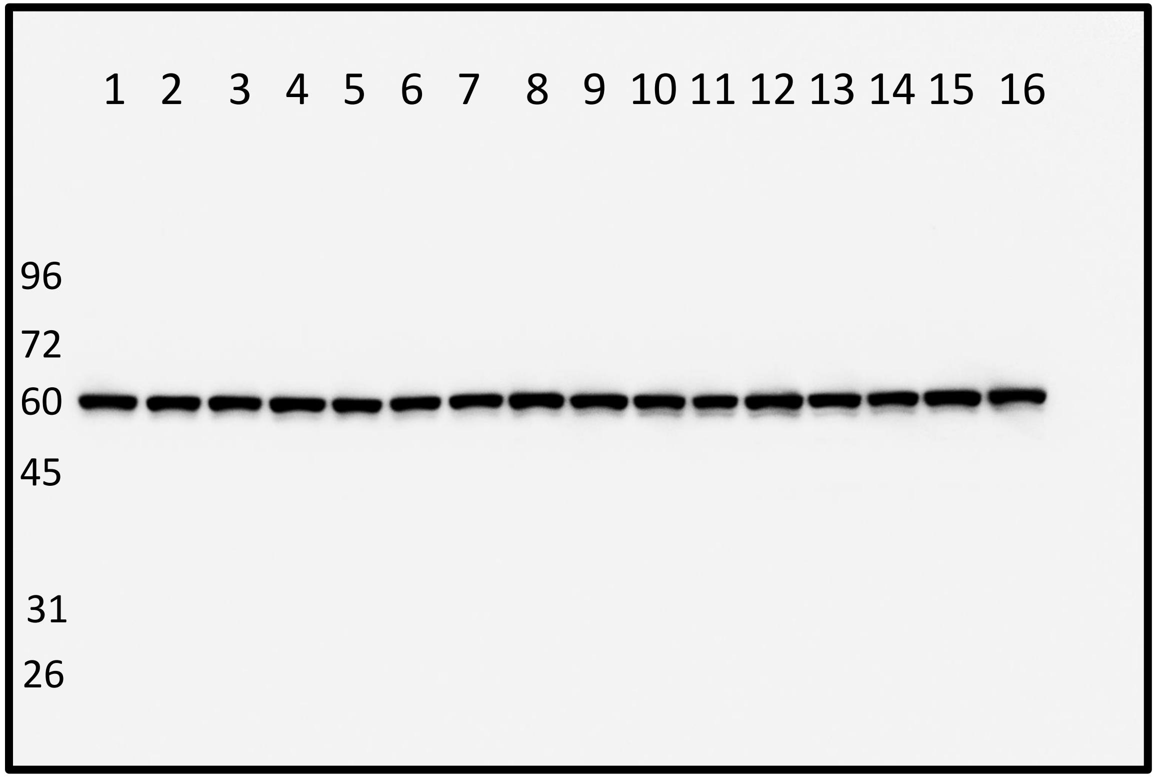

60kDa:100:91:70:82:60:58:80:80:100:68:52:106:94:81:114:82

HT1080 treatment 24h

Treatment time 24 h

- 1. Everolimus 0 µM
- 2. Everolimus 5 µM
- 3. Everolimus 10 µM
- 4. Everolimus 20 µM
- 5. Everolimus 0 µM Bortezomib 5nM
- 6. Everolimus 5 µM Bortezomib 5nM
- 7. Everolimus 10 µM Bortezomib 5nM
- 8. Everolimus 20 µM Bortezomib 5nM

HT1080 24 h treatment β actin

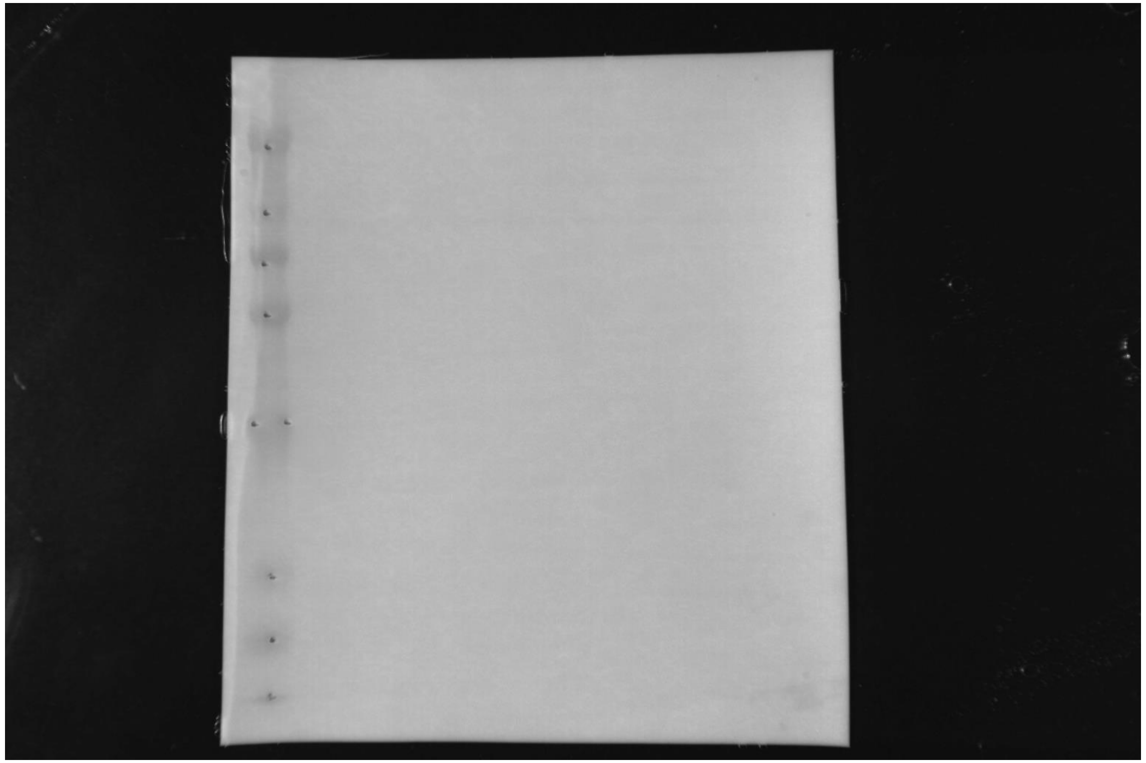

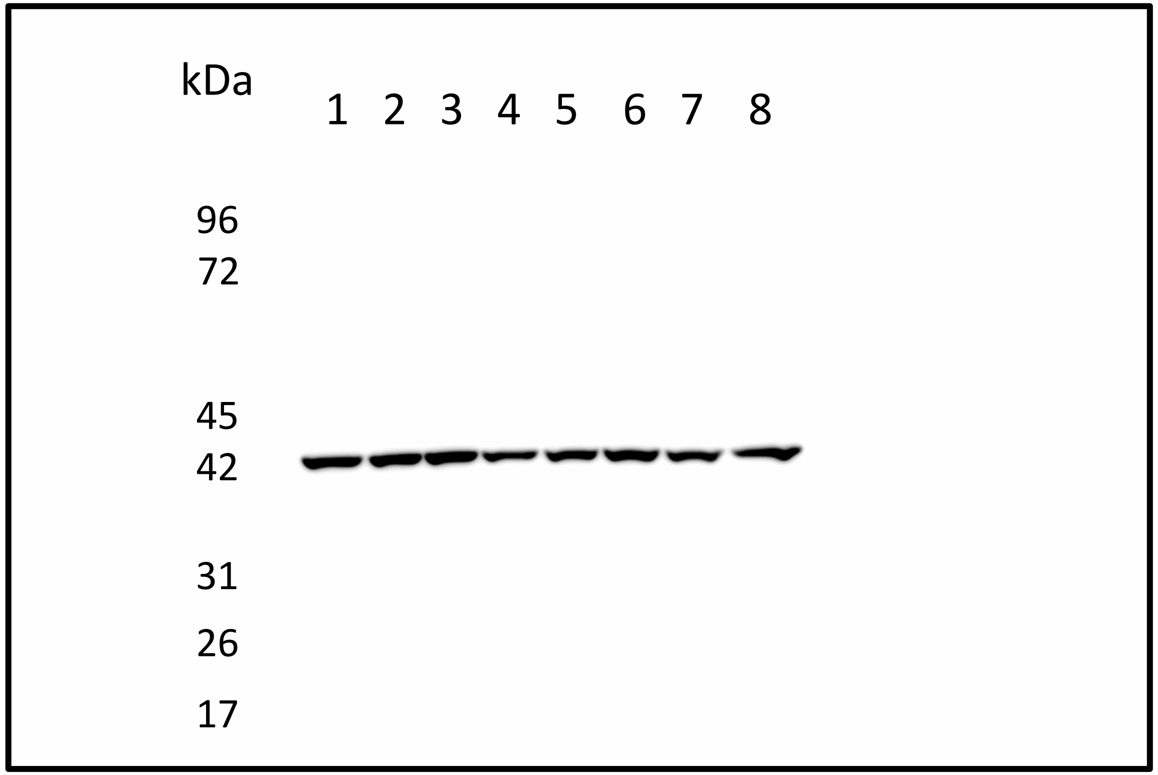

42kDa:100:74:81:49:52:68:53:67

HT1080 24 h treatment BIK

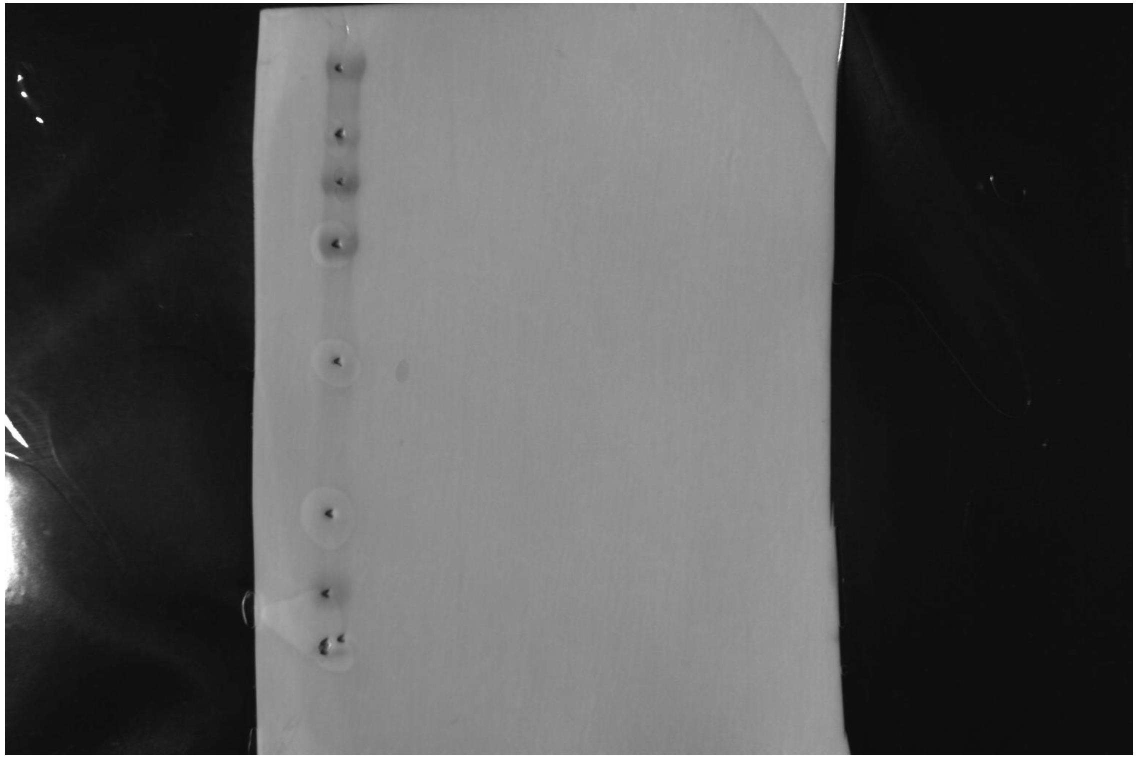

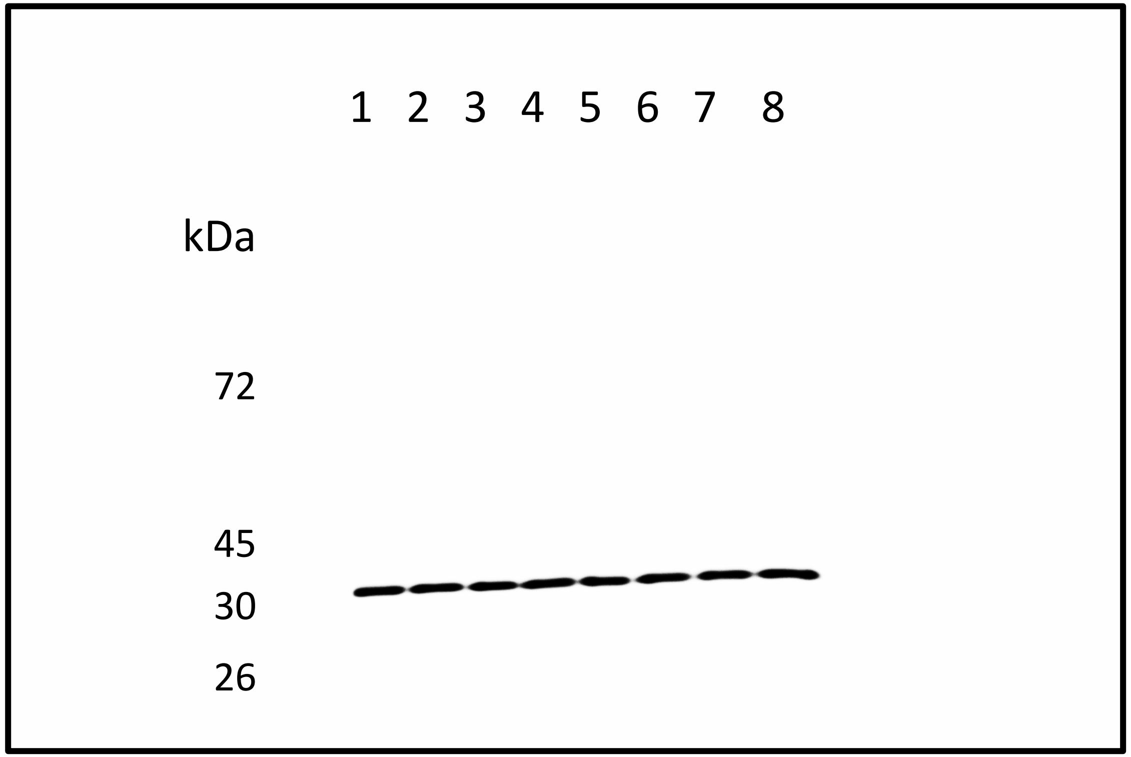

30kDa:100:121:94:116:110:108:119:183

HT1080 24 h treatment cleaved caspase 9

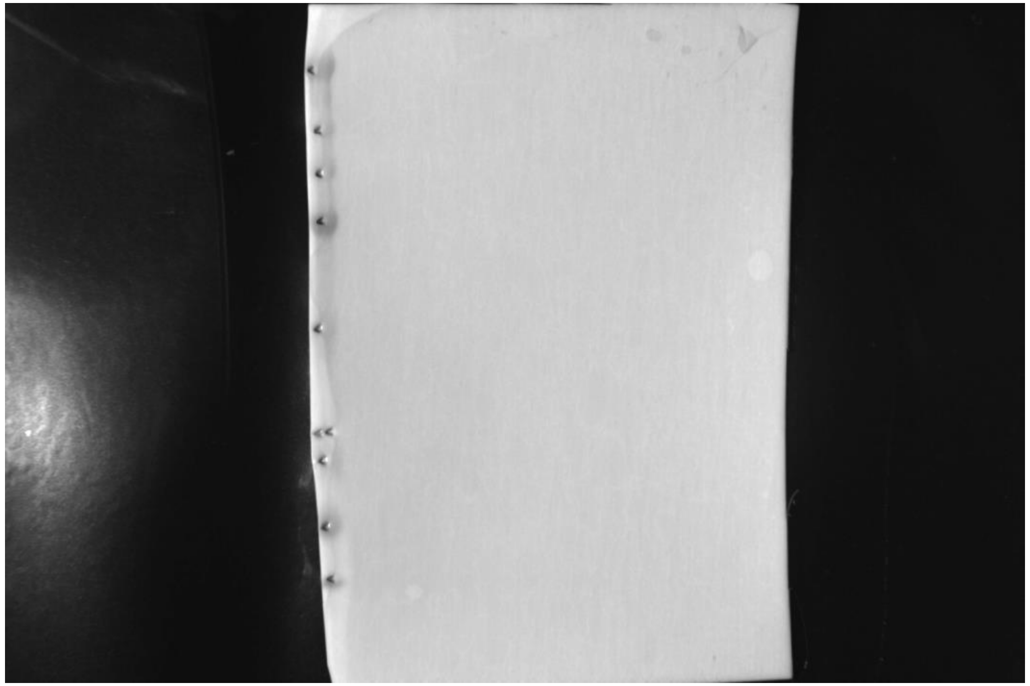

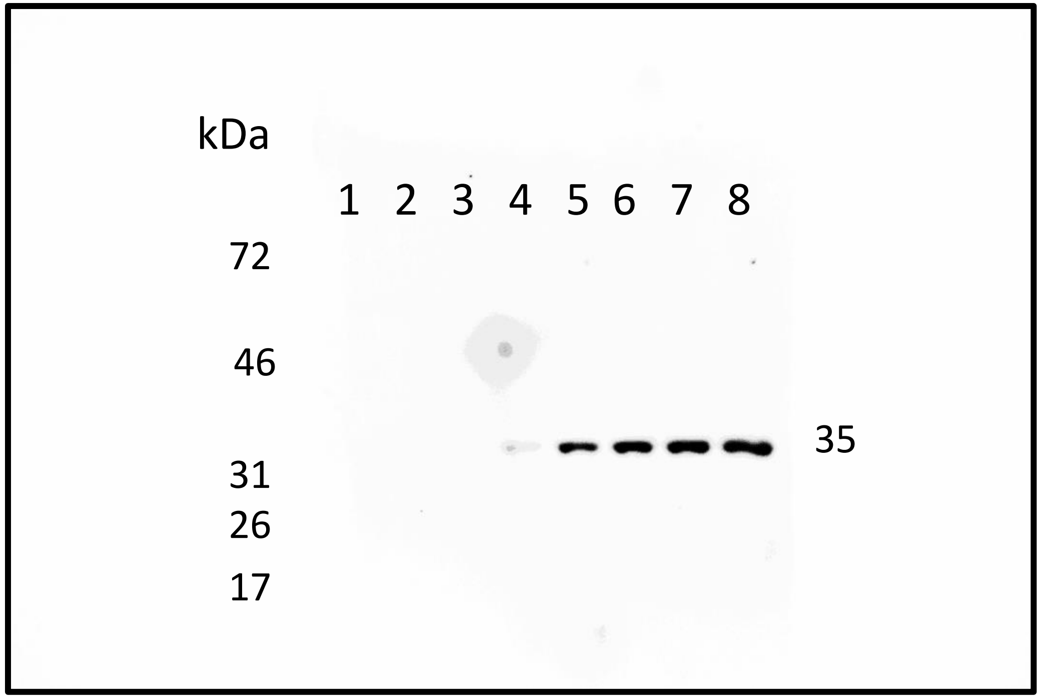

35kDa:0:0:0:1:38:59:82:100

HT1080 24h treatment cleaved caspase 8

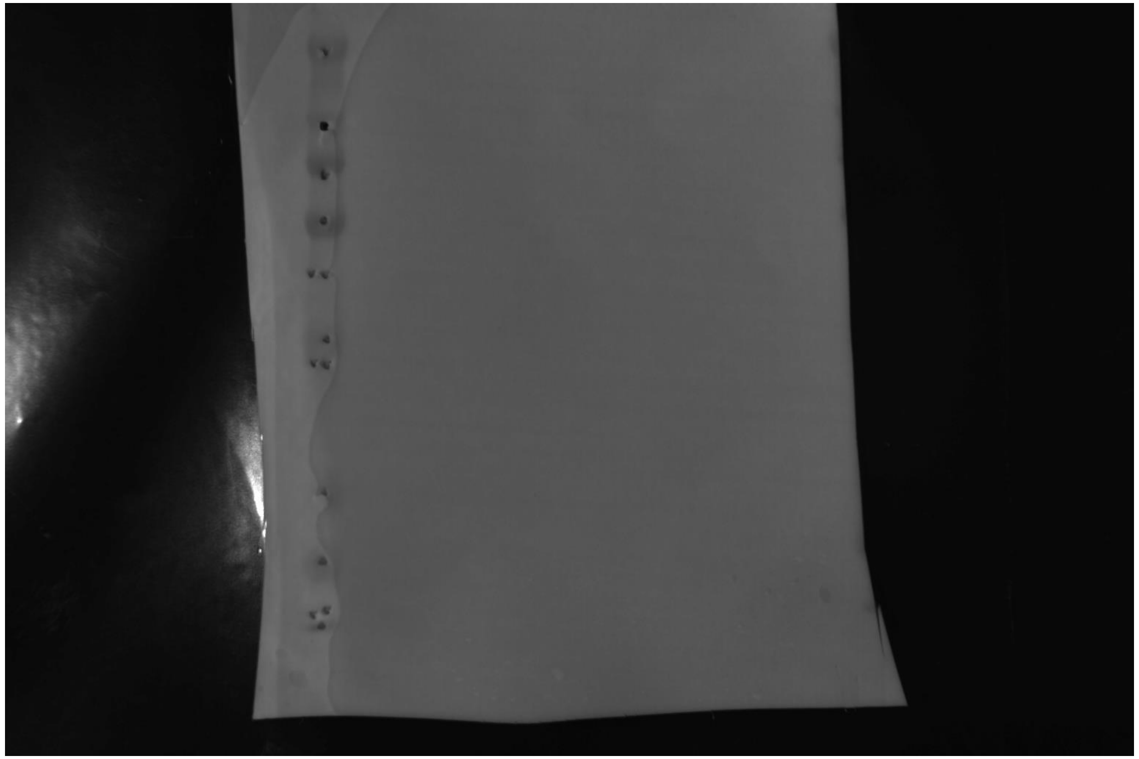

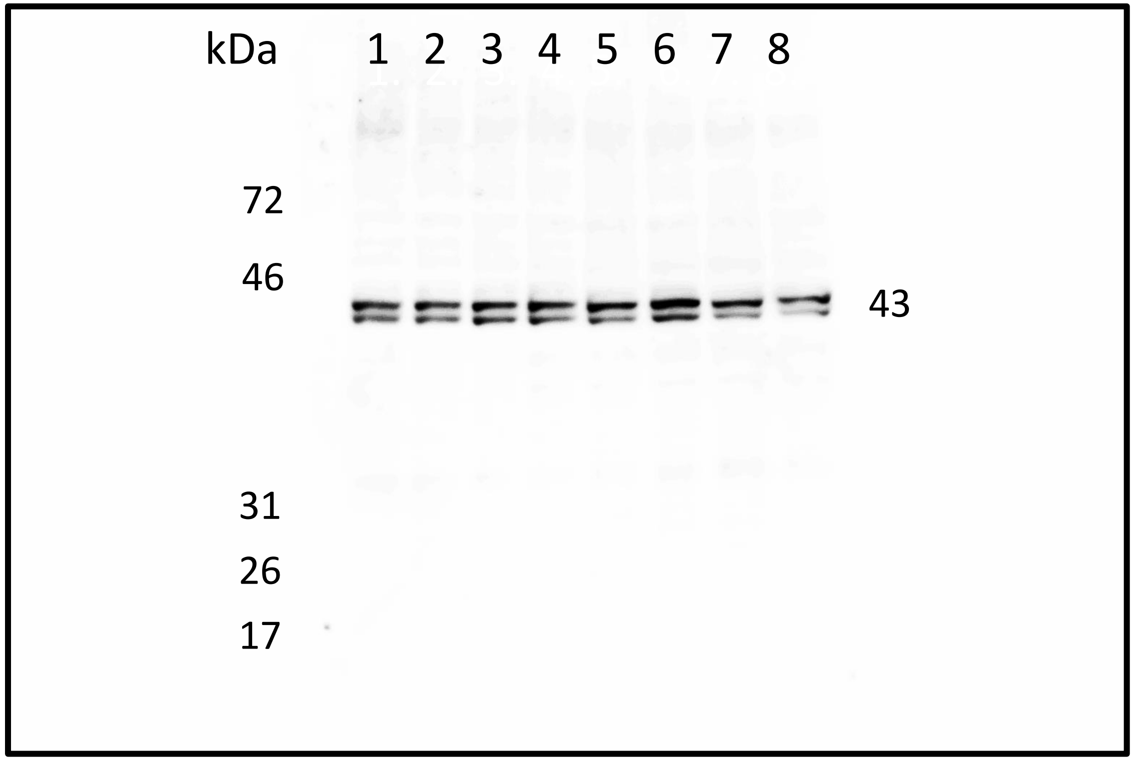

43kDa:100:80:105:105:122:174:95:66

HT1080 24h treatment cleaved caspase 3

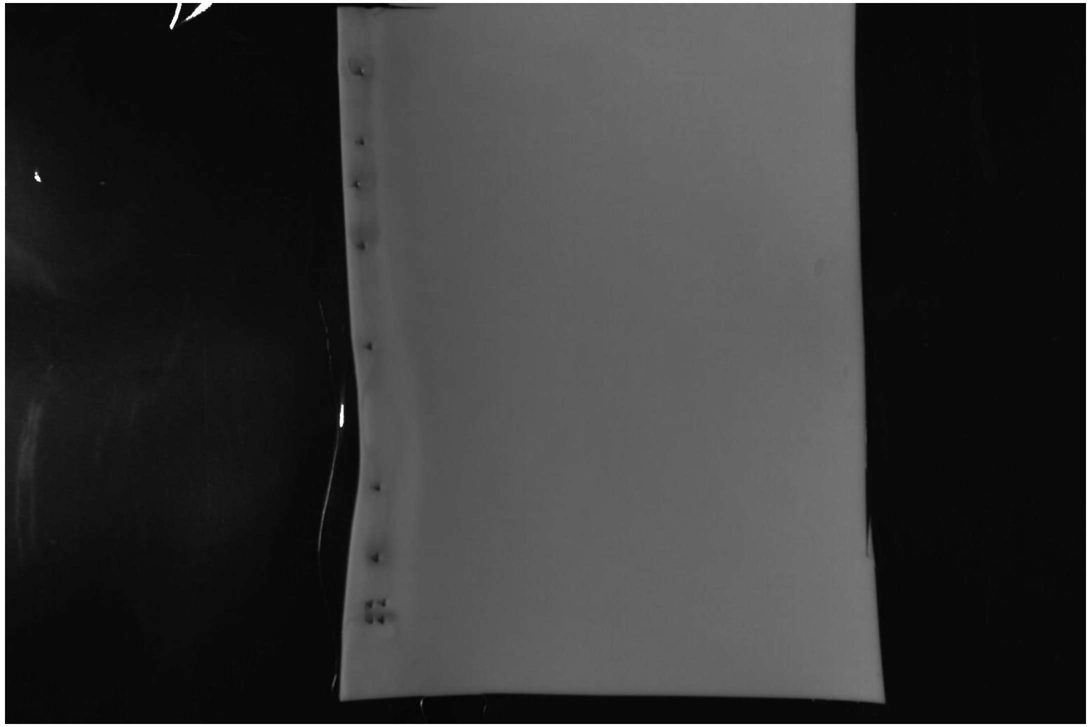

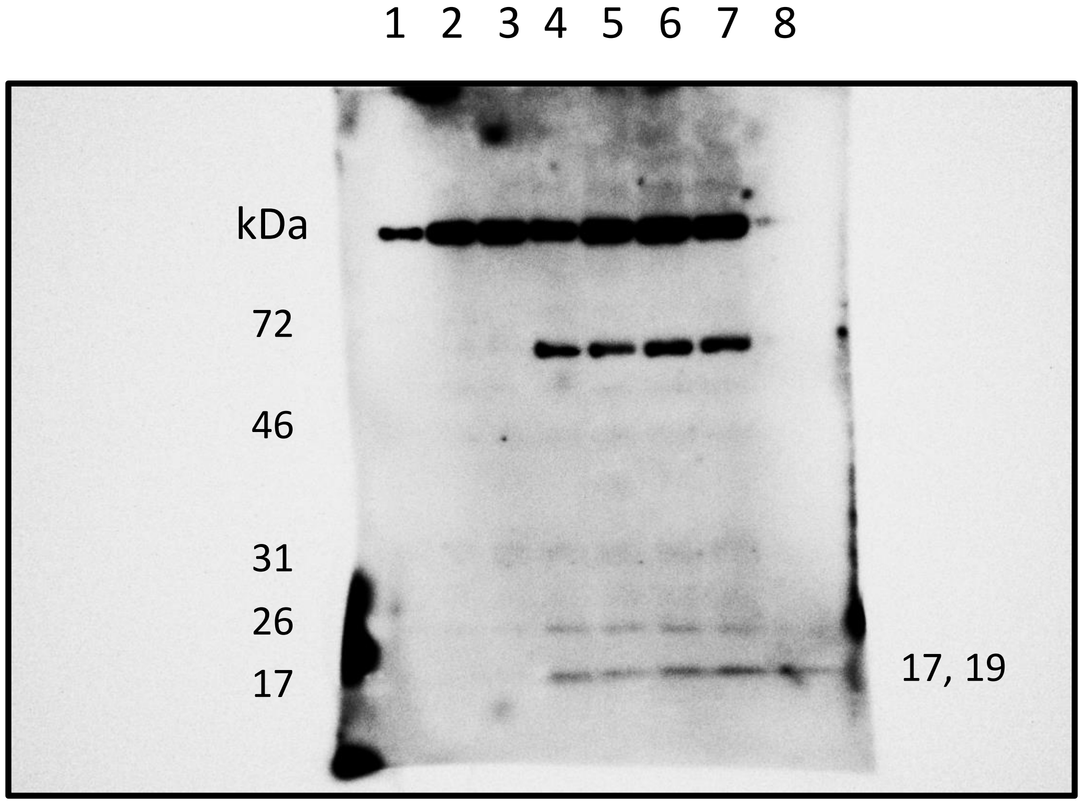

17,19kDa:0:3:4:52:39:86:102:100

HT1080 24h treatment cleaved PARP

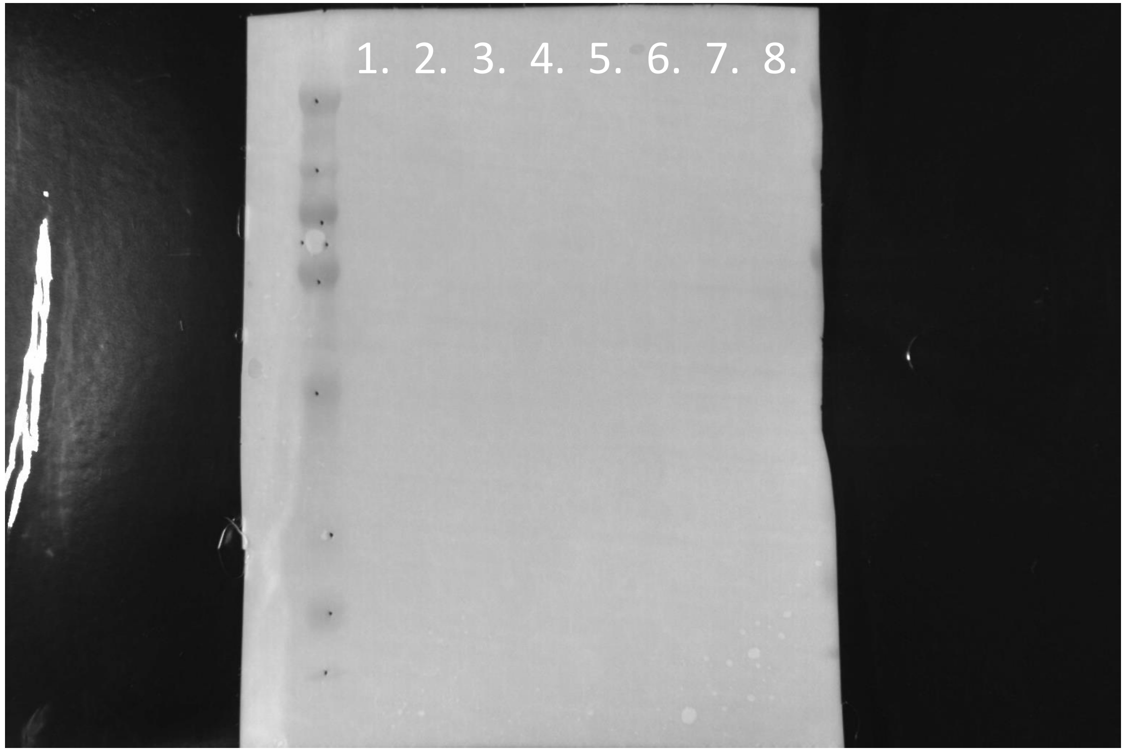

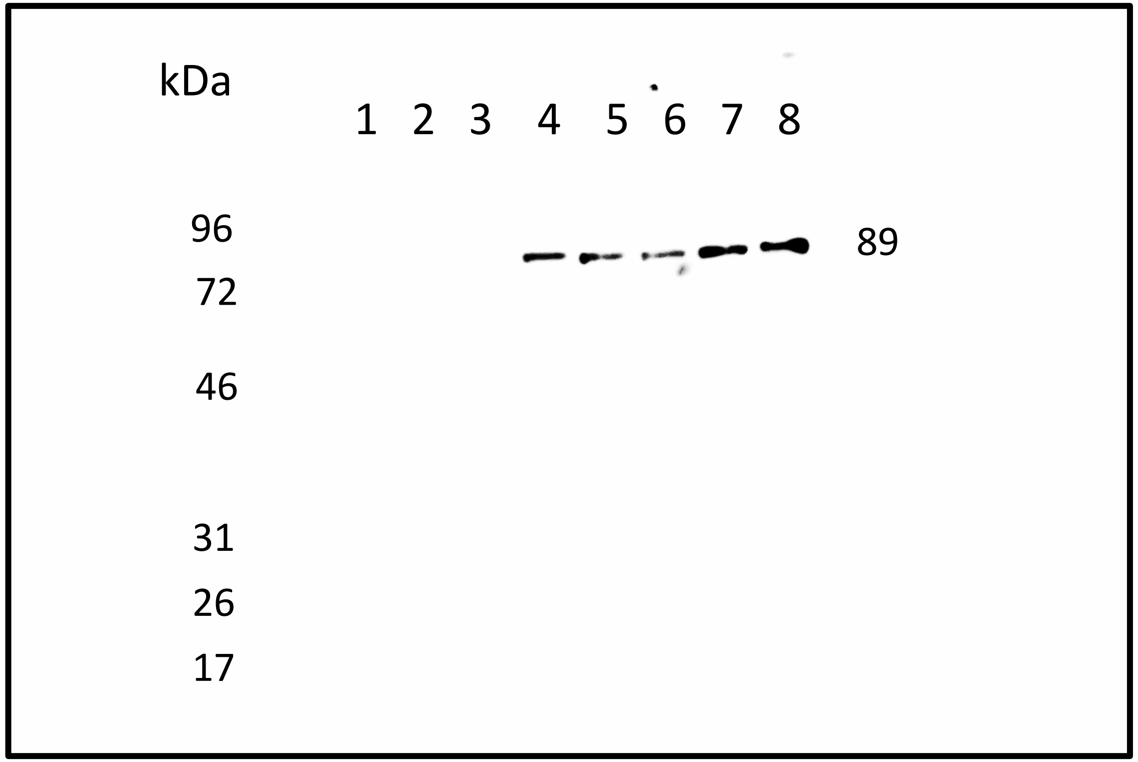

89kDa:0:0:0:35:27:15:88:100

HT1080 24h treatment p-ERK

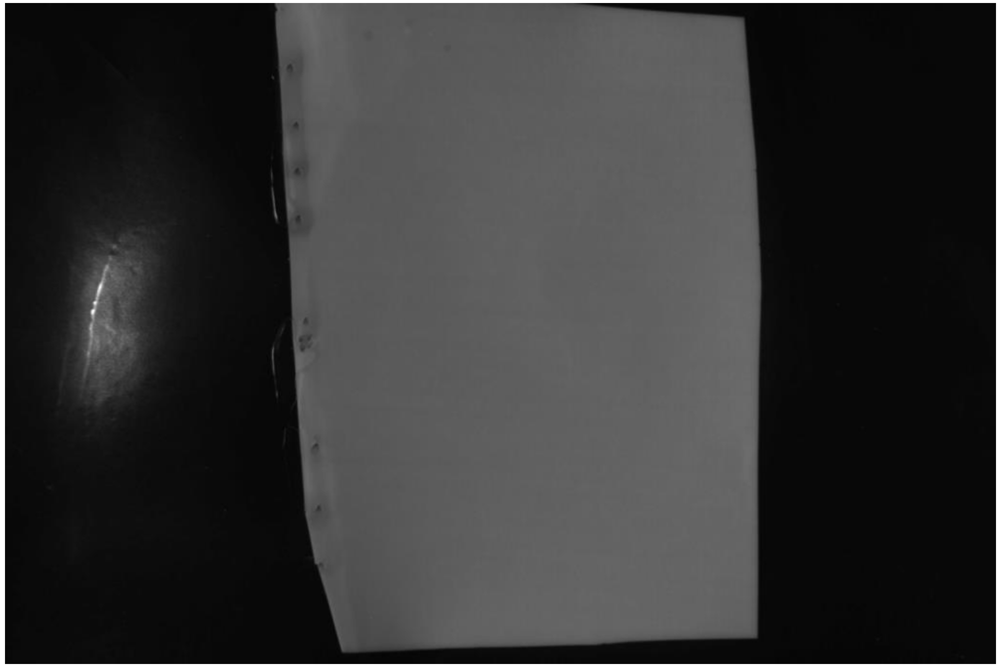

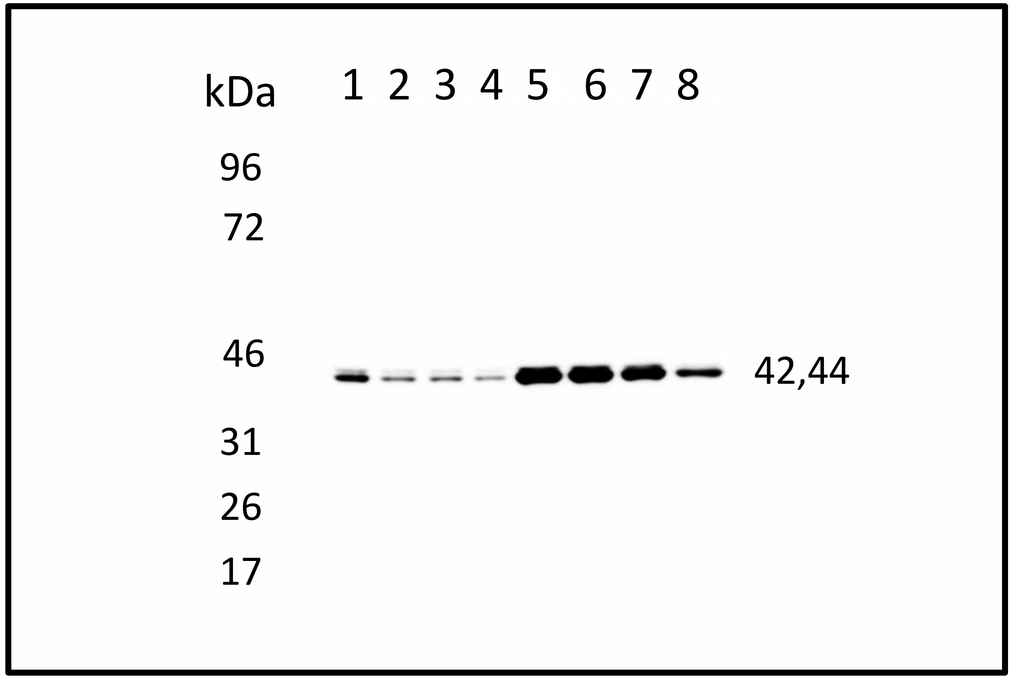

42.44kDa:100:28:29:14:601:563:359:155

HT1080 24h treatment ERK

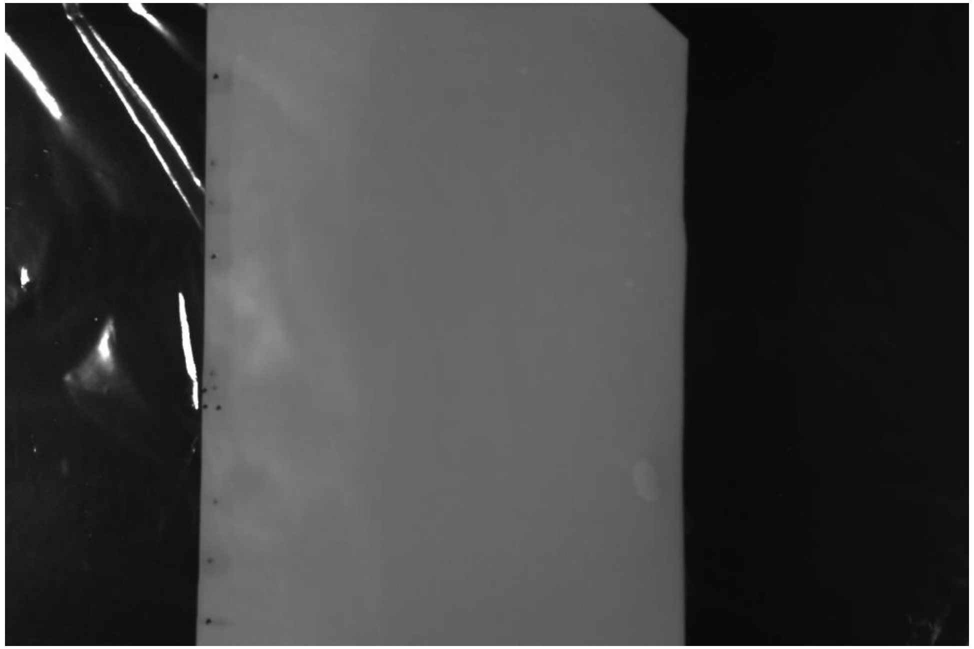

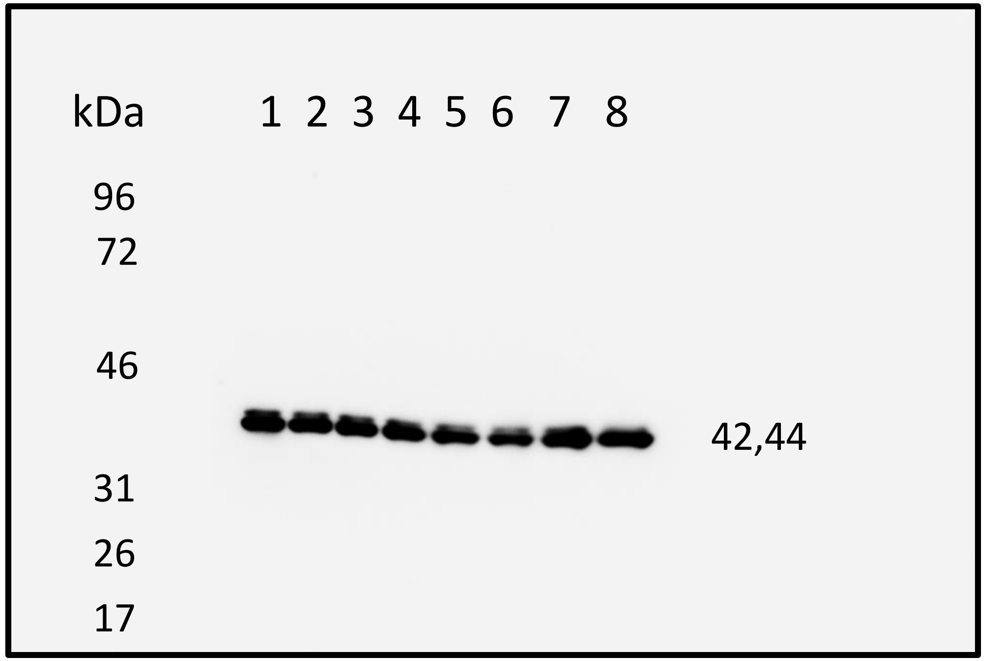

42.44kDa:100:80:84:74:68:62:103:104

HT1080 24h treatment p-JNK

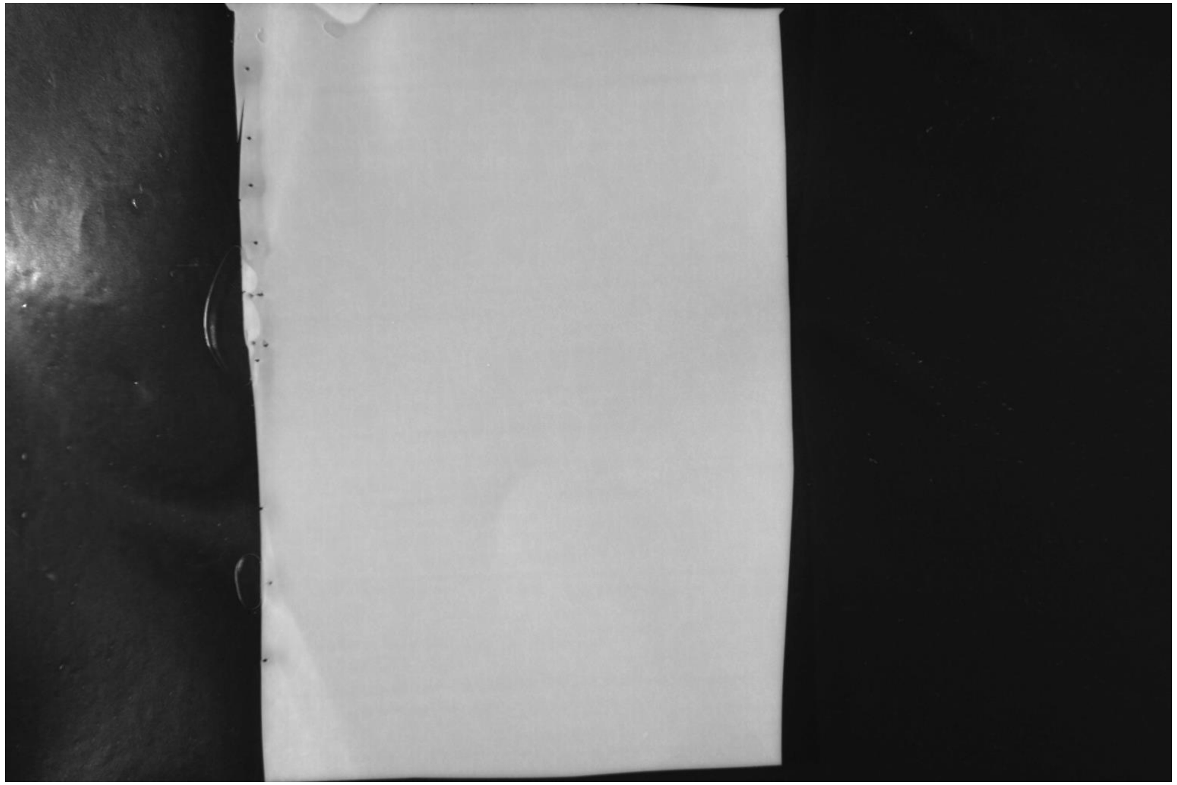

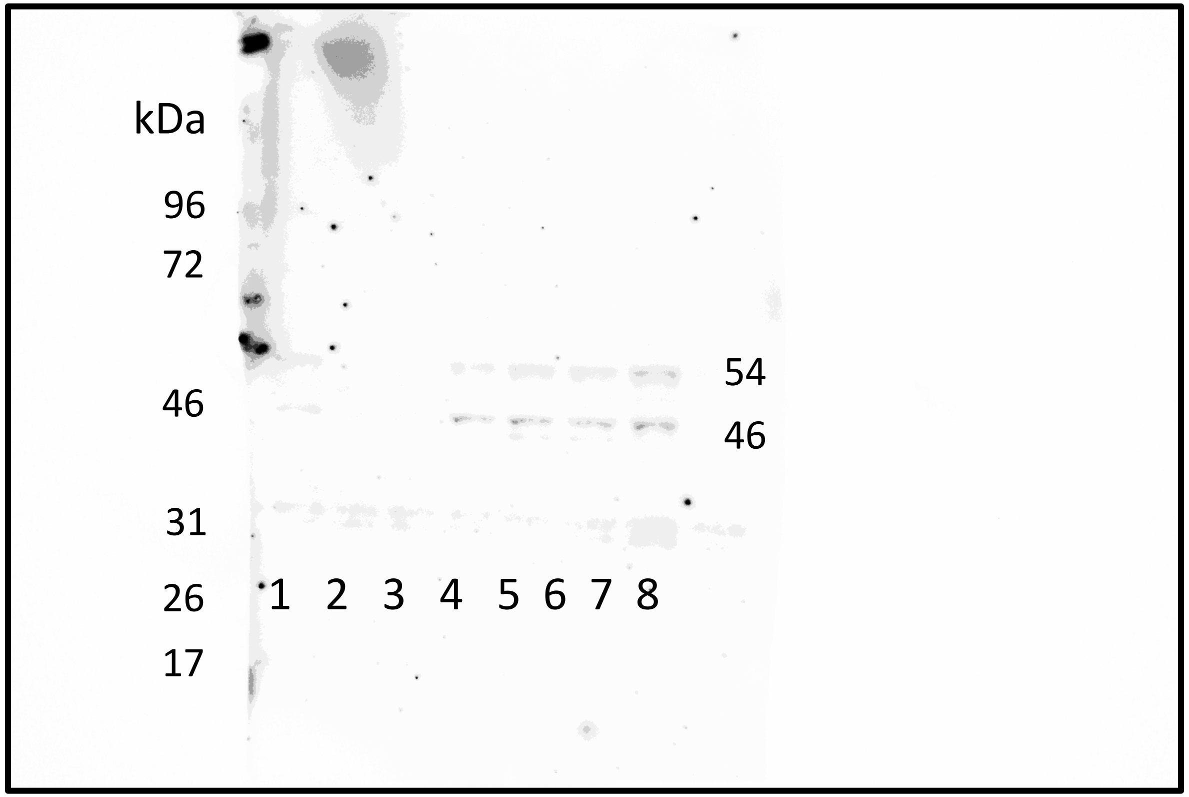

54kDa:100:115:82:122:115:159:164:47

46kDa:100:71:65:108:206:223:251:43

HT1080 24h treatment JNK

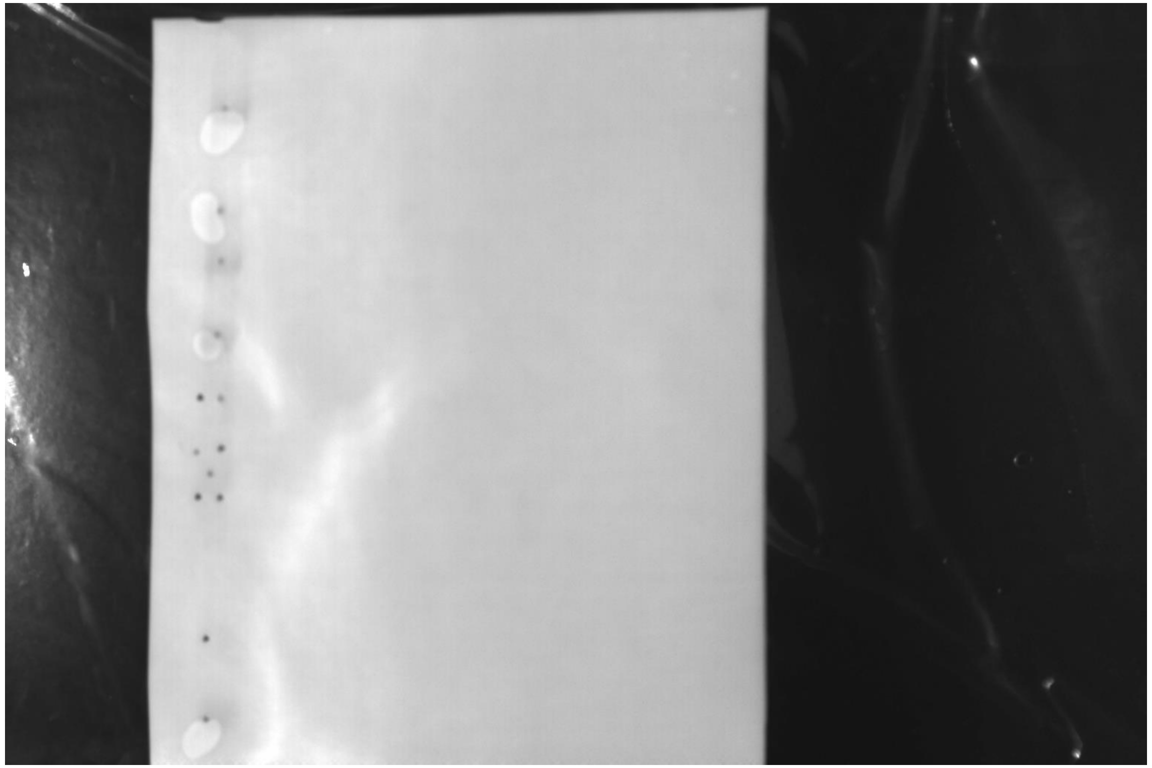

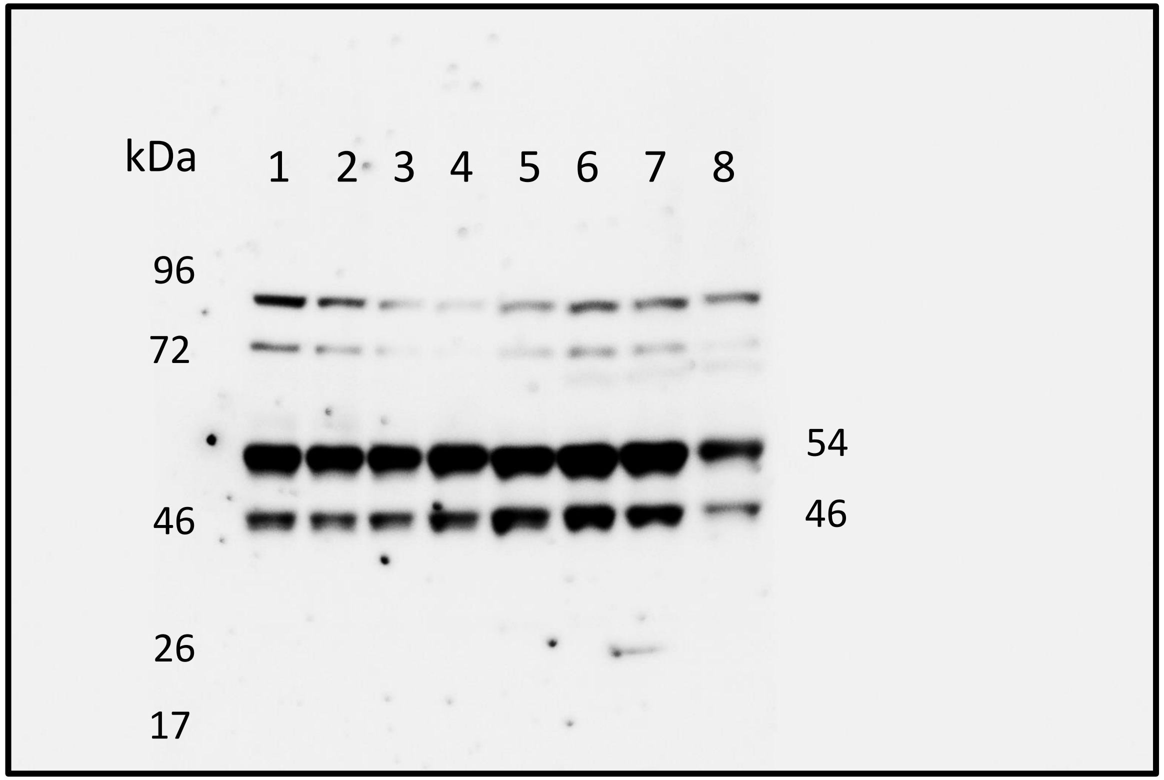

54kDa:100:122:104:125:168:183:227:62

46kDa:92:67:73:111:196:195:199:39

HT1080 24h treatment p38

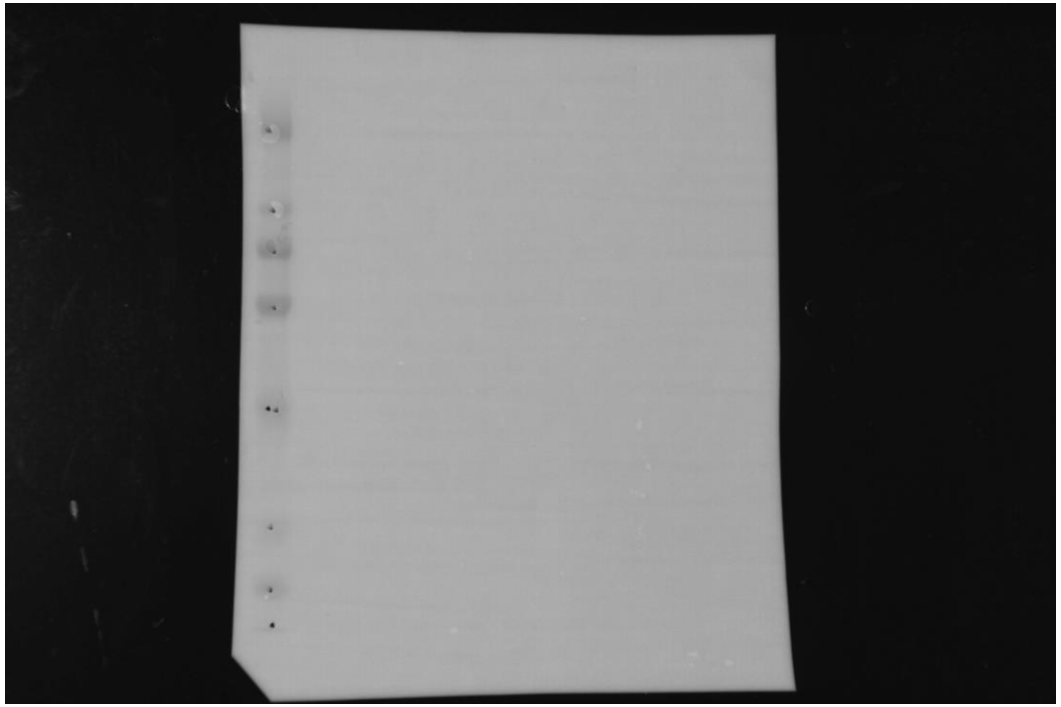

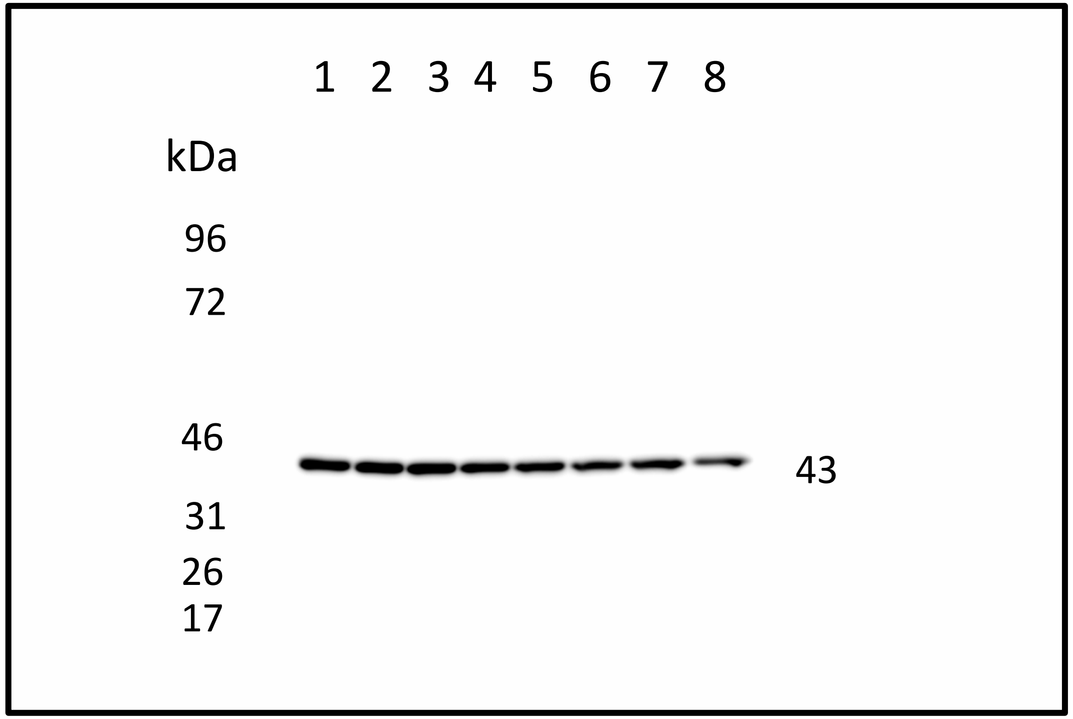

43kDa:100:112:124:98:89:68:85:46

HT1080 24h treatment MYC

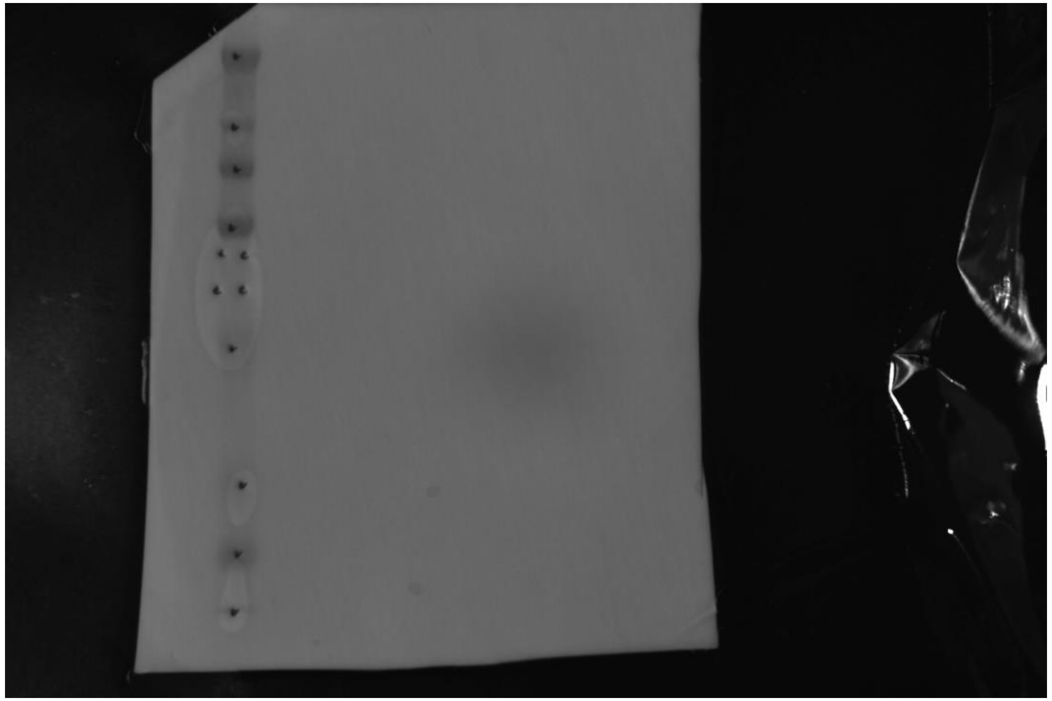

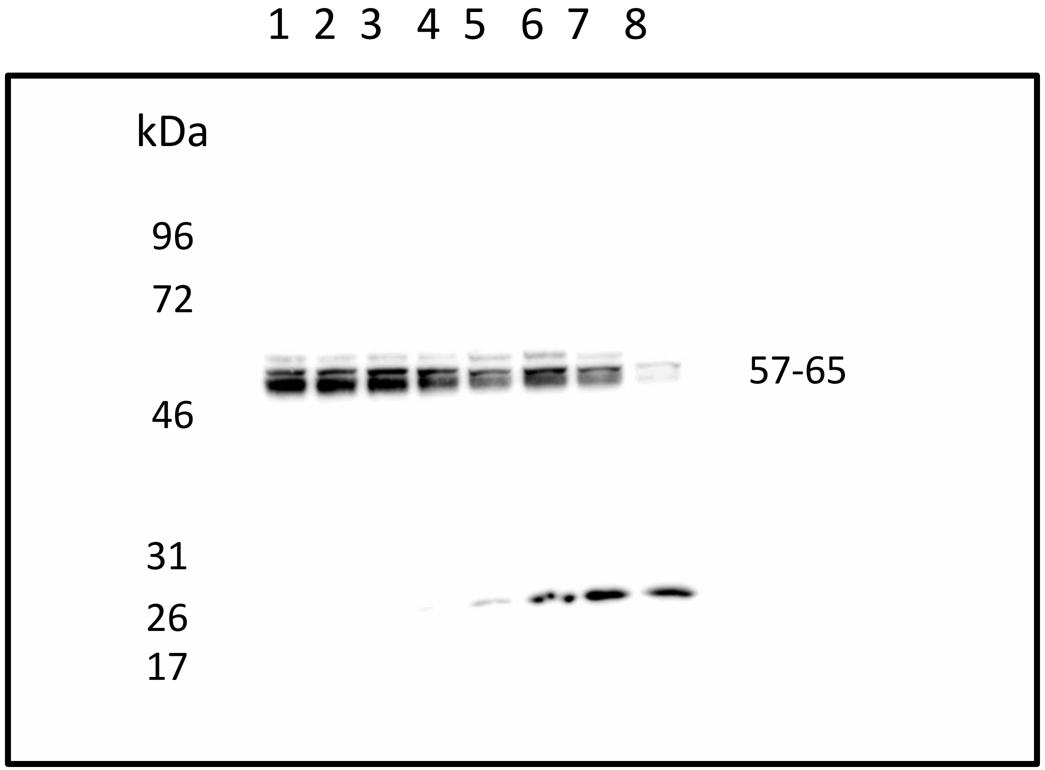

57-65kDa:100:105:121:62:37:63:34:3

HT1080 24h treatment Survivin

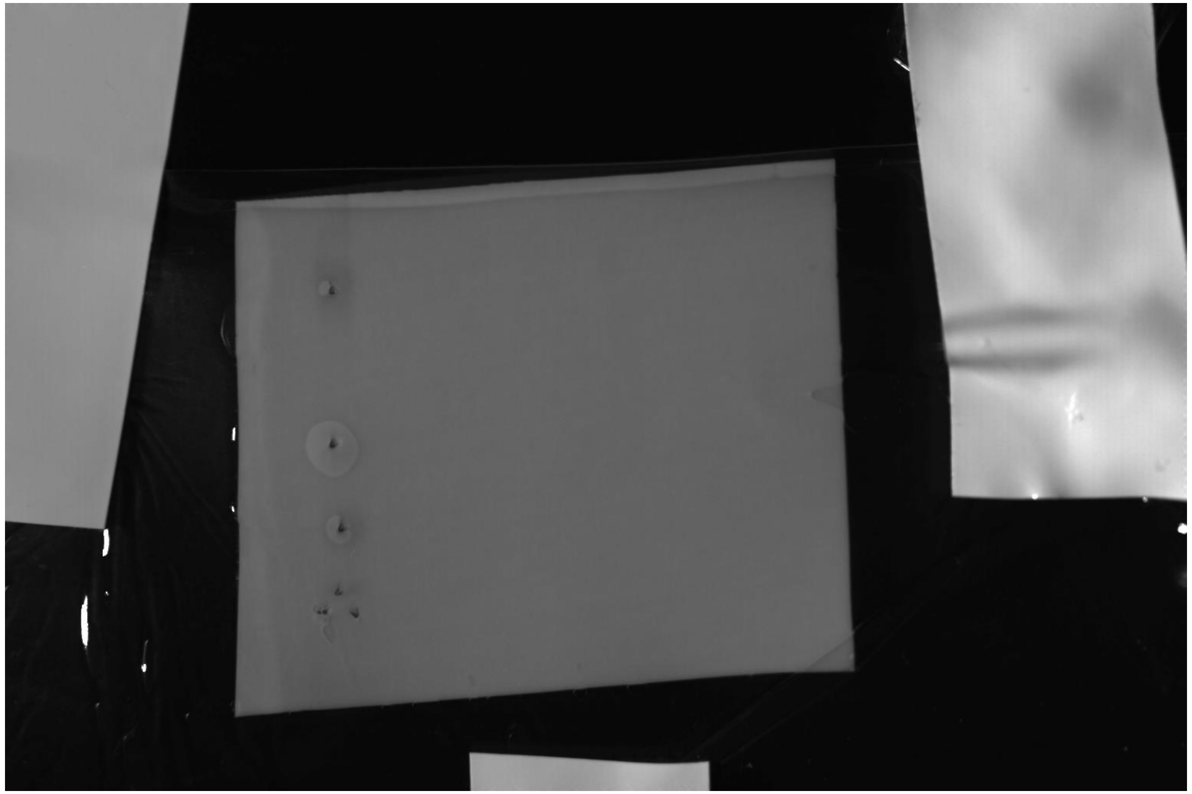

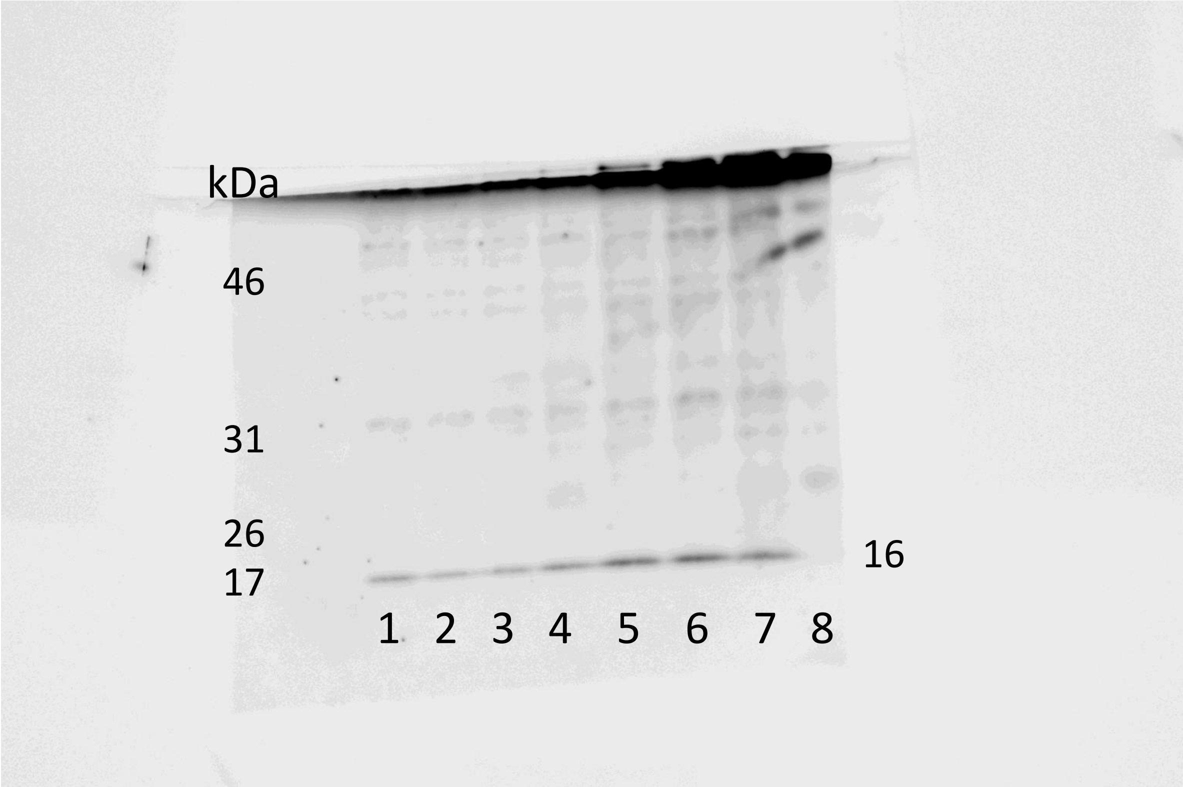

16kDa:100:65:81:151:268:307:188:29

HT1080 24h treatment MCL-1

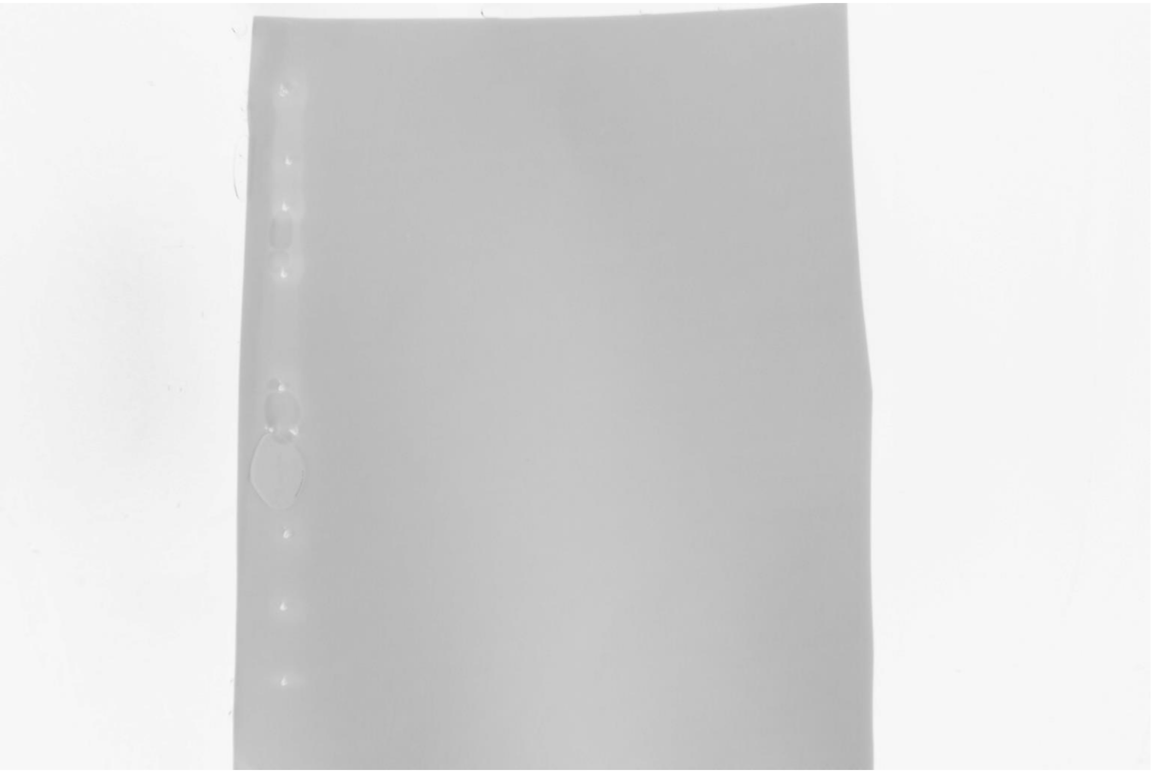

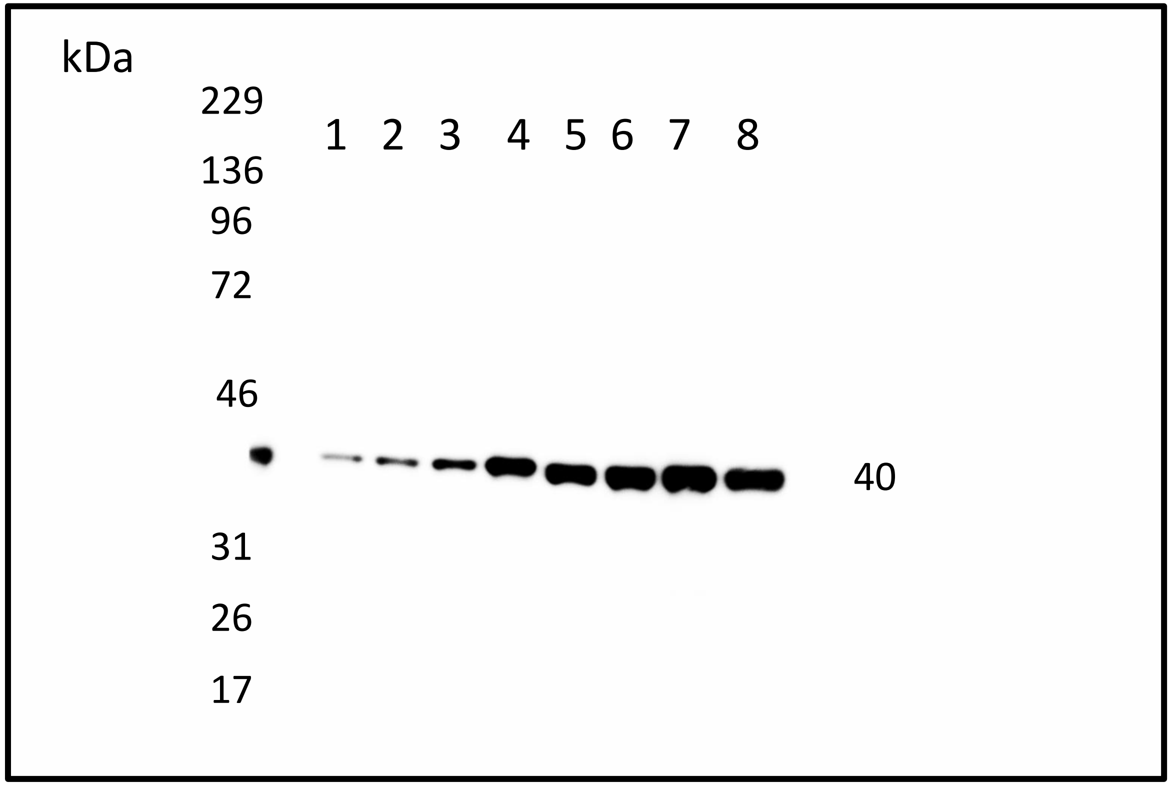

40kDa:2:8:27:87:88:104:125:100

HT1080 24 h treatment p-AKT

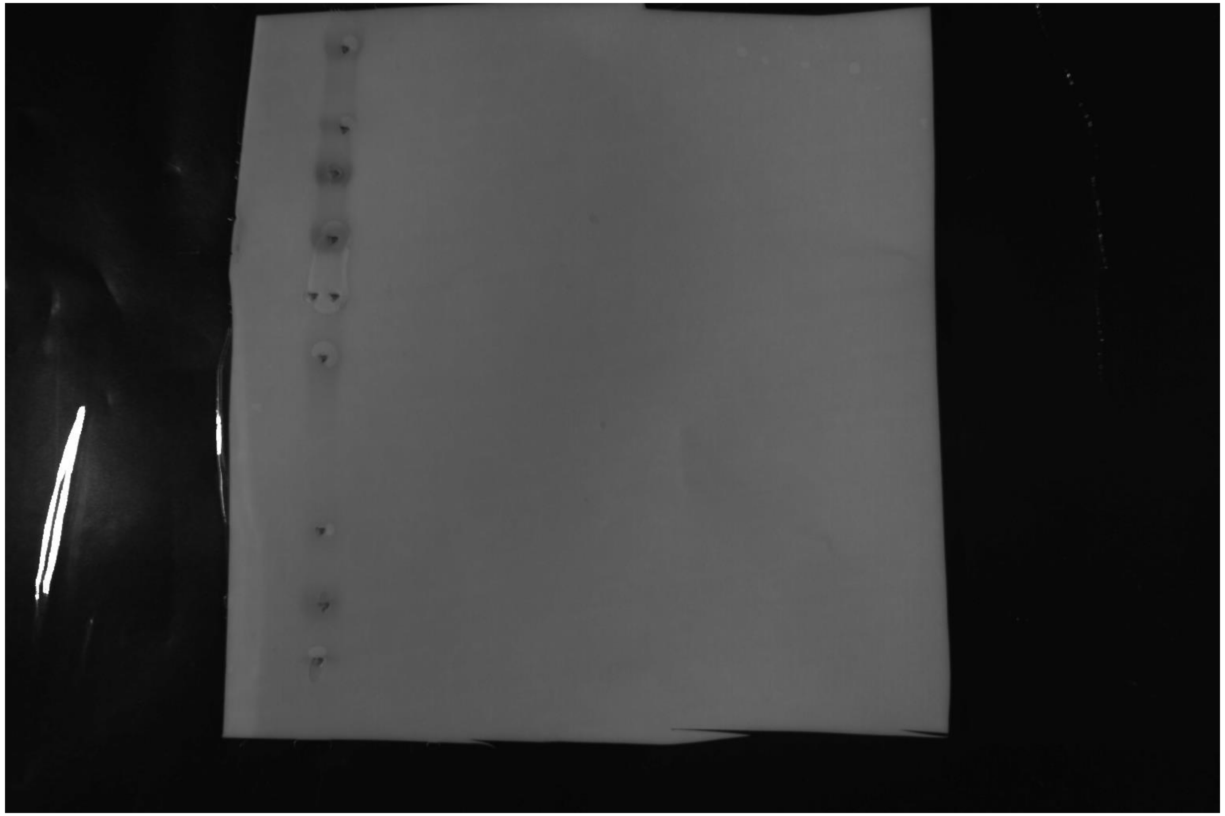

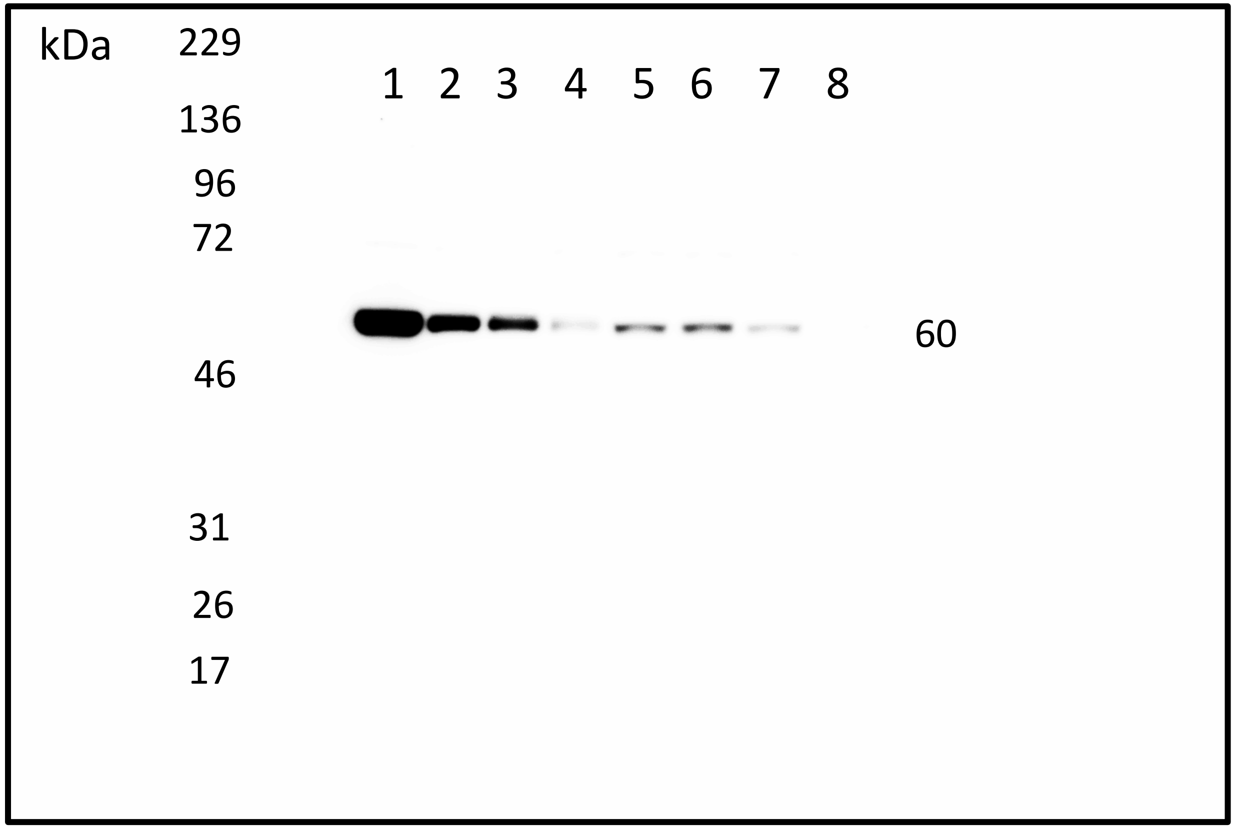

60kDa: 100:26:11:0:3:3:0:0

HT1080 24 h treatment AKT

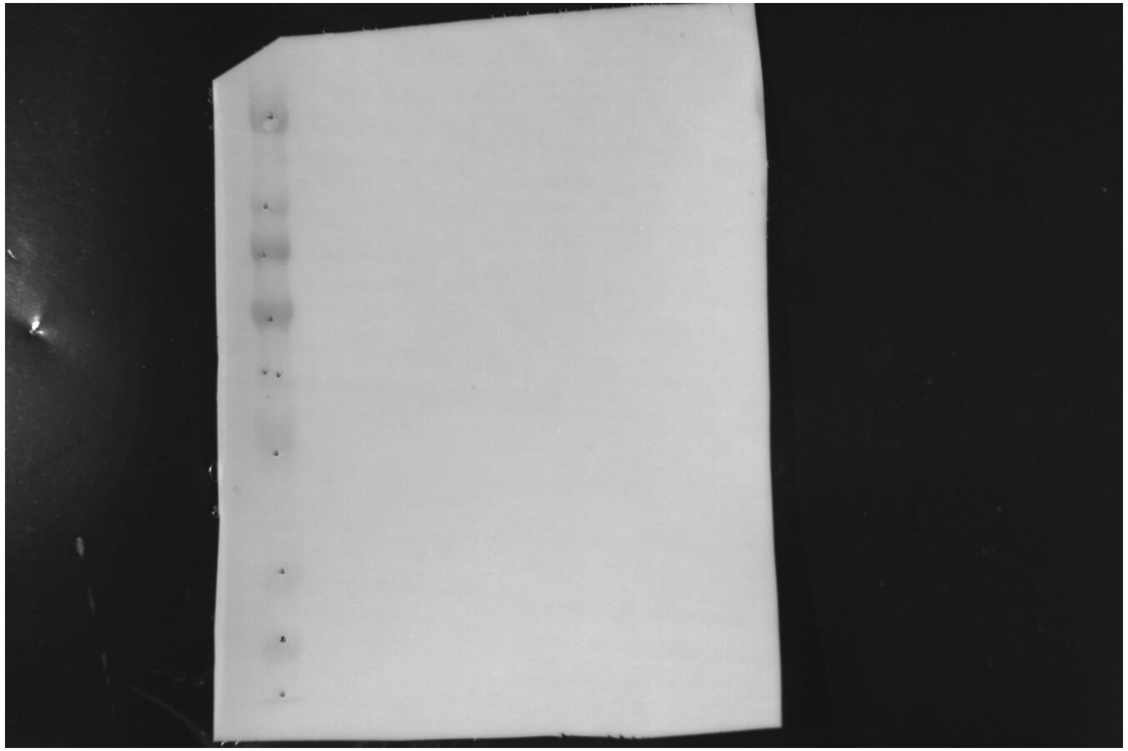

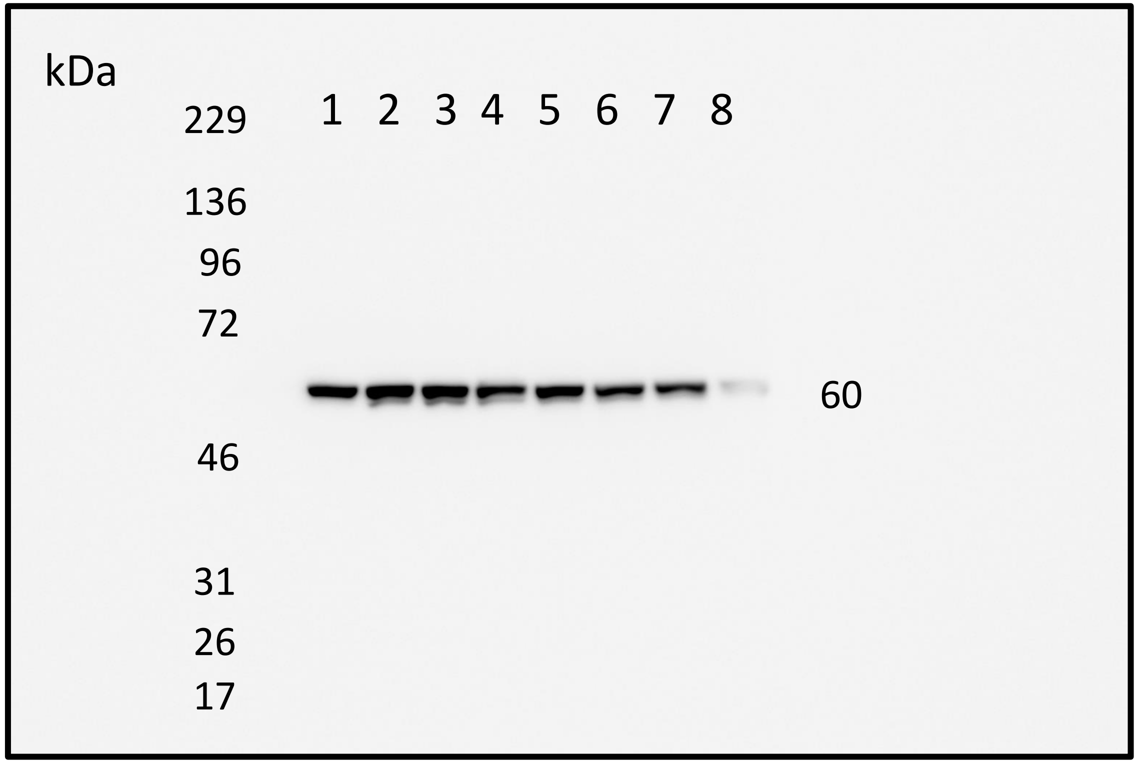

60kDa: 100:124:115:88:101:71:58:6

Lane No. LM8 treatment 6 h, 12 h

Treatment time 6 h

- 1. Everolimus 0 μM
- 2. Everolimus 5 μM
- 3. Everolimus 10 μM
- 4. Everolimus 0 μM Bortezomib 2.5nM
- 5. Everolimus 5 μM Bortezomib 2.5nM
- 6. Everolimus 10 μM Bortezomib 2.5nM

Treatment time 12 h

- 7. Everolimus 0 μM
- 8. Everolimus 5 μM
- 9. Everolimus 10 μM
- 10. Everolimus 0 μM Bortezomib 2.5nM
- 11. Everolimus 5 μM Bortezomib 2.5nM
- 12. Everolimus 10 μM Bortezomib 2.5nM

LM8 6 h 12 h treatment β actin

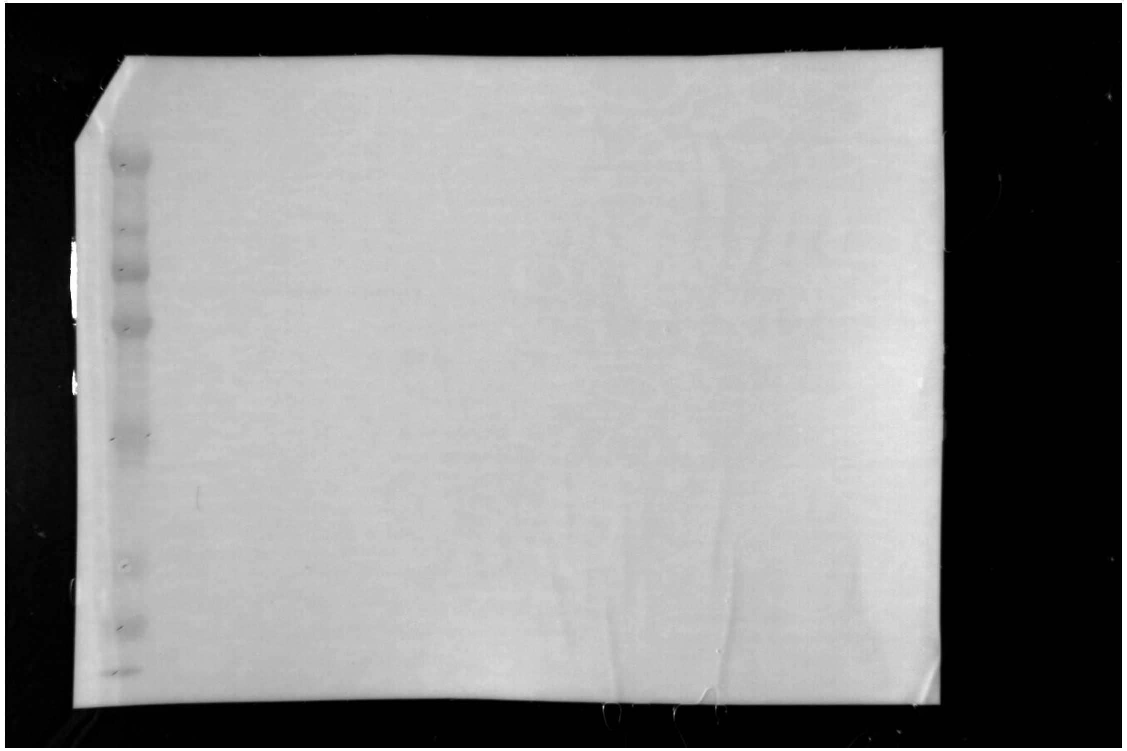

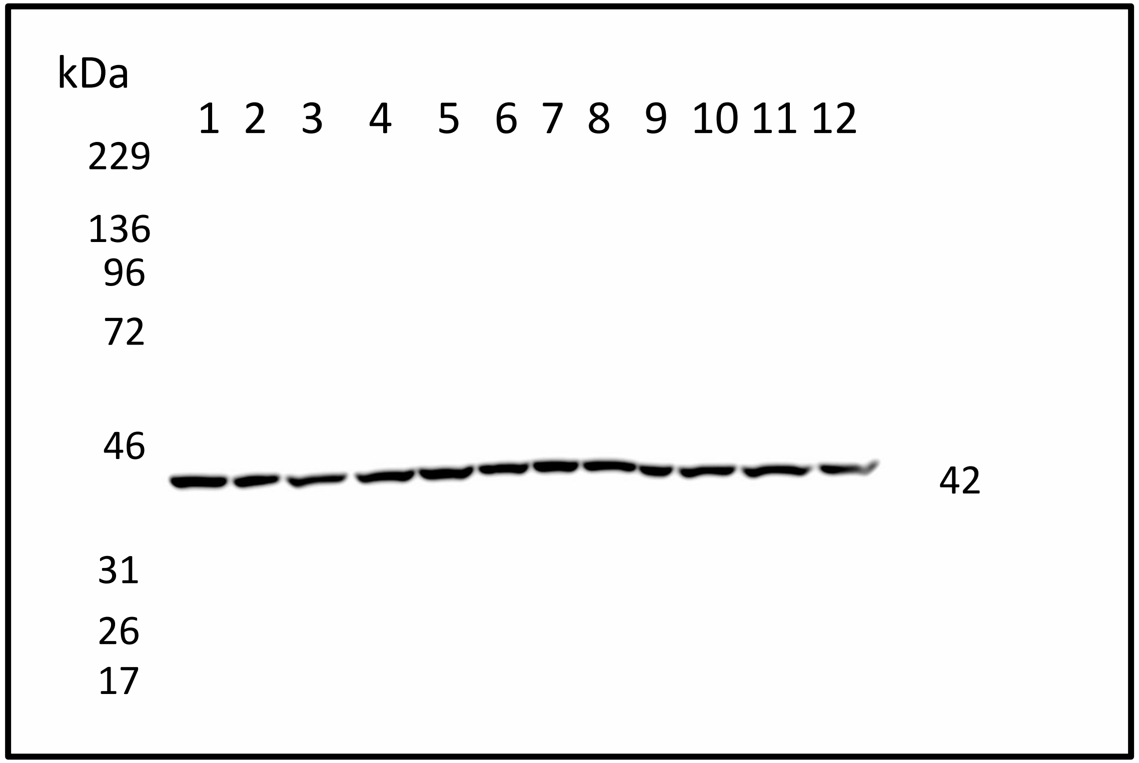

42kDa:100:44:45:71:69:58:55:70:39:63:60:31

LM8 6 h 12 h treatment cleaved caspase 3

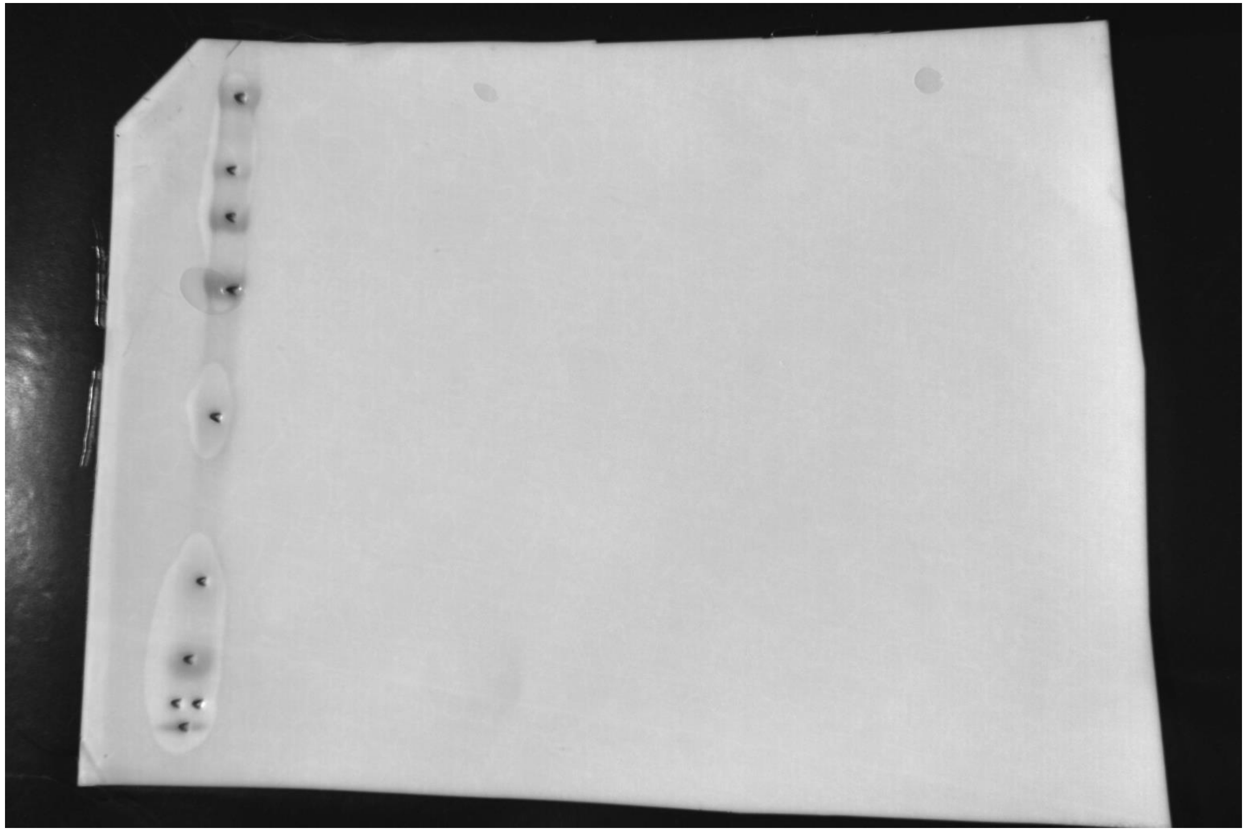

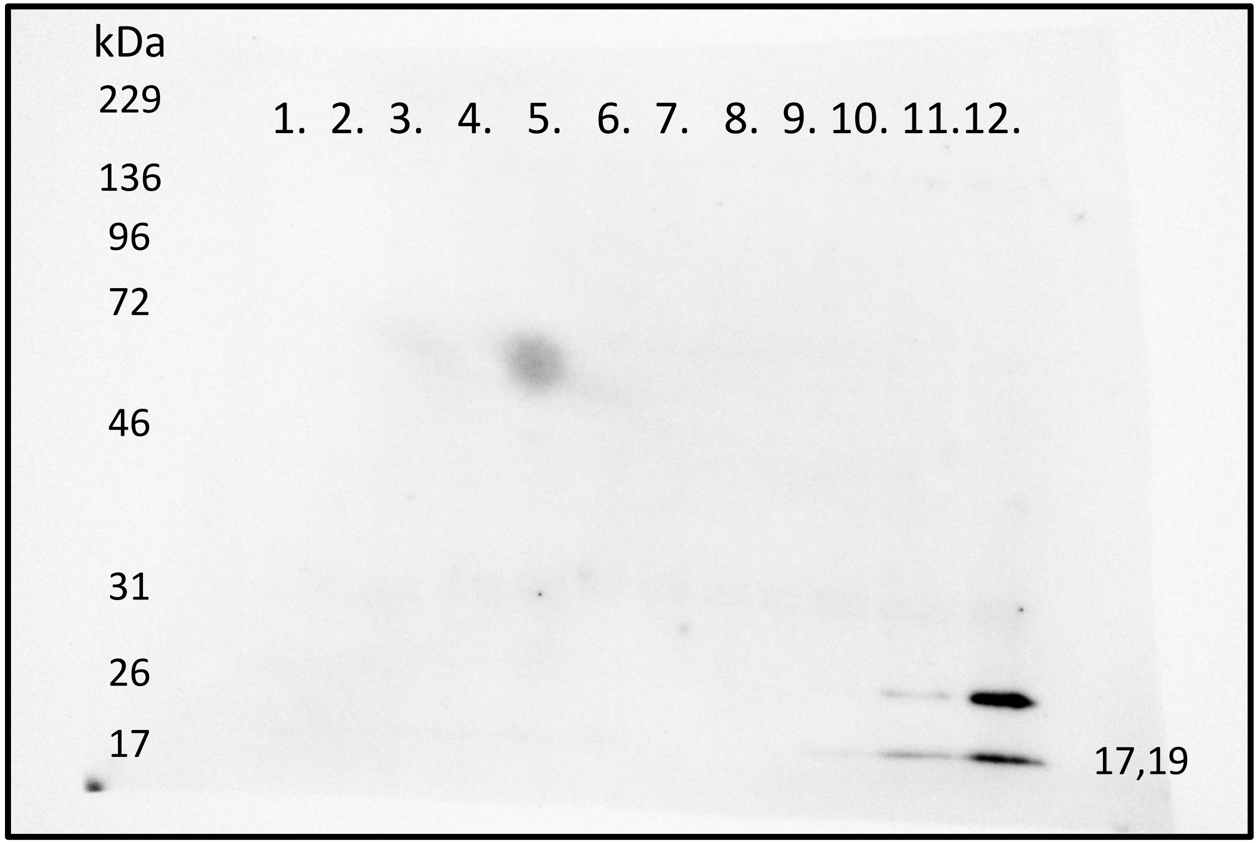

17,19kDa:0:0:0:0:0:0:0:0:0:4:16:100

LM8 6 h 12 h treatment cleaved PARP

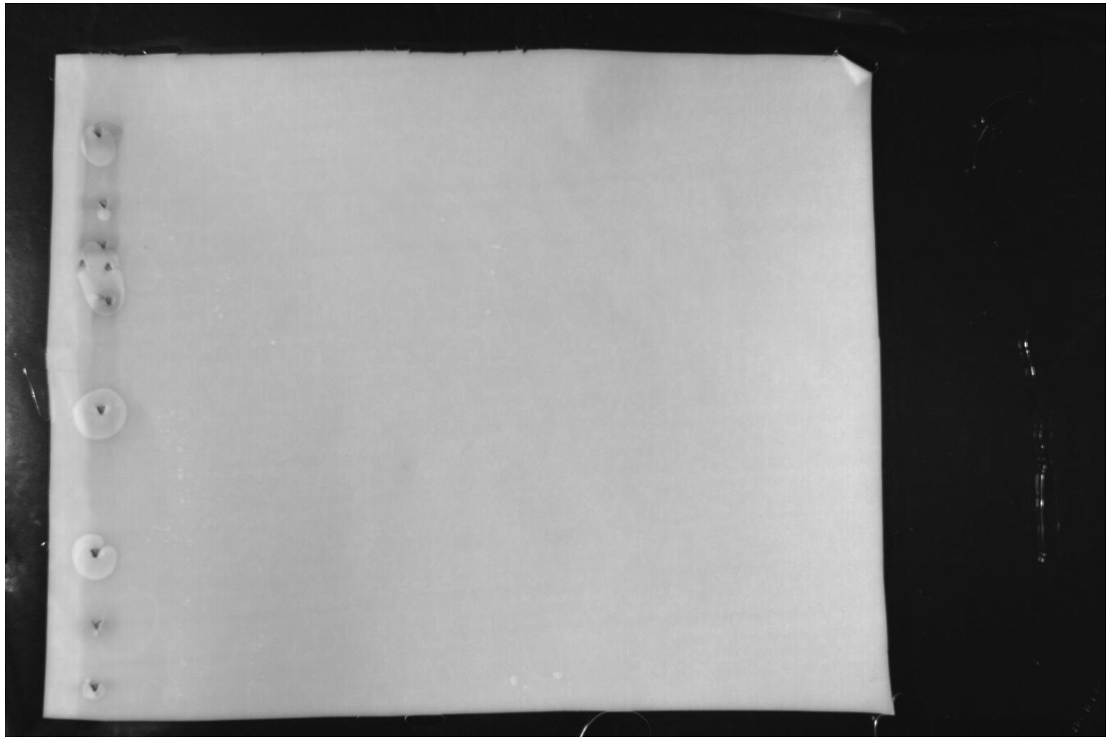

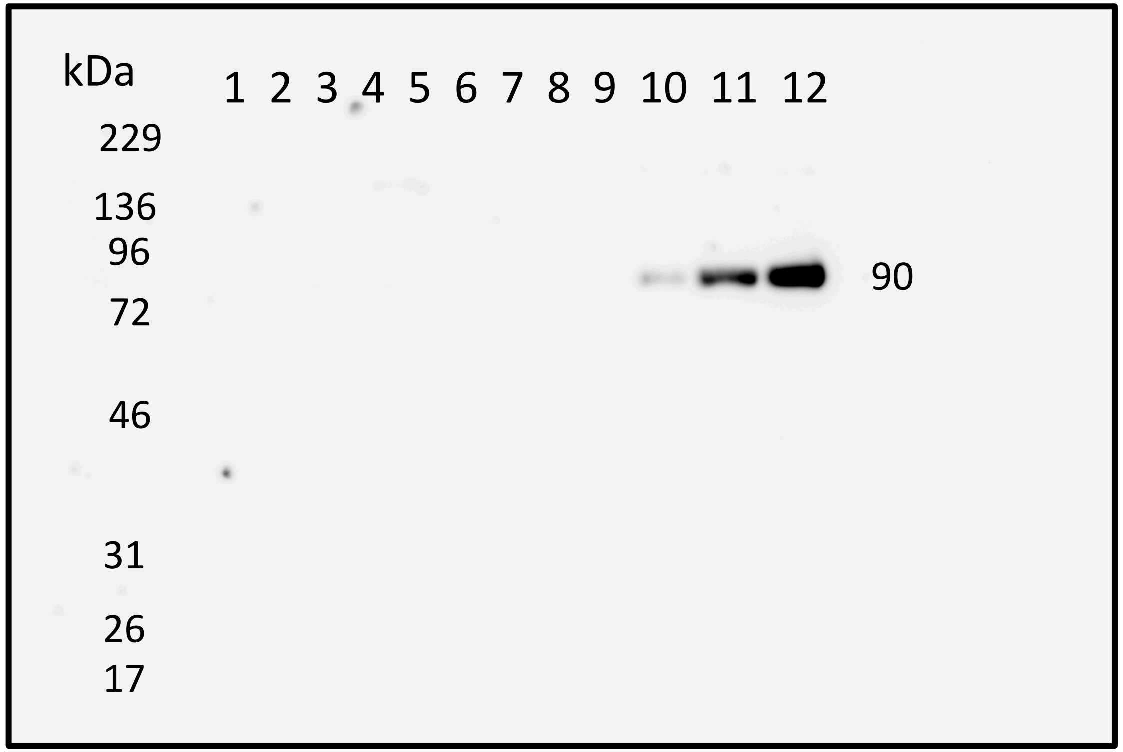

90kDa:0:0:0:0:0:0:0:0:0:2:23:100

LM8 6 h 12 h treatment p-ERK

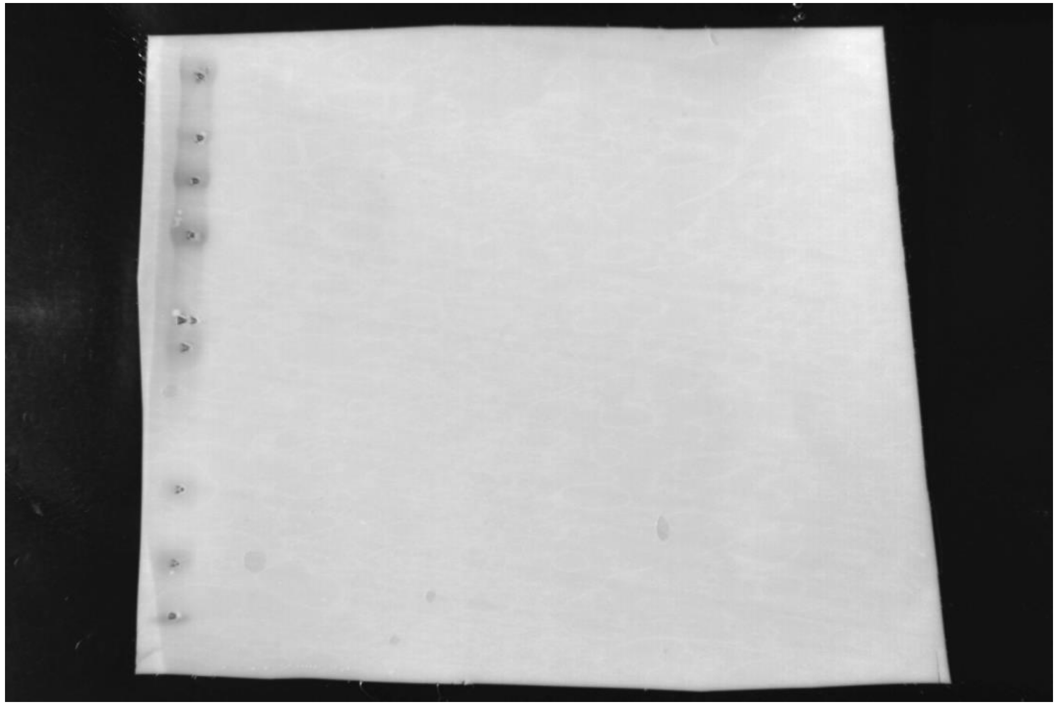

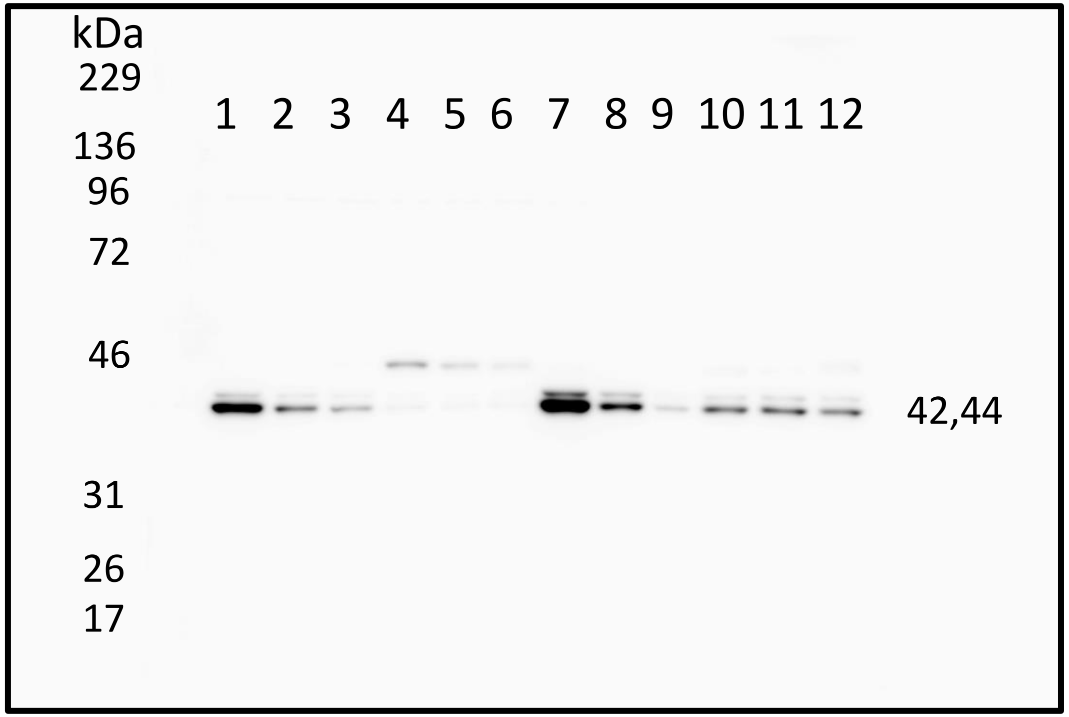

42,44kDa:100:16:7:1:0:1:147:42:4:19:20:15

LM8 6 h 12 h treatment ERK

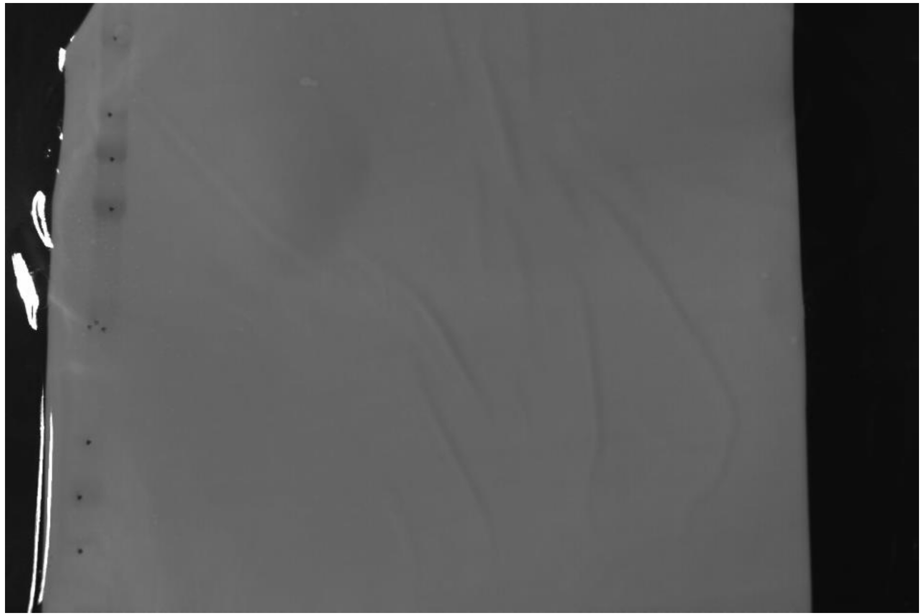

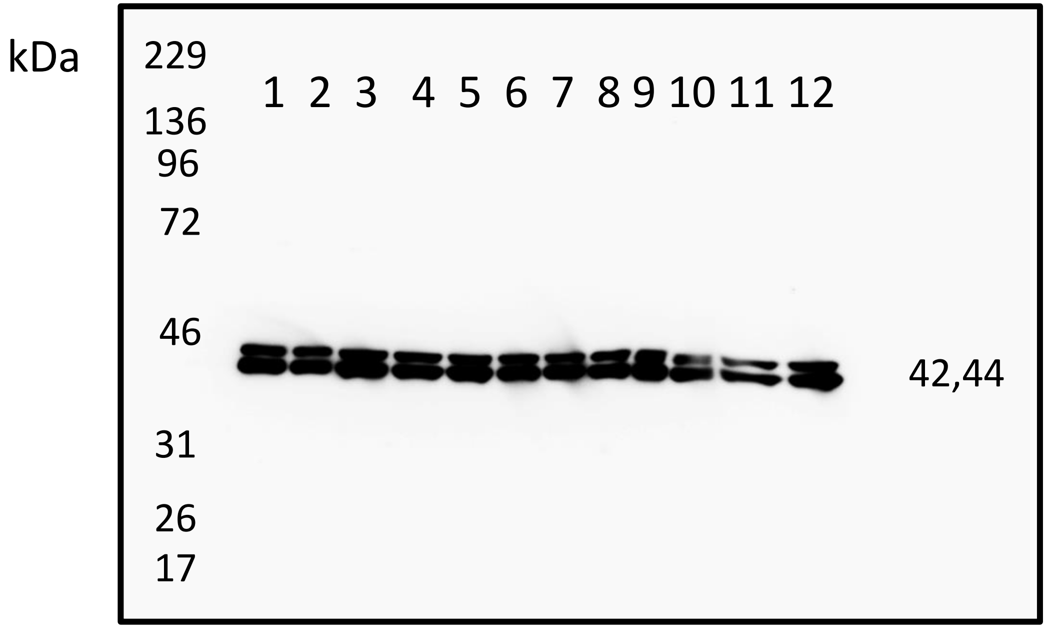

42,44kDa:100:85:120:88:99:92:90:82:67:51:32:62

LM8 6 h 12 h treatment p-JNK

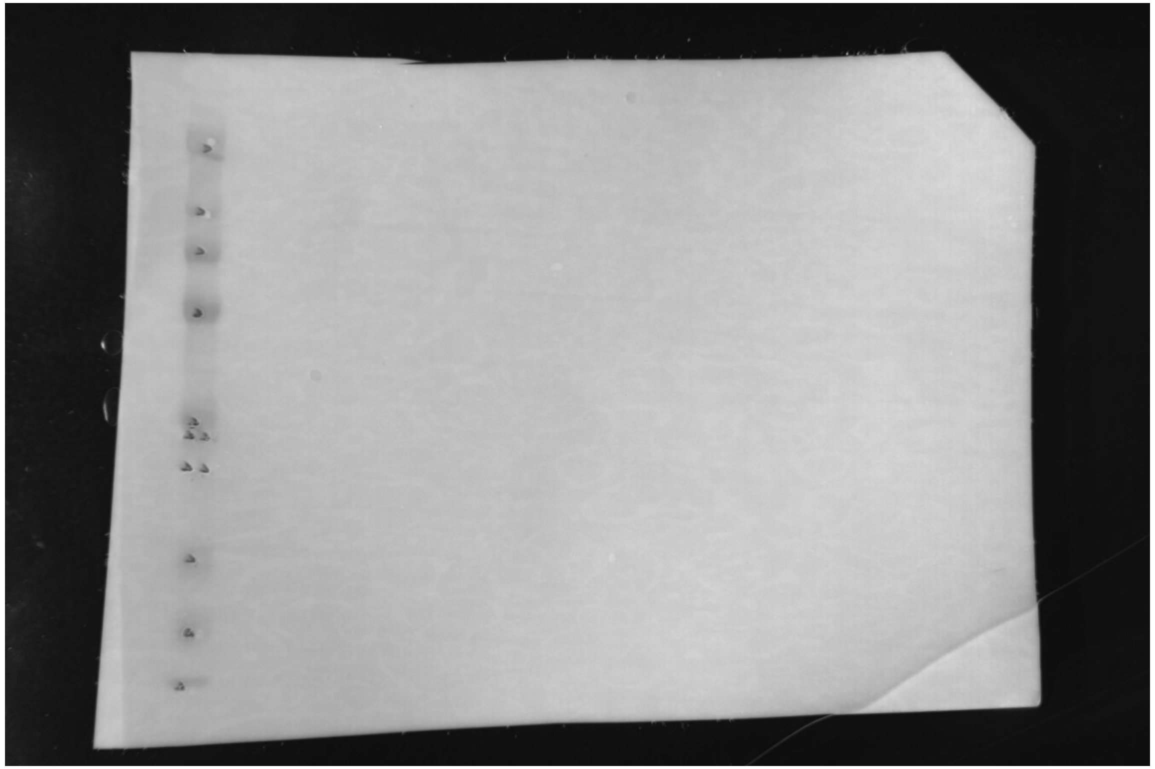

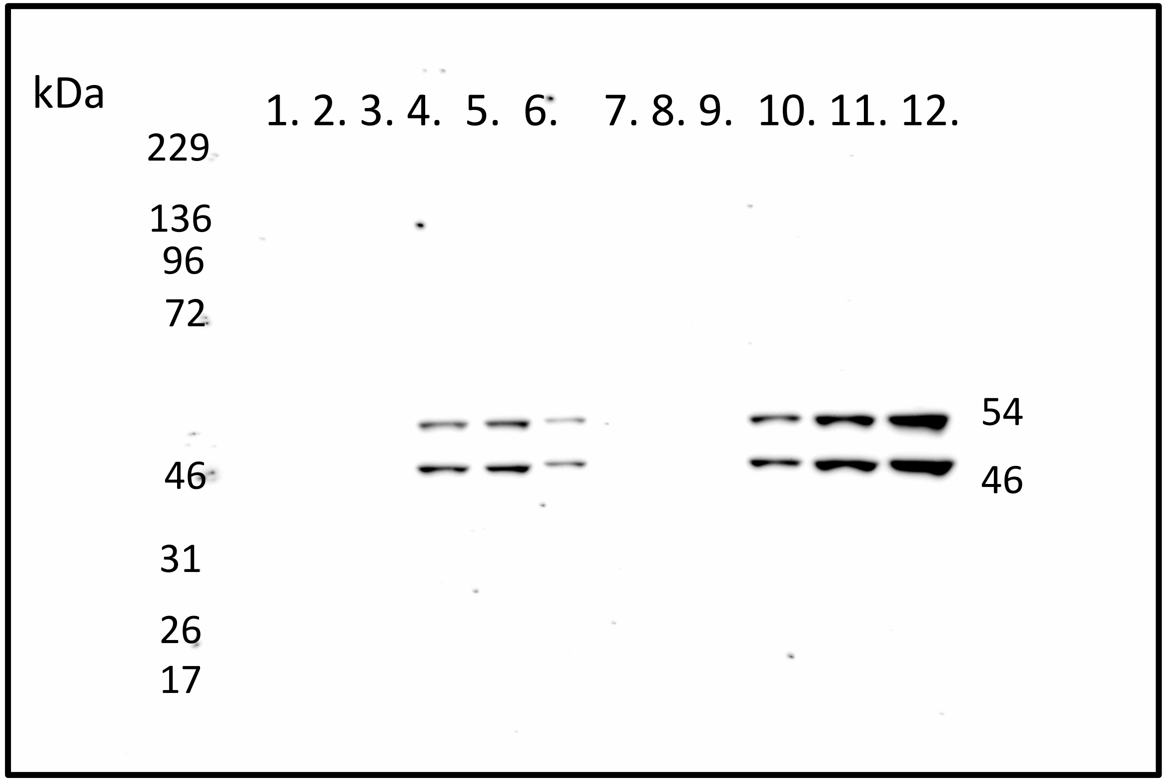

54kDa:0:0:0:13:17:3:0:0:0:22:70:100

46kDa:0:0:0:25:29:7:0:0:0:32:94:148

LM8 6 h 12 h treatment JNK

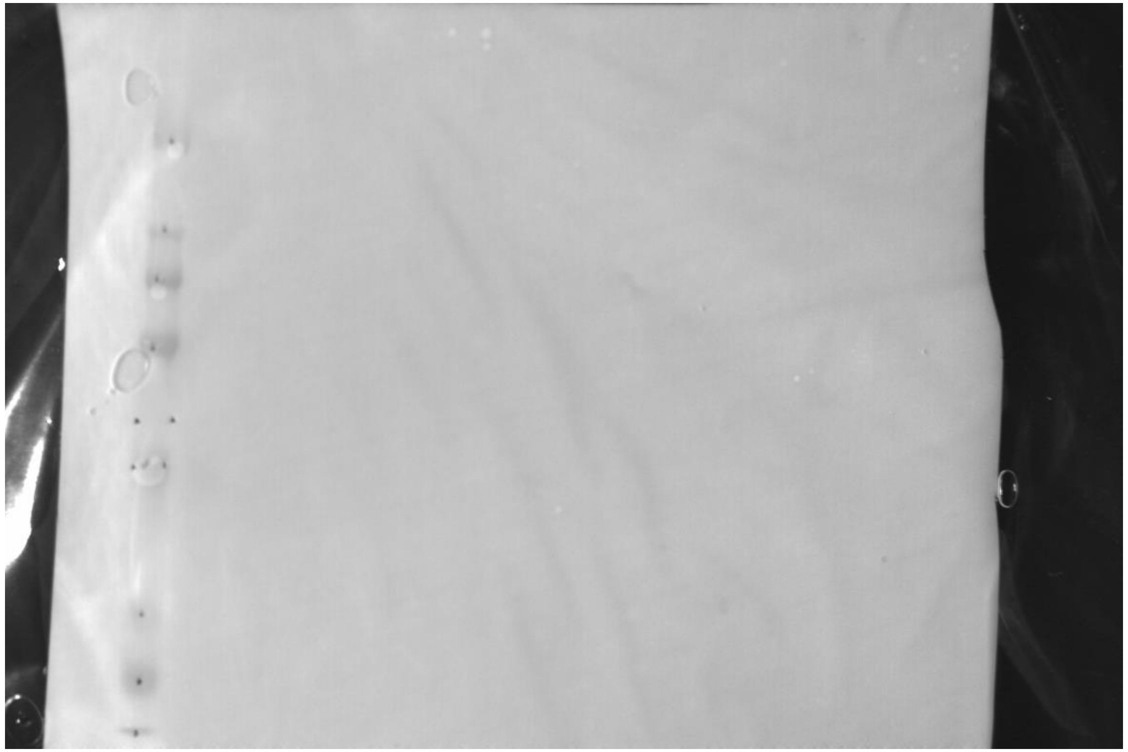

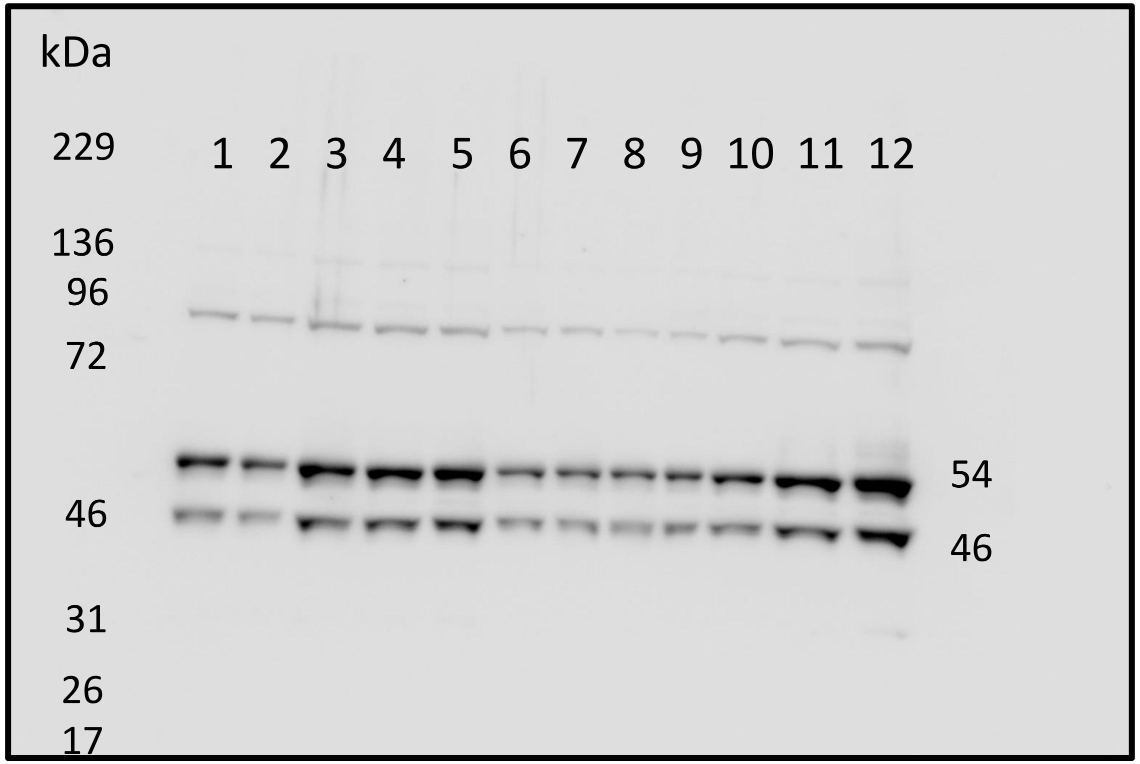

54kDa:27:14:43:40:44:12:12:12:12:21:46:100

46kDa:9:5:16:14:20:6:5:5:6:9:14:37

LM8 6 h 12 h treatment p-p38

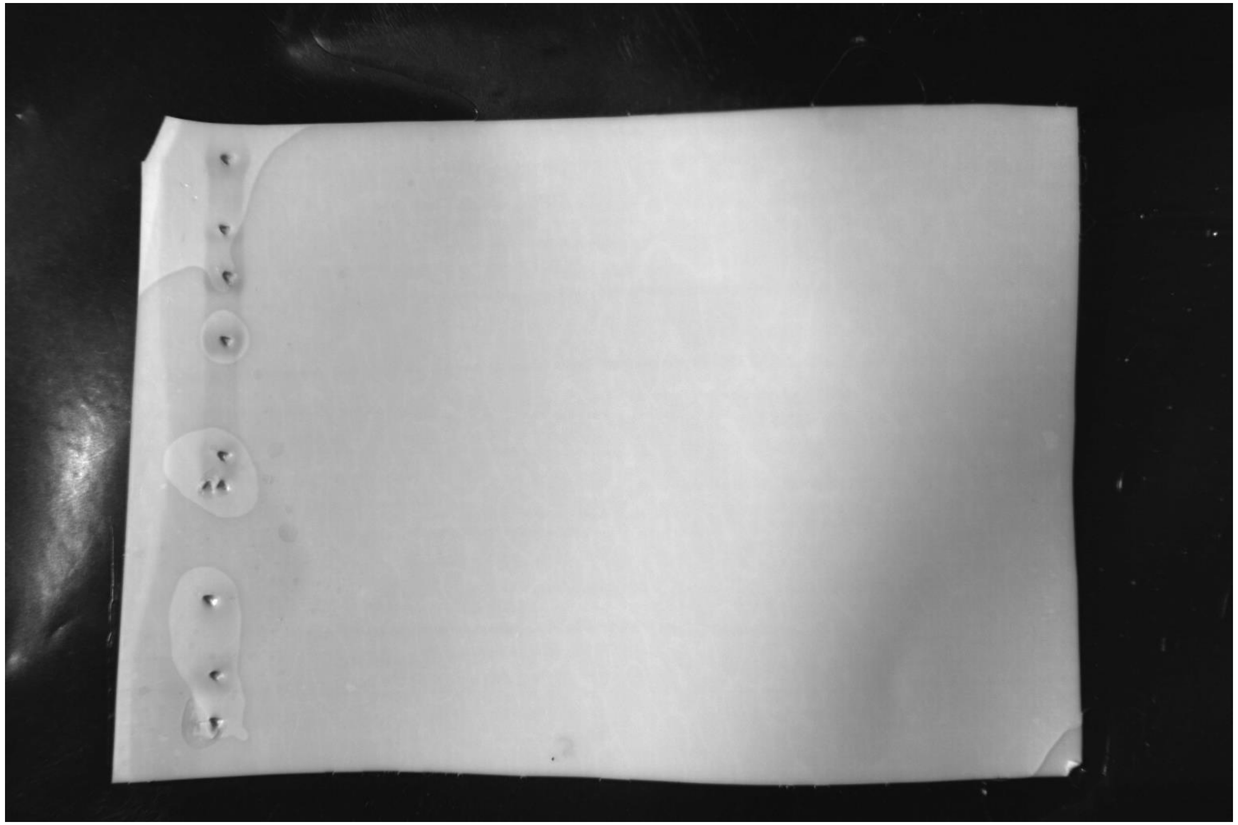

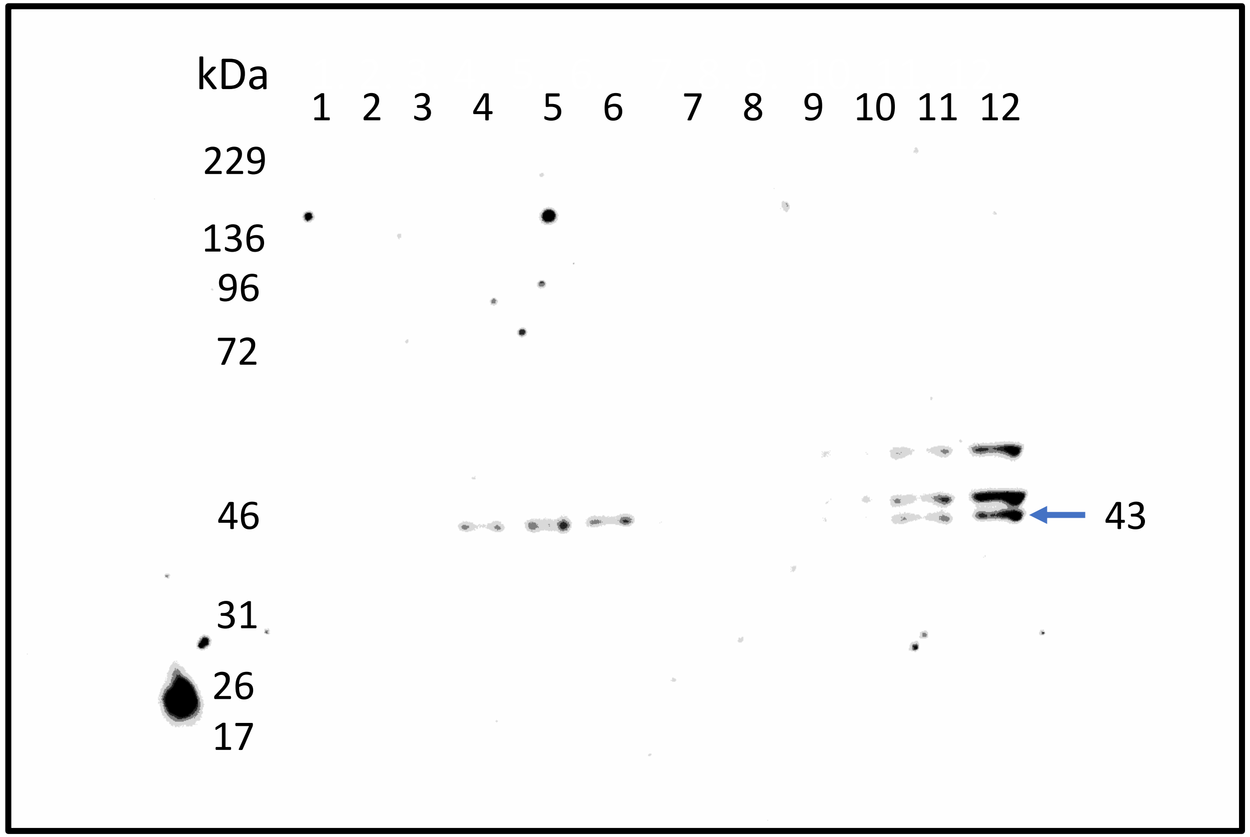

43kDa:0:0:0:13:36:25:0:0:0:0:19:100

LM8 6 h 12 h treatment p38

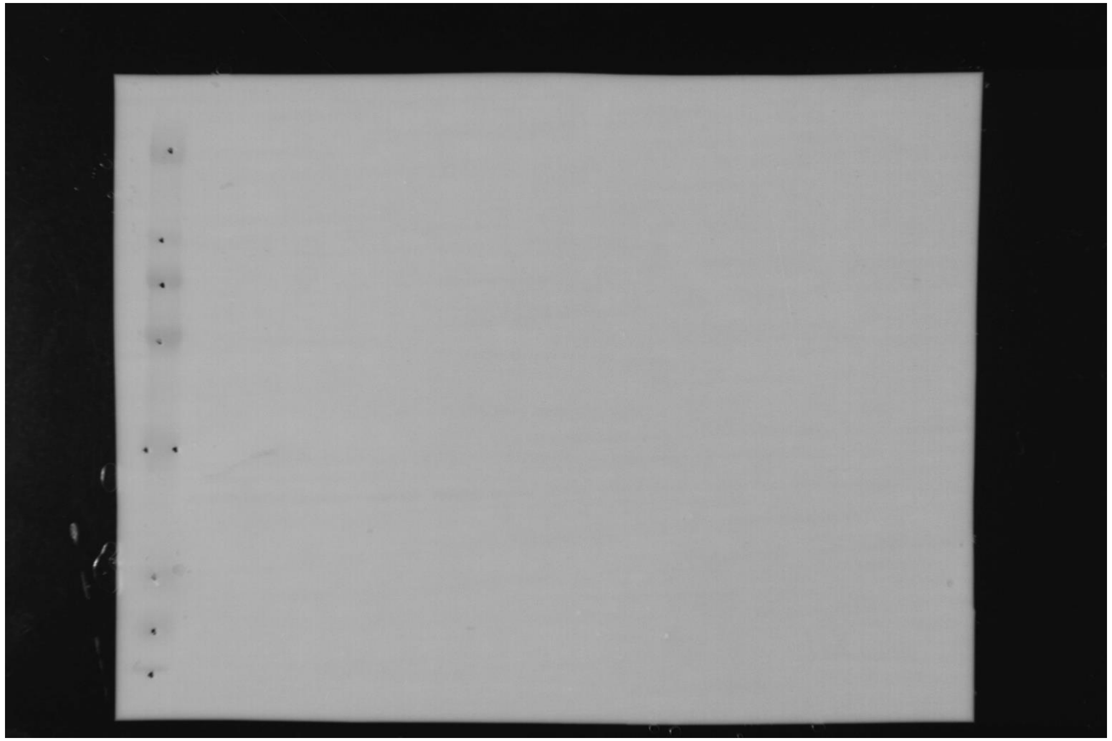

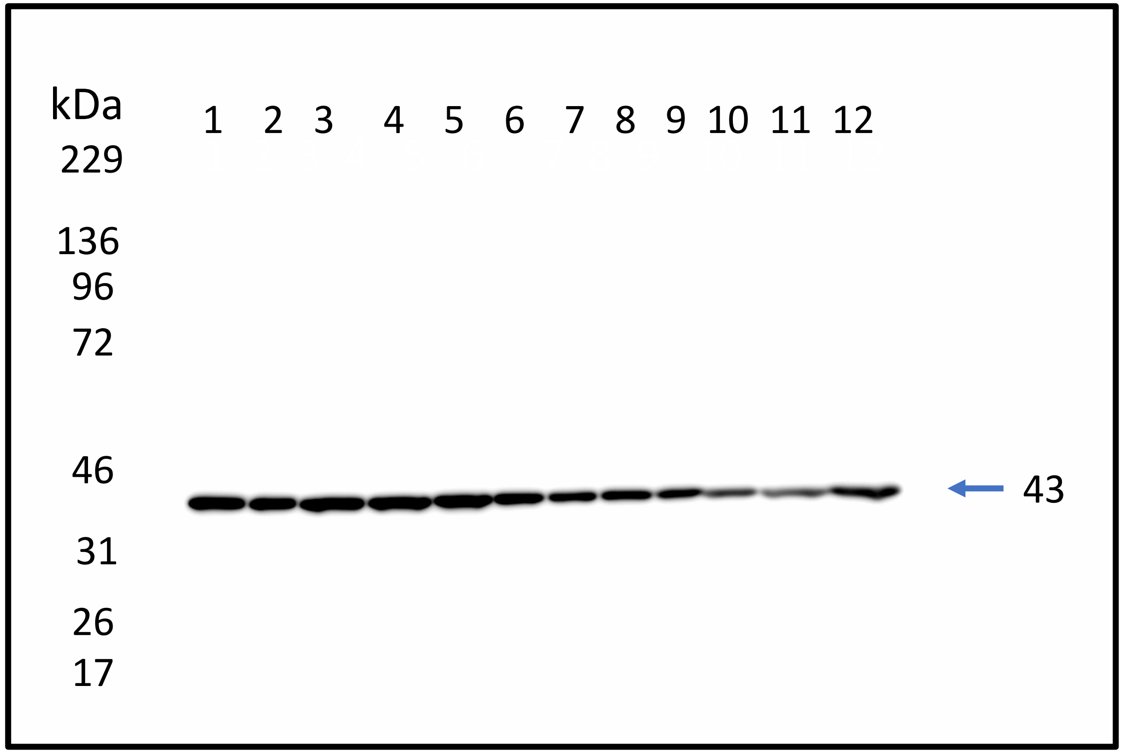

43kDa:100:55:106:112:104:60:39:47:29:17:14:41

LM8 6 h 12 h treatment MYC

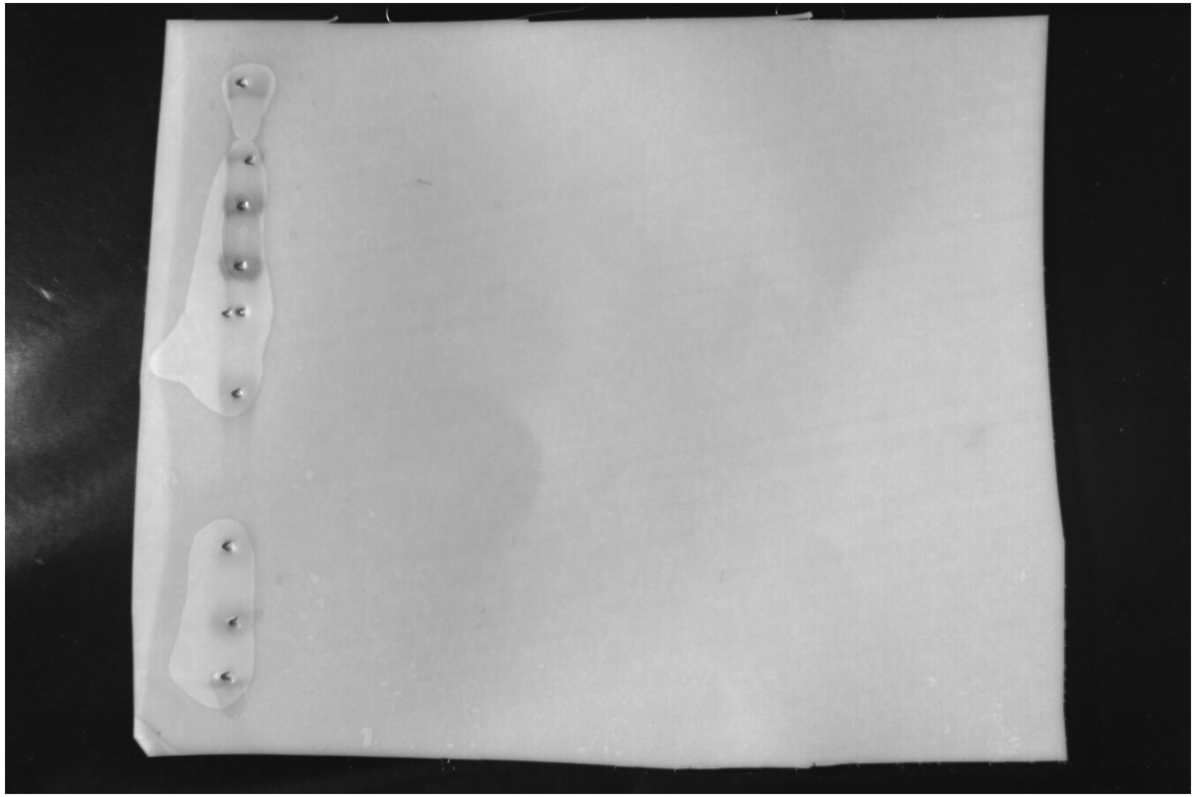

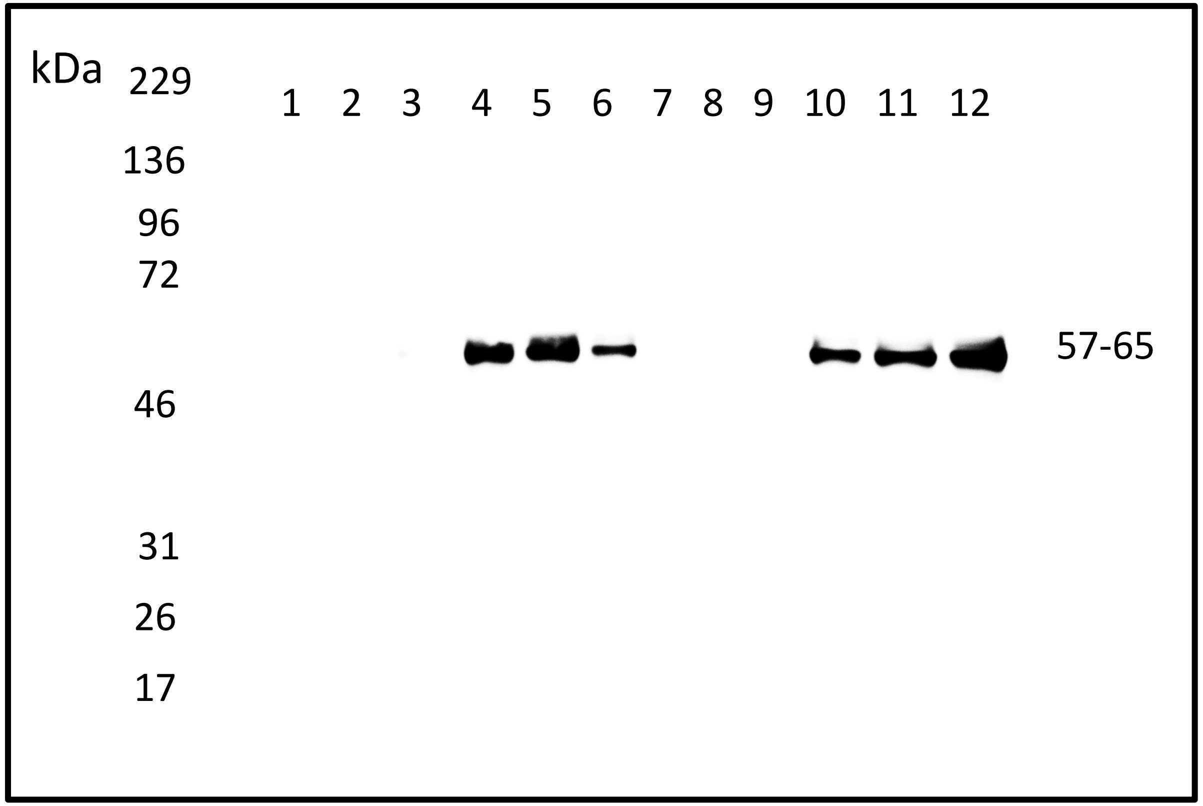

57-65kDa:0:0:0:47:82:17:0:0:0:34:62:100

LM8 6 h 12 h treatment Survivin

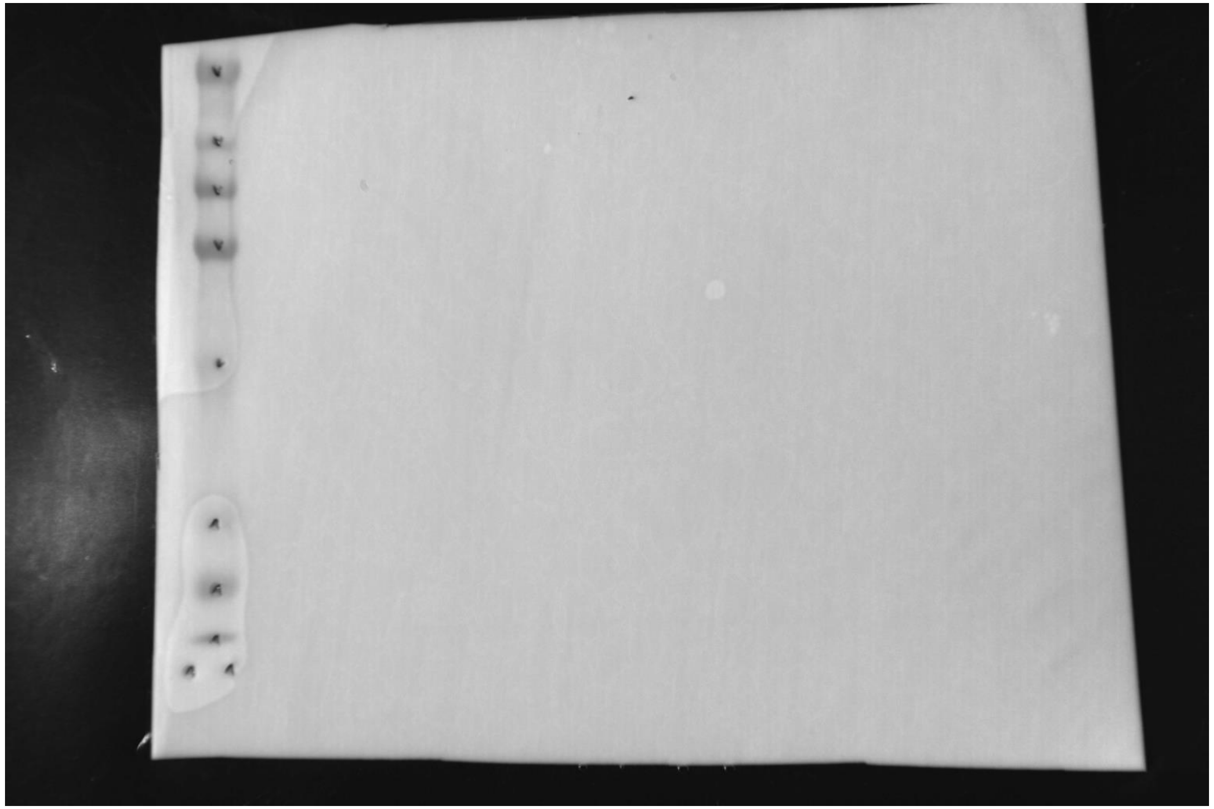

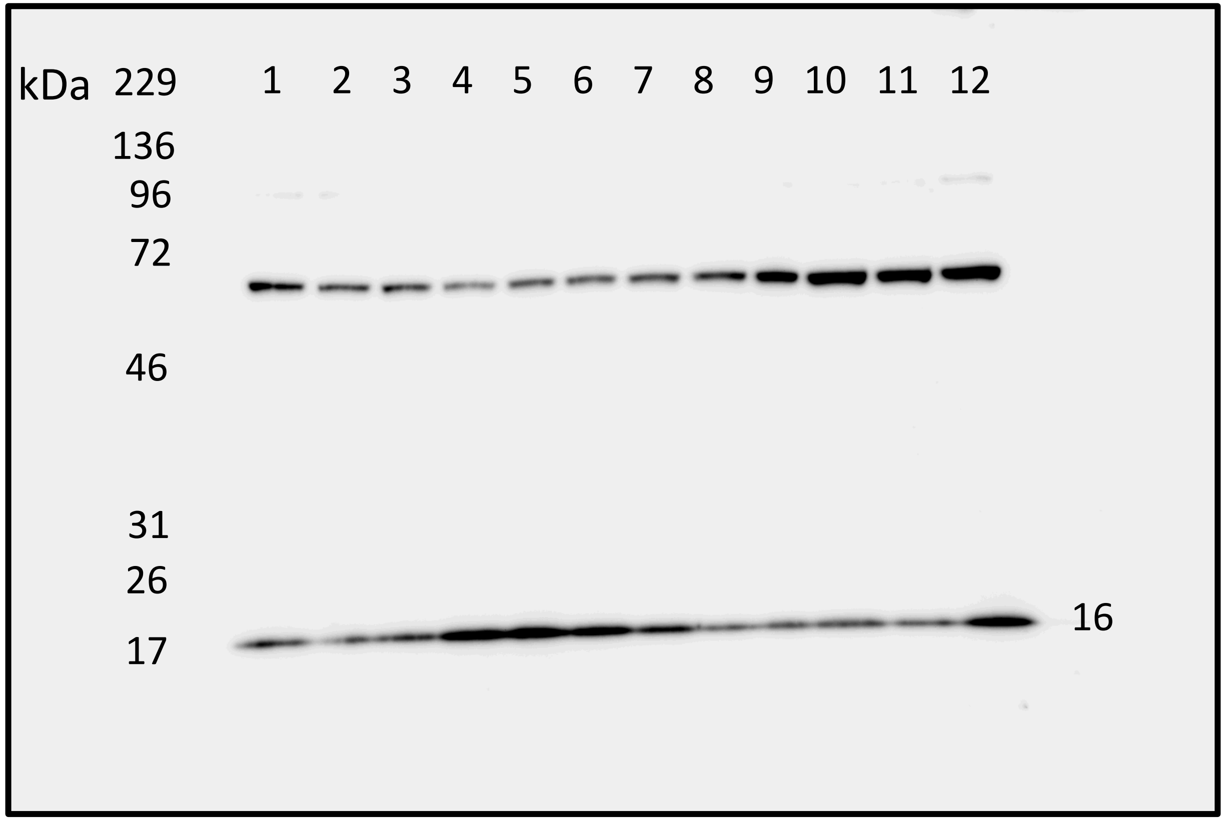

16kDa:100:55:102:228:571:422:209:101:73:89:71:215

LM8 6 h 12 h treatment p-AKT

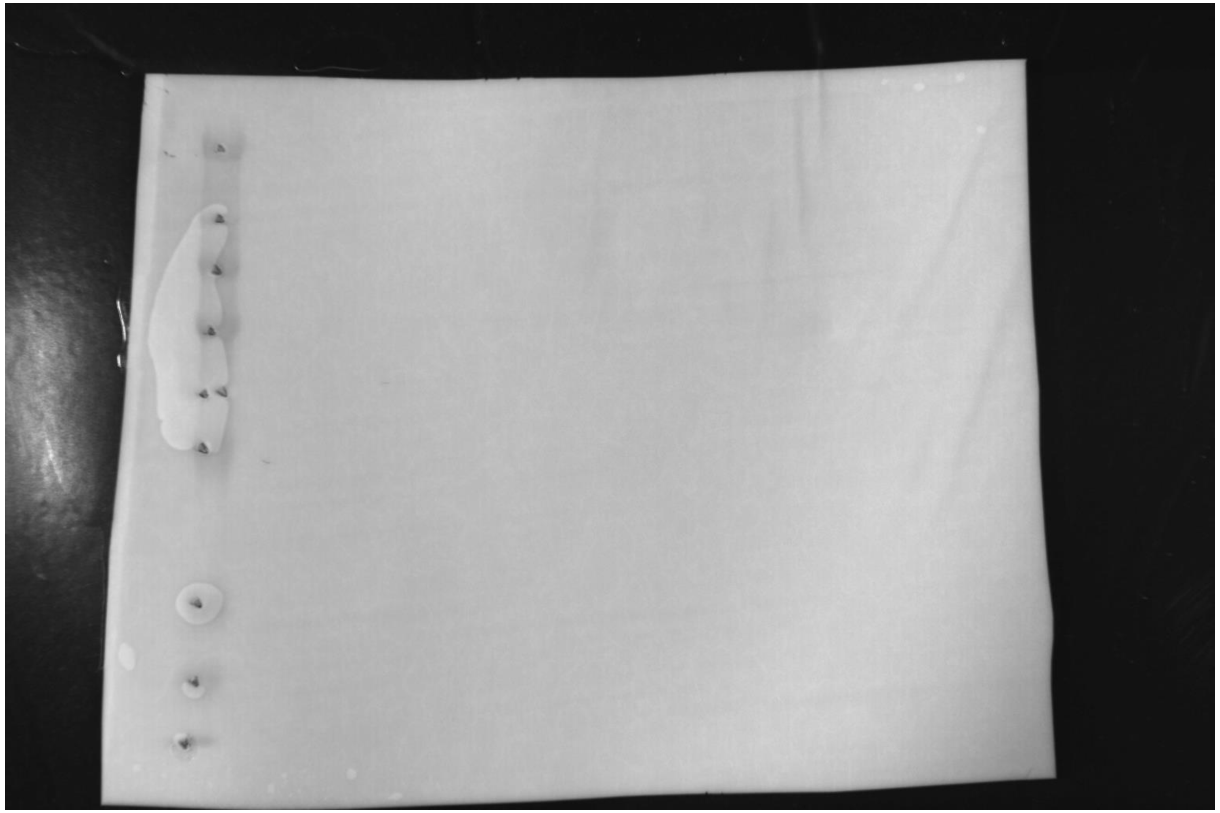

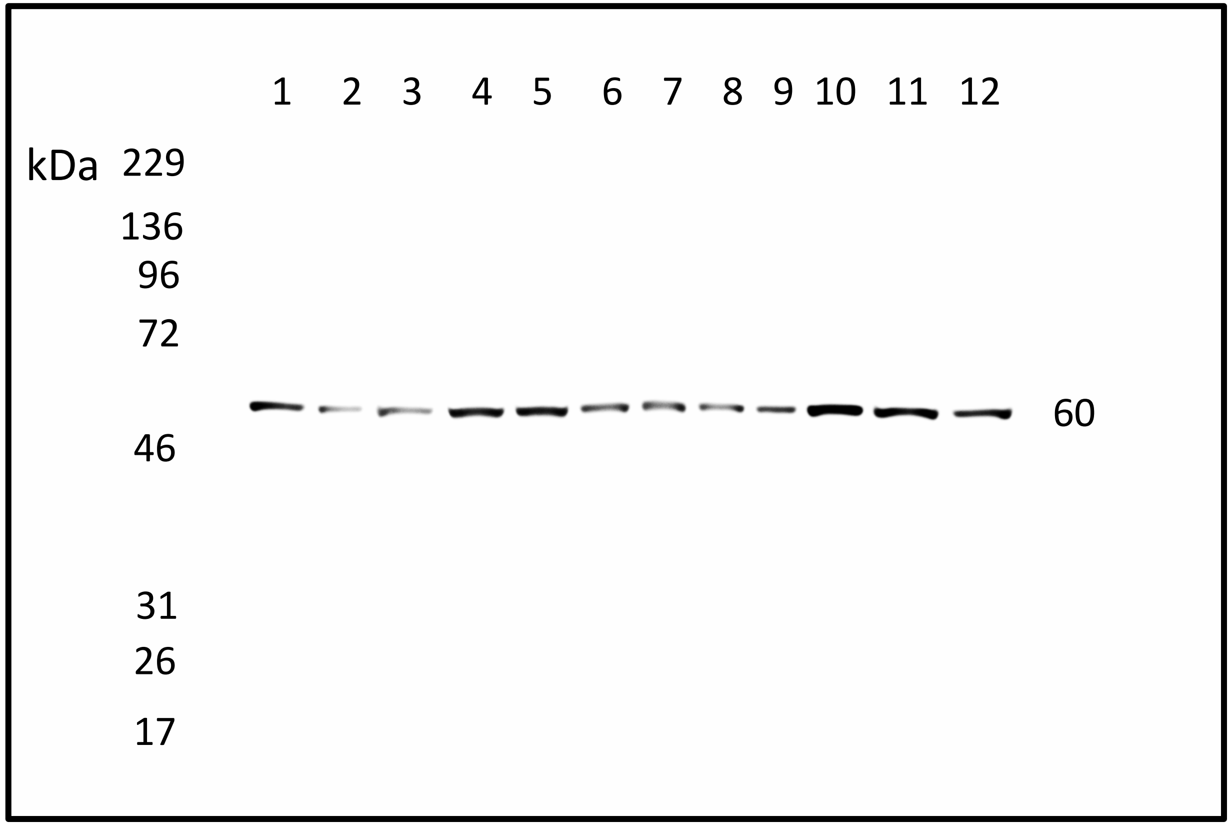

60kDa:100:17:32:129:128:50:45:33:38:236:191:117

LM8 6 h 12 h treatment AKT

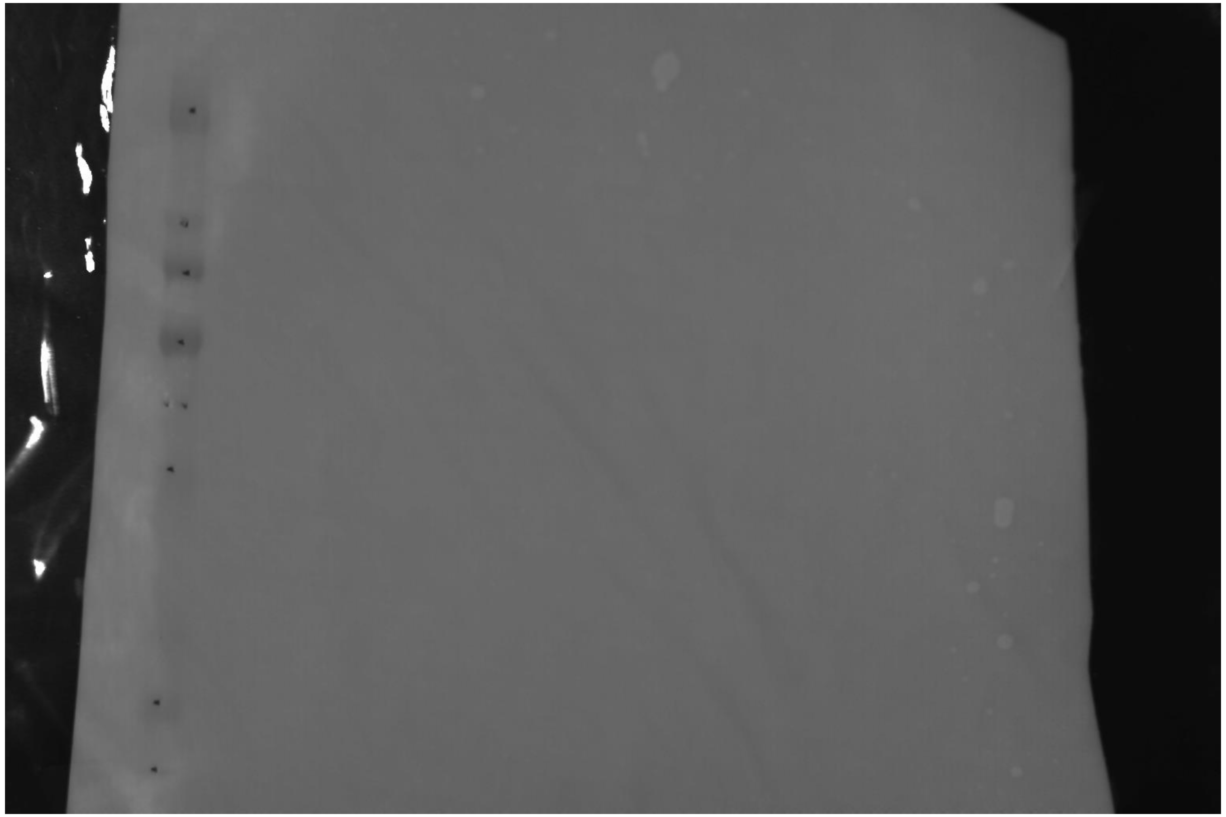

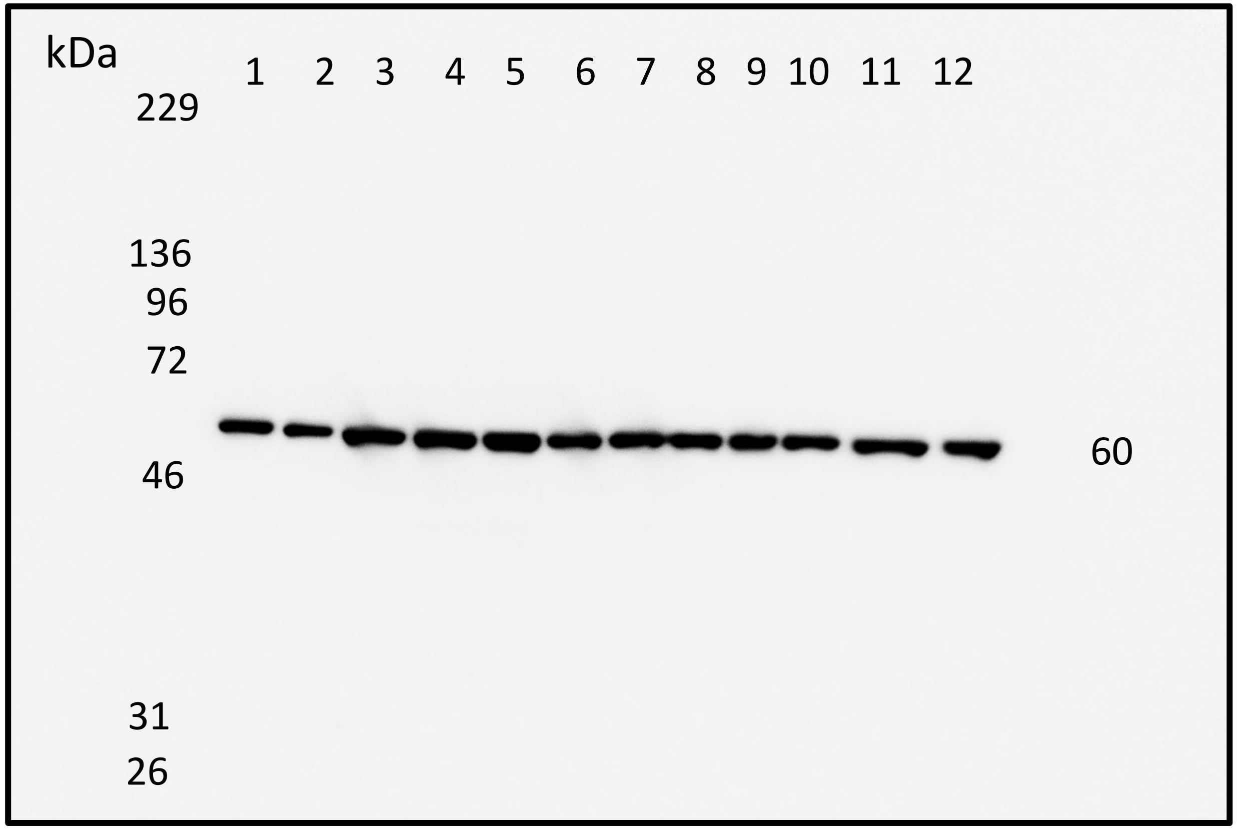

60kDa:100:66:166:175:150:110:119:104:87:104:113:121

LM8 treatment 24h

Treatment time 24 h

- 1. Everolimus 0 µM
- 2. Everolimus 5 µM
- 3. Everolimus 10 µM
- 4. Everolimus 0 µM Bortezomib 2.5nM
- 5. Everolimus 5 µM Bortezomib 2.5nM
- 6. Everolimus 10 µM Bortezomib 2.5nM

LM8 24 h treatment β actin

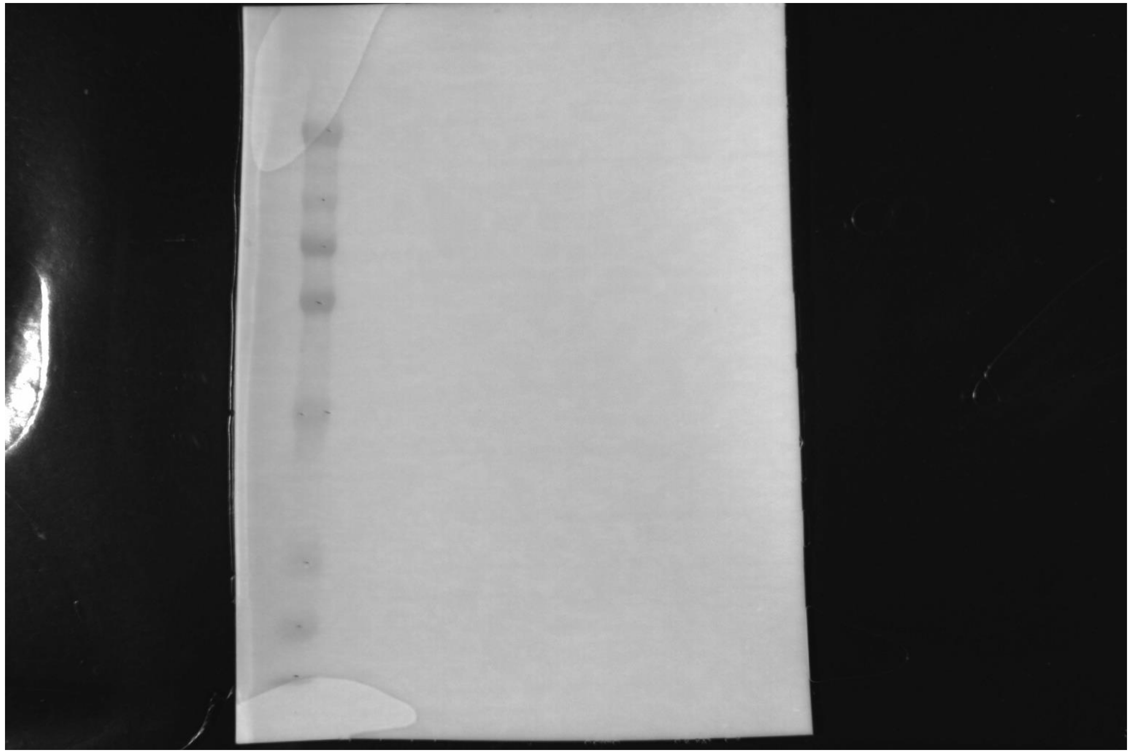

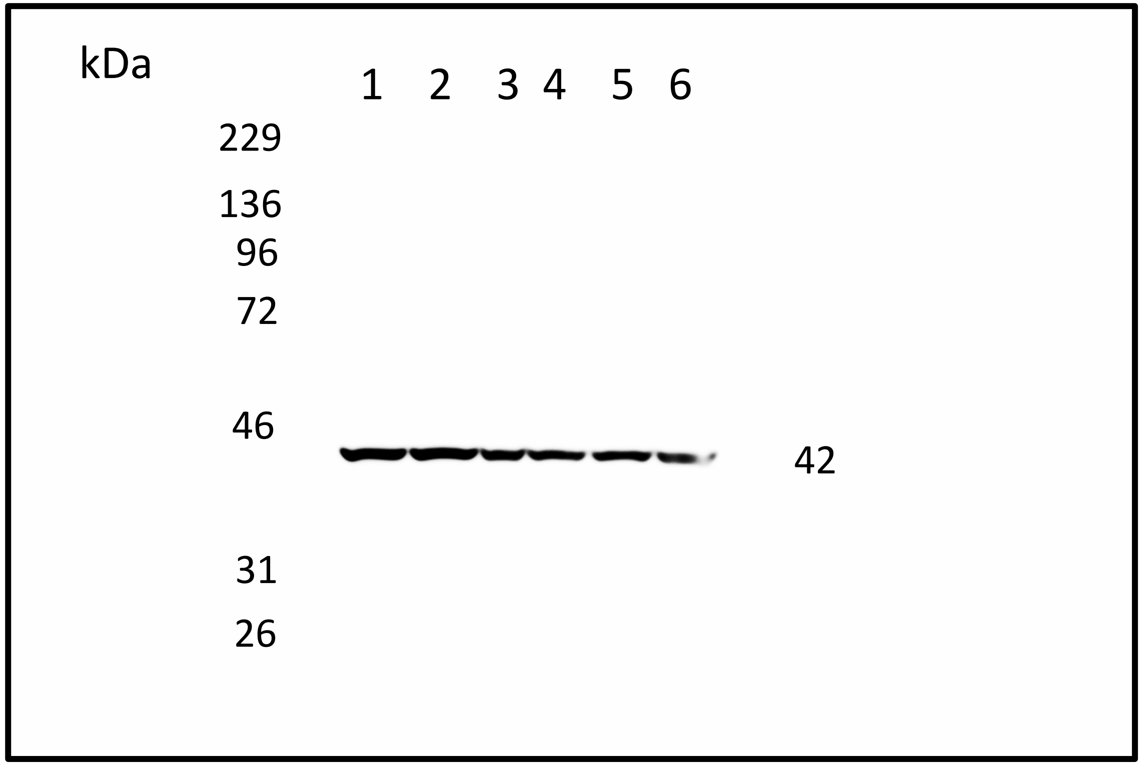

42kDa:100:102:43:40:63:21

LM8 24 h treatment cleaved caspase 3

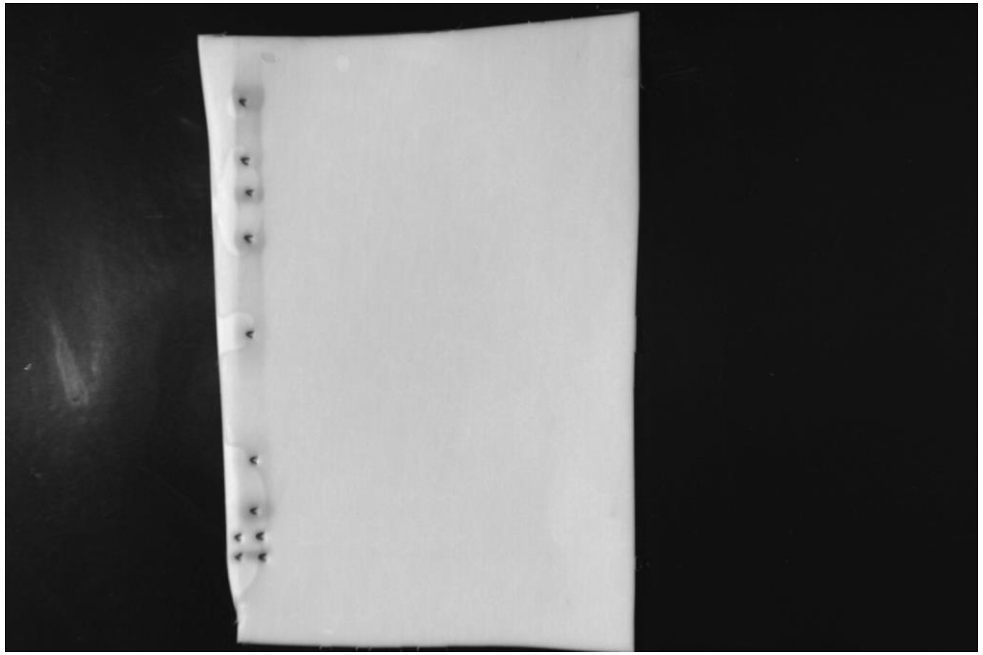

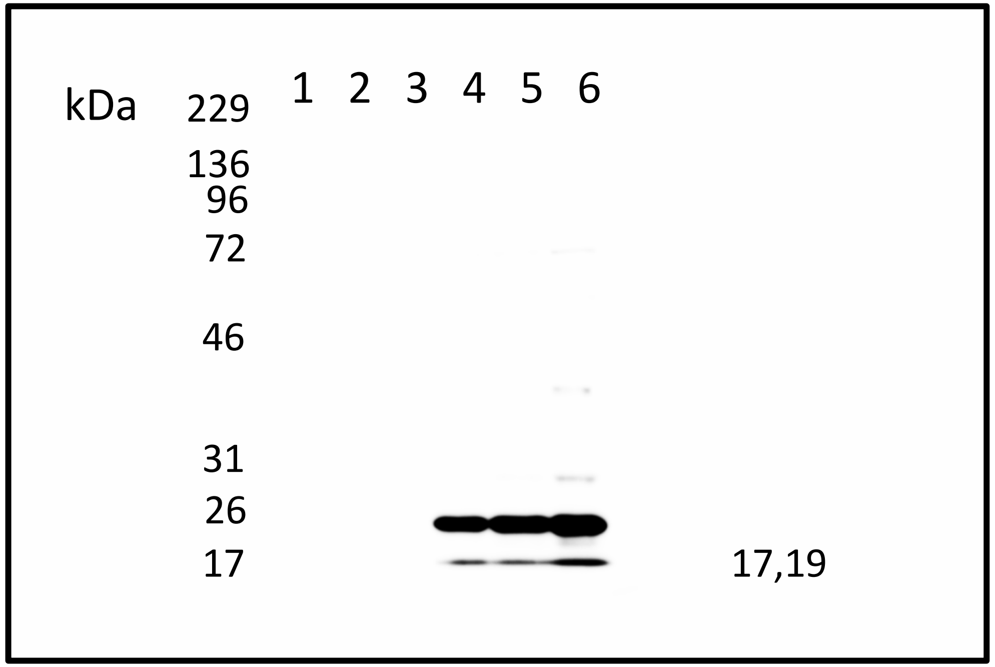

17,19Da:0:0:0:22:25:100

LM8 24h treatment cleaved PARP

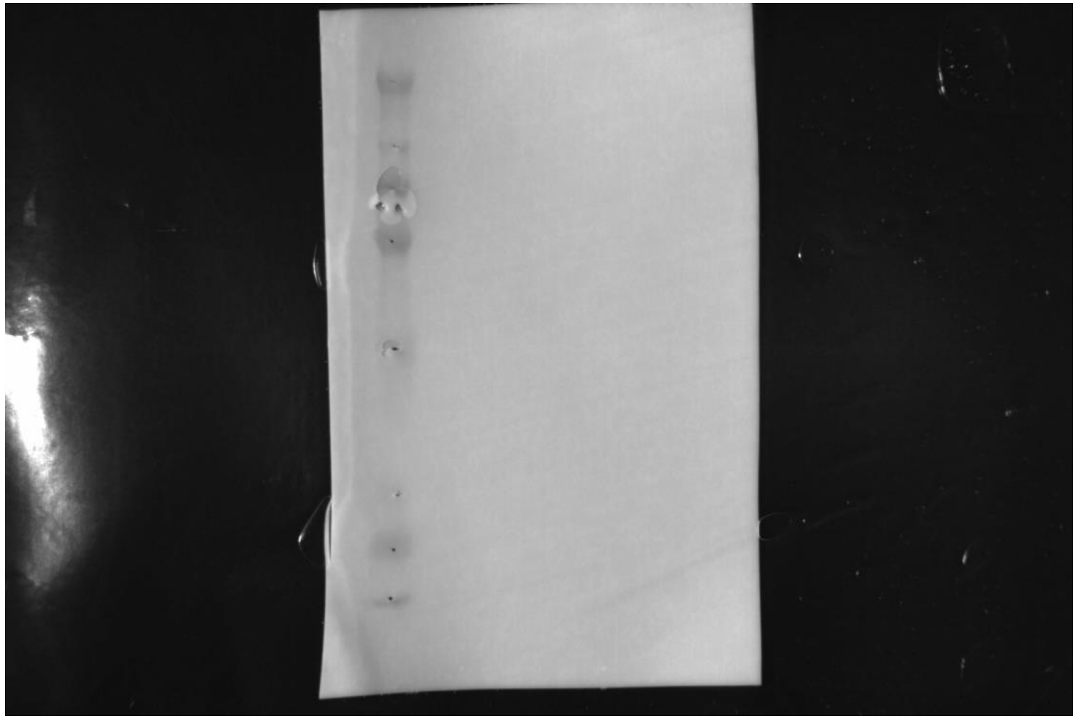

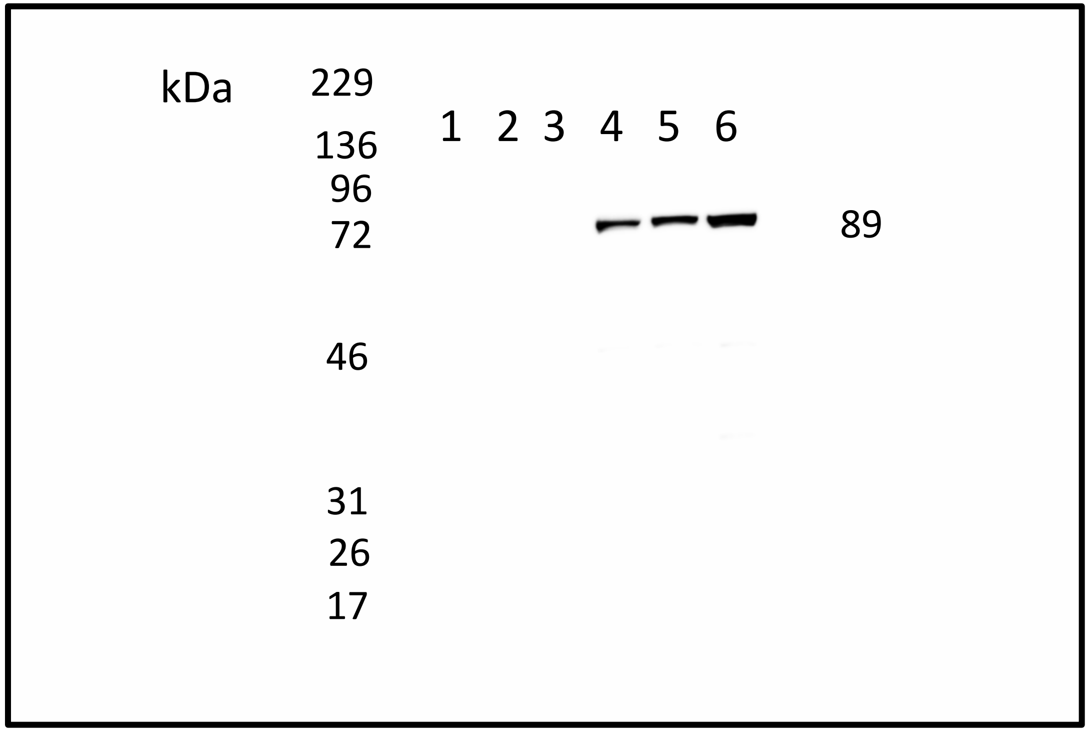

89Da:0:0:0:31:51:100

LM8 24h treatment p-ERK

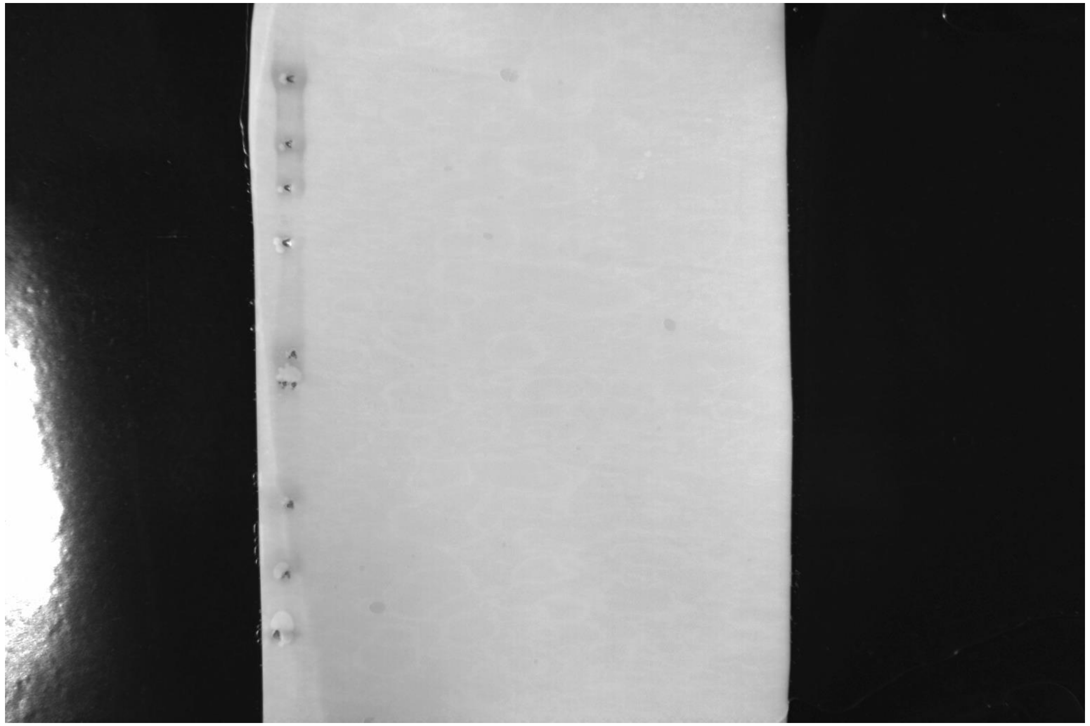

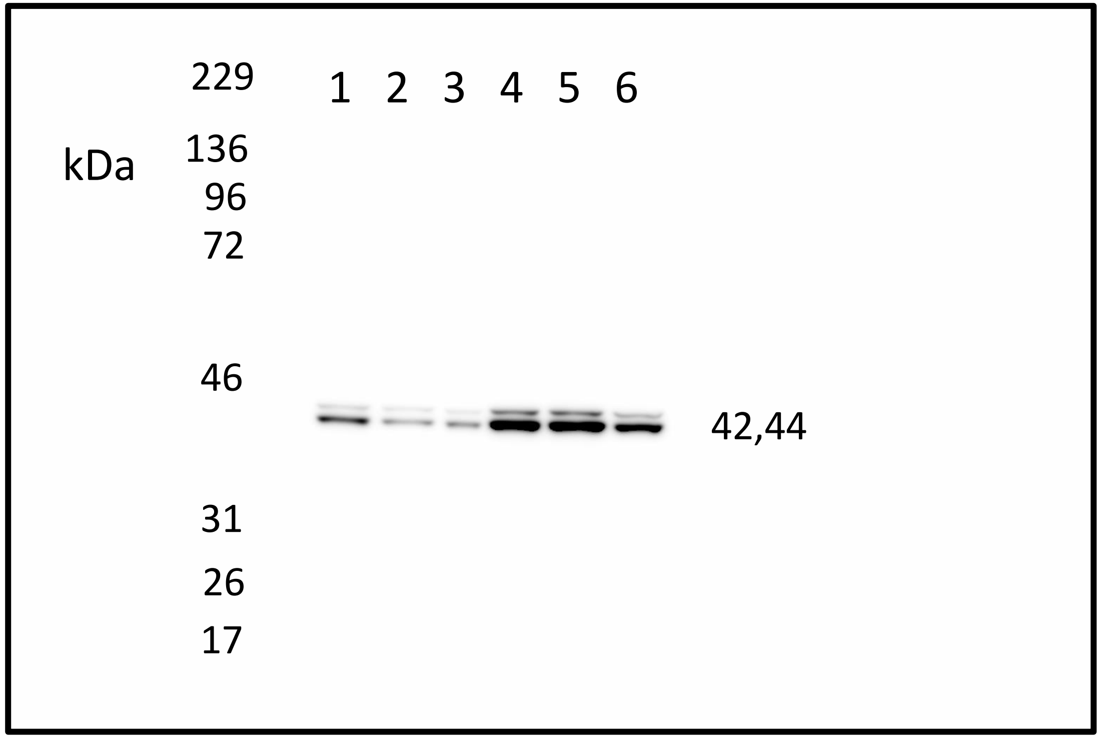

42,44Da:100:23:29:481:565:329

LM8 24h treatment ERK

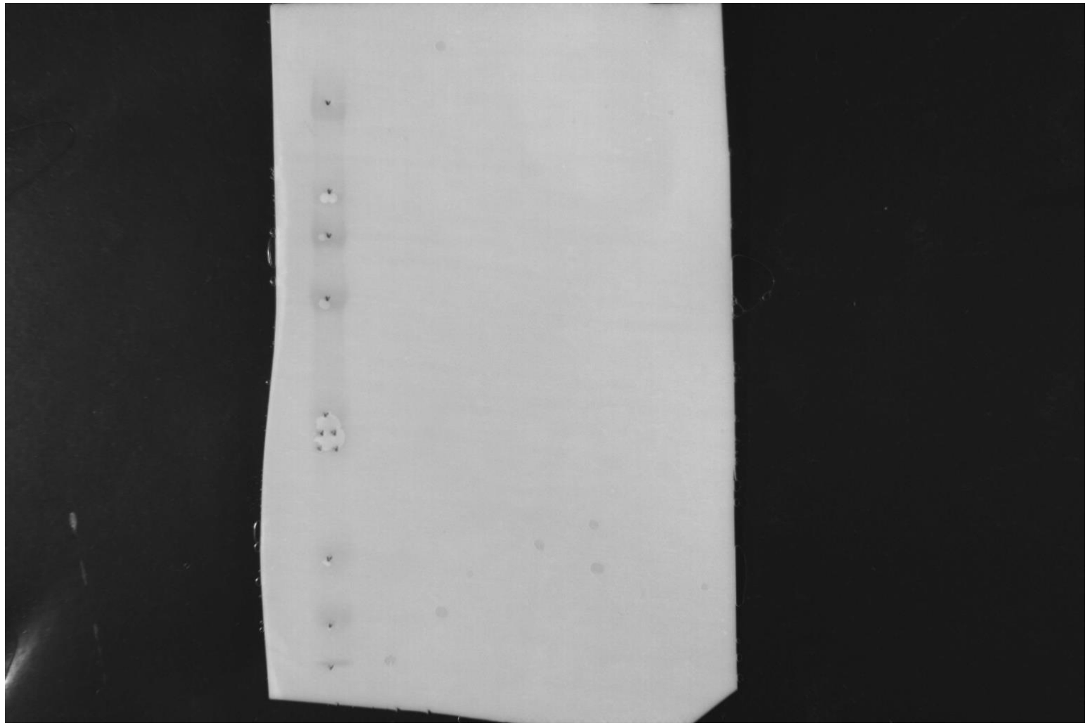

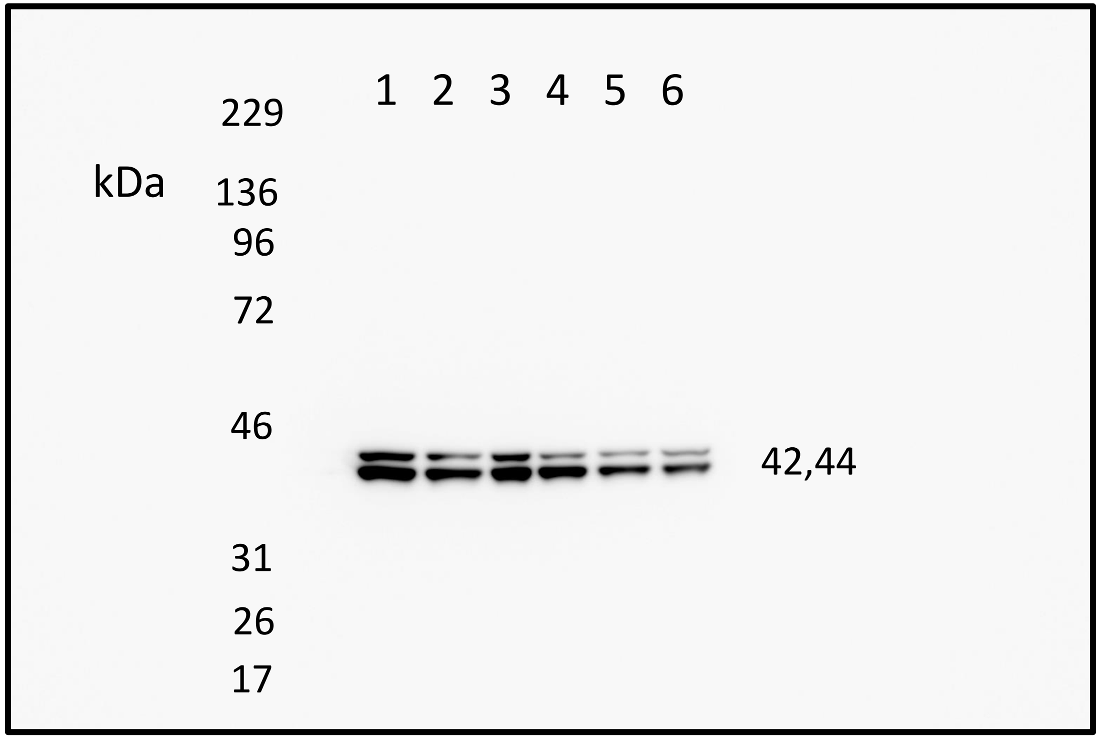

42,44Da:100:47:37:47:28:18

LM8 24 h treatment p-JNK

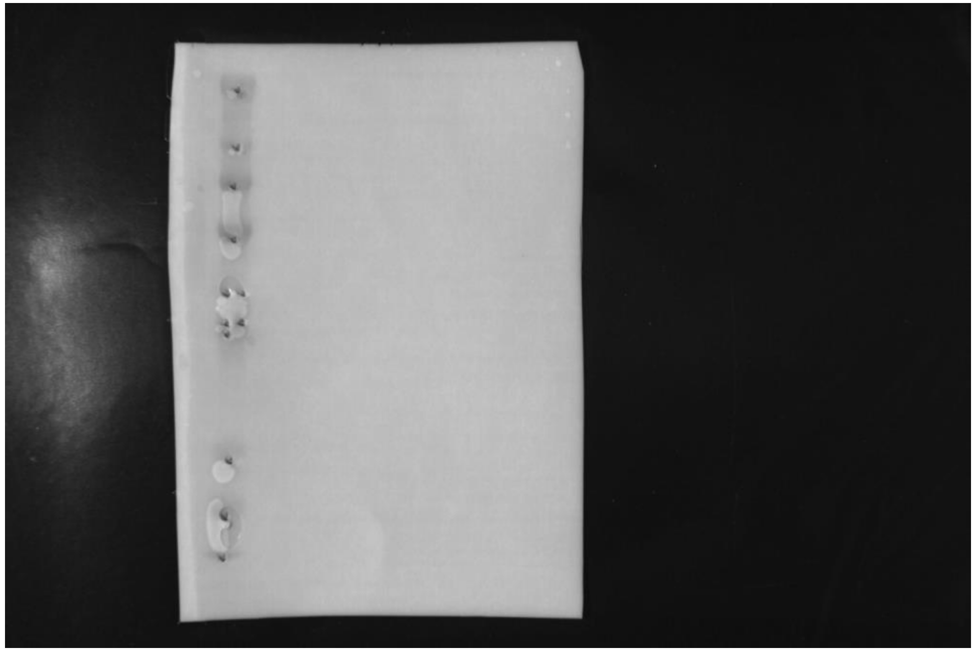

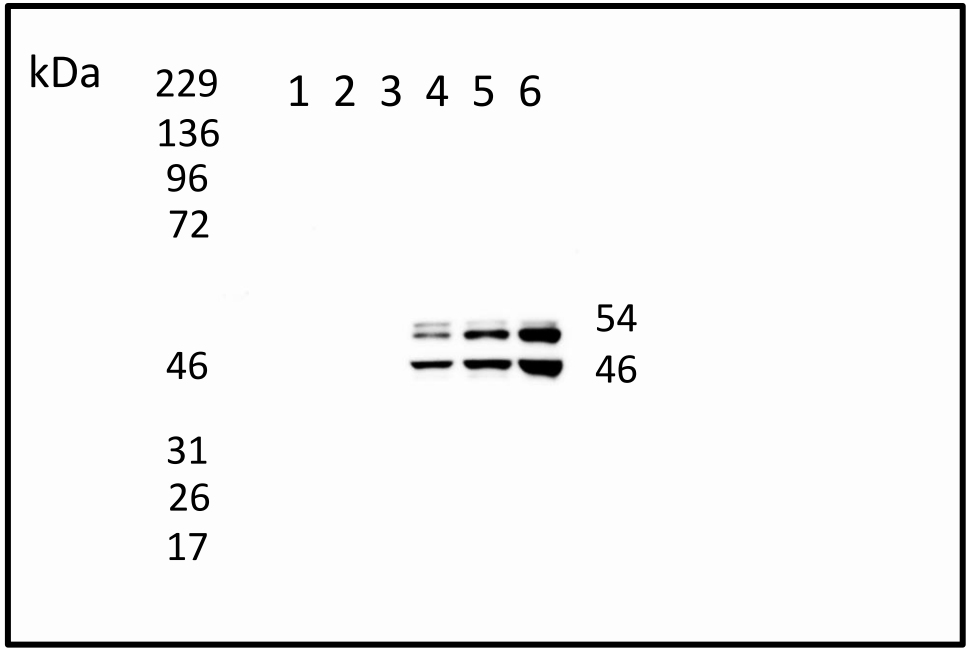

54kDa:0:0:0:18:51:100

46kDa:0:0:0:41:65:99

LM8 24 h treatment JNK

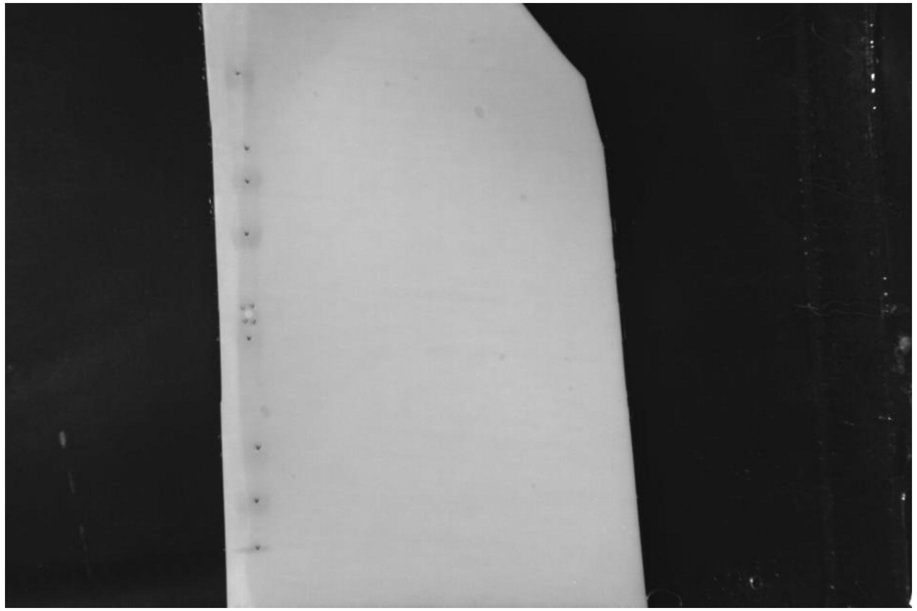

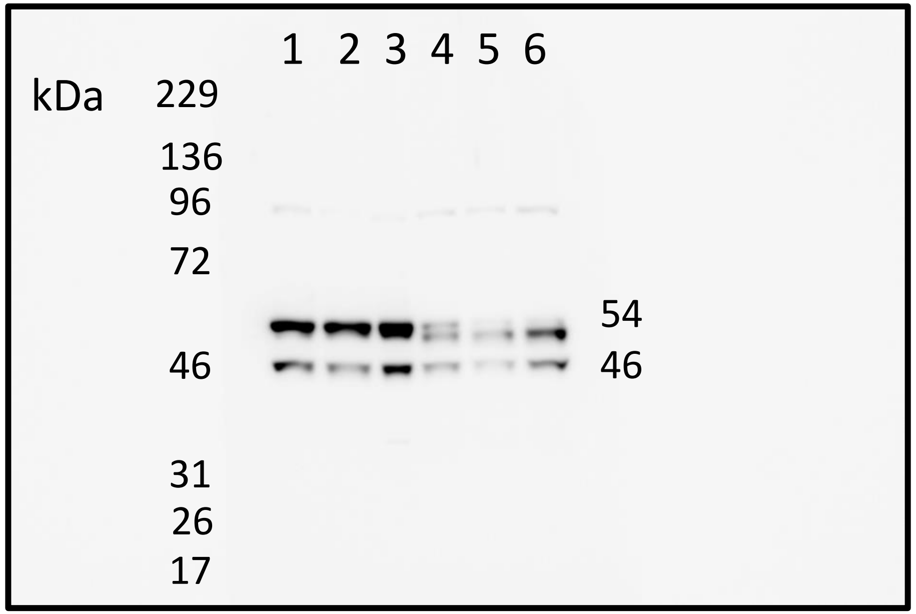

54kDa:225:273:220:46:25:100

46kDa:68:36:86:20:7:43

LM8 24 h treatment p-p38

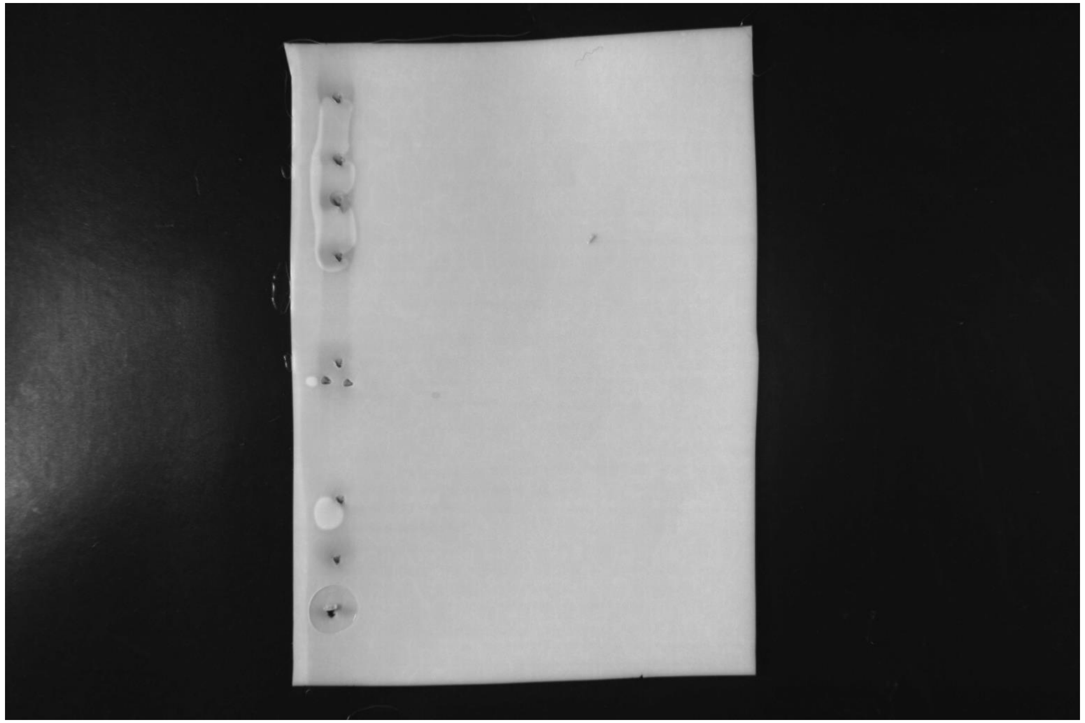

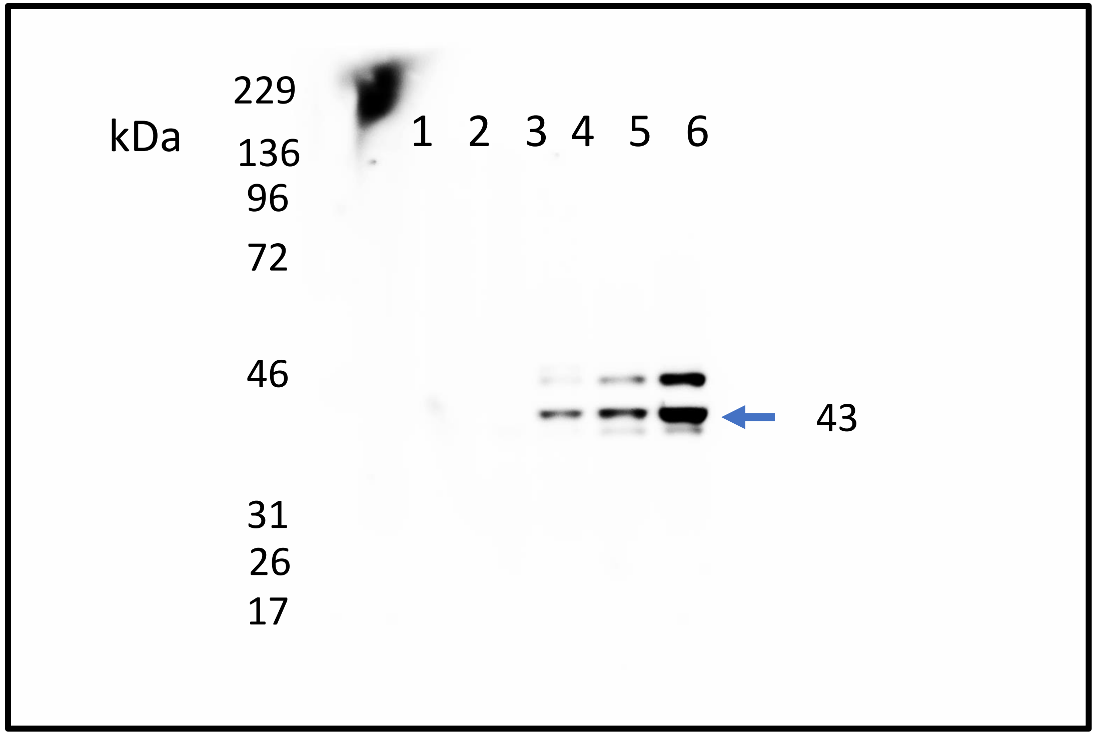

43kDa:0:0:0:9:24:100

LM8 24 h treatment p38

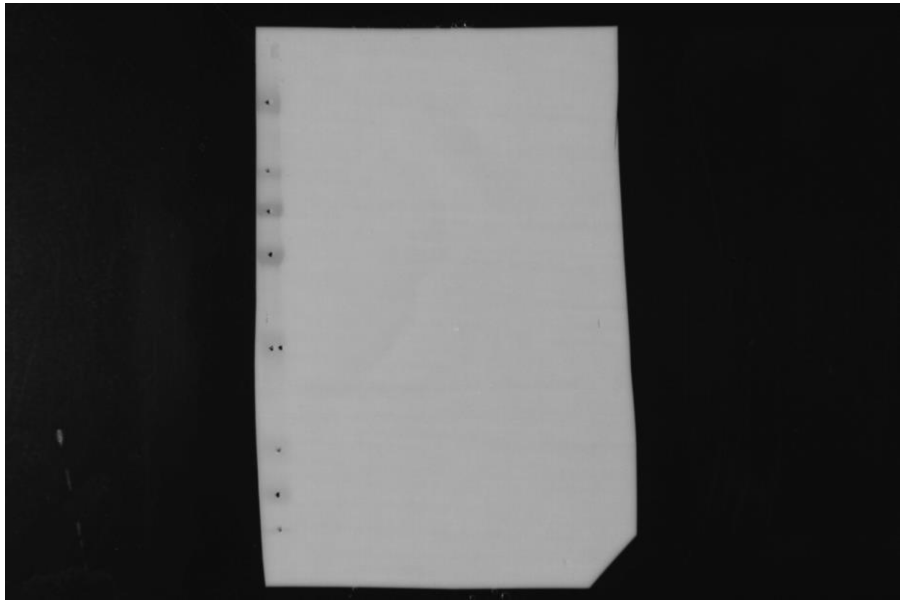

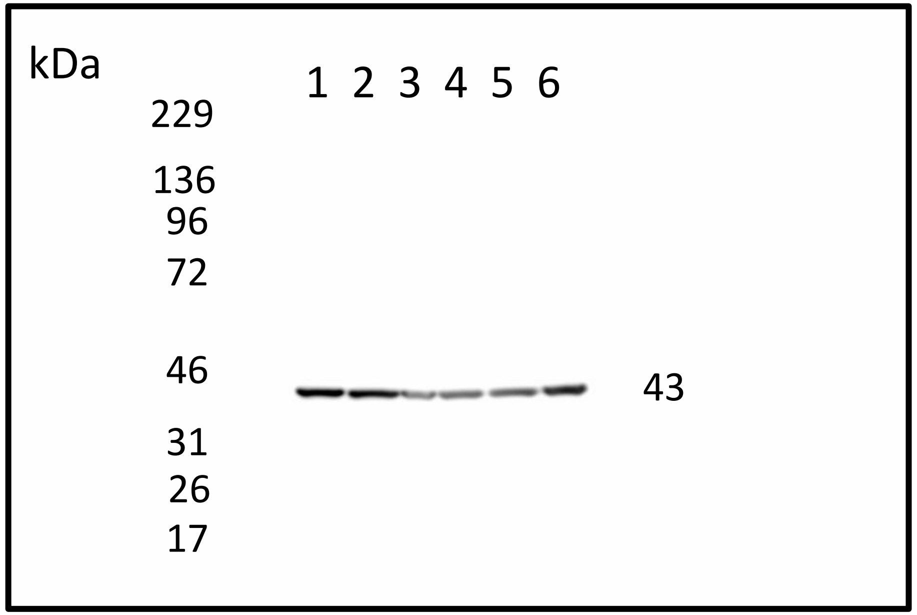

43kDa:203:117:34:42:49:100

LM8 24 h treatment MYC

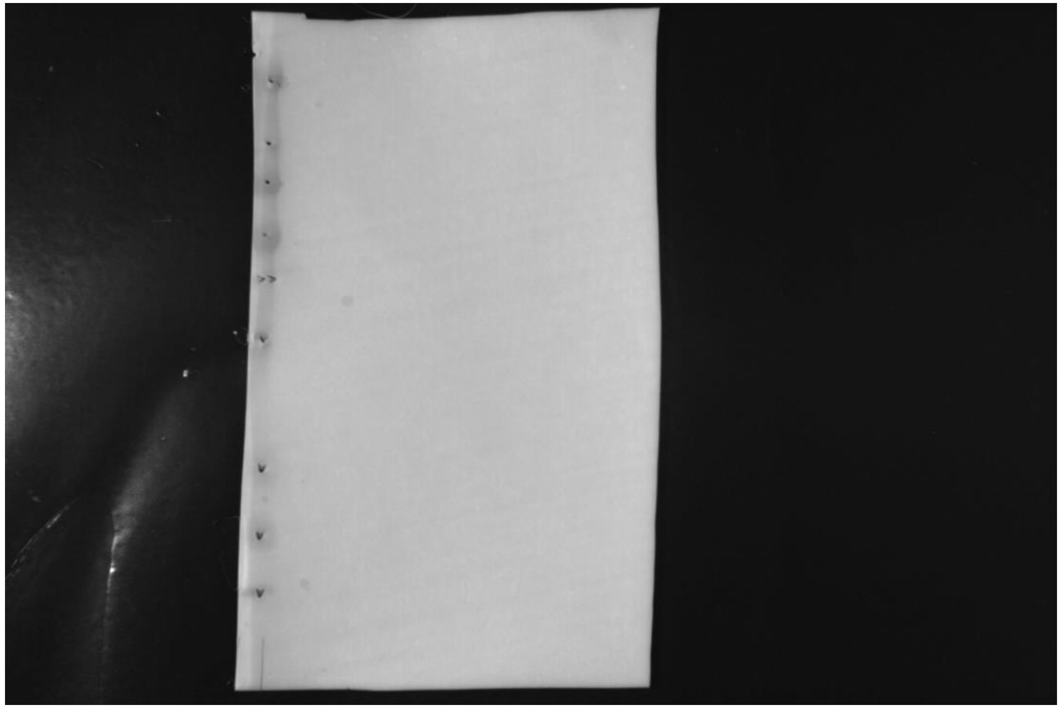

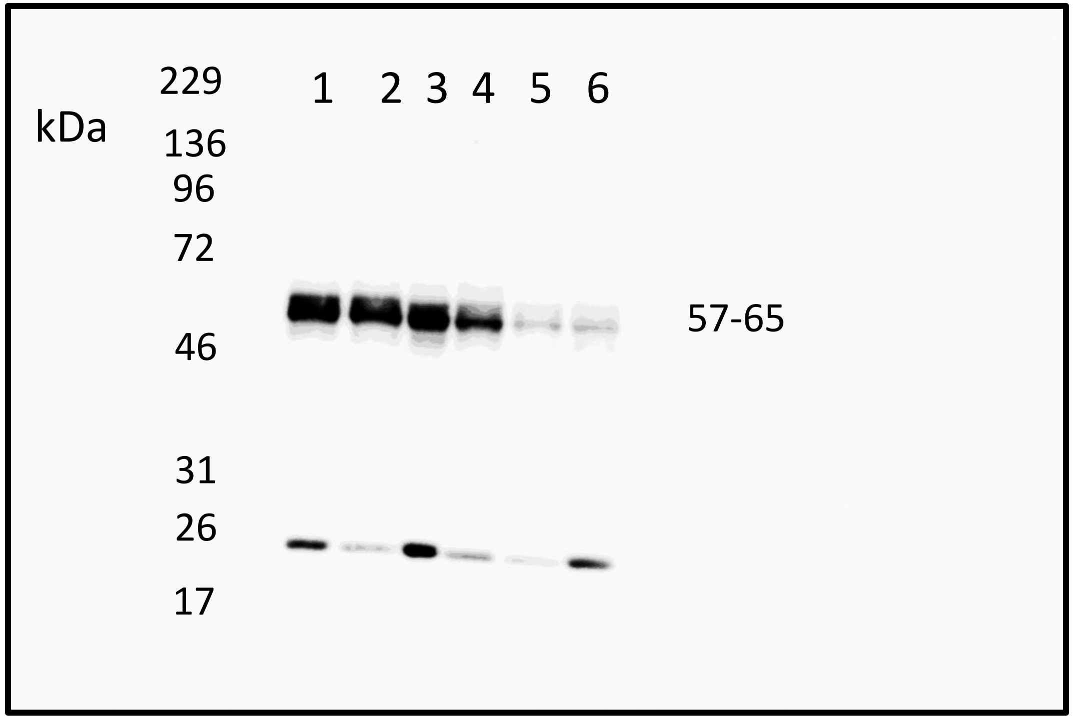

57-65kDa:100:87:74:46:4:4

LM8 24 h treatment Survivin

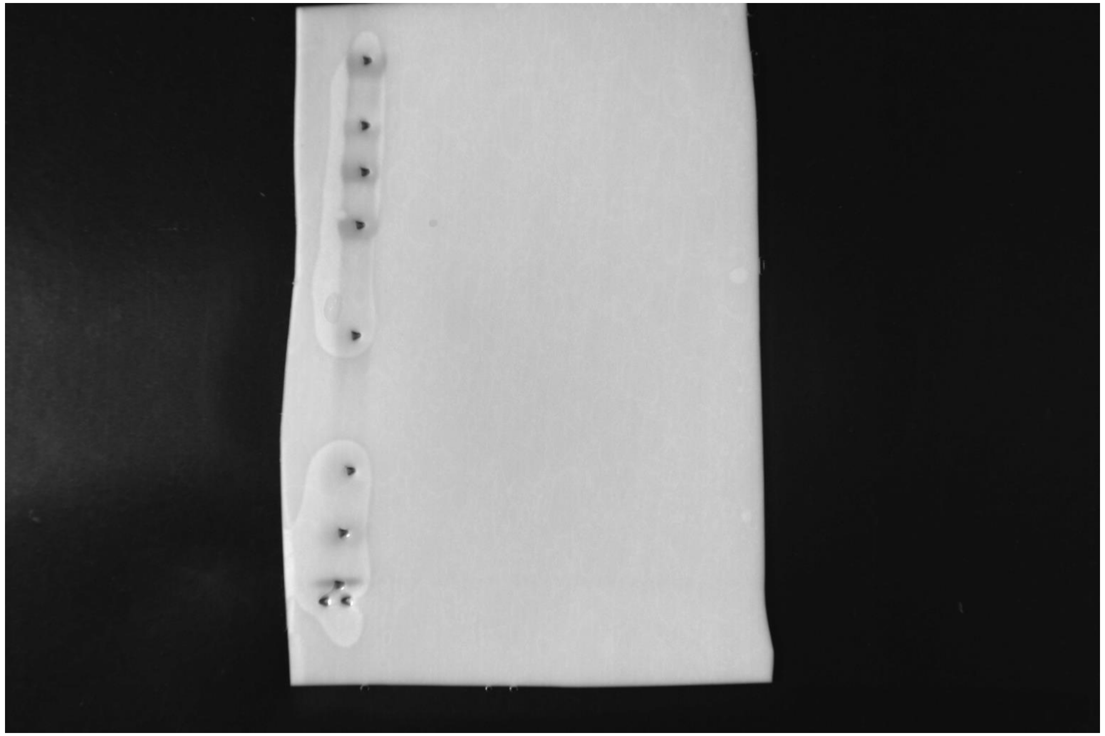

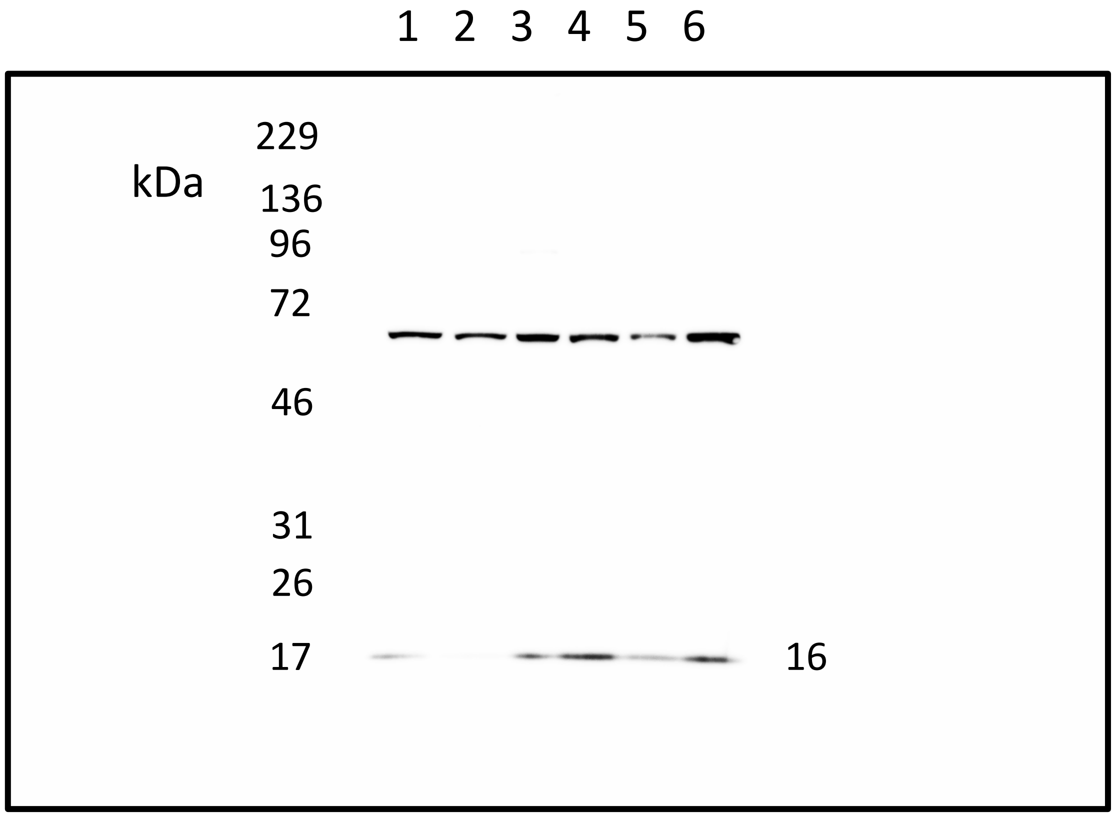

16kDa:17:1:41:127:27:100

LM8 24 h treatment p-AKT

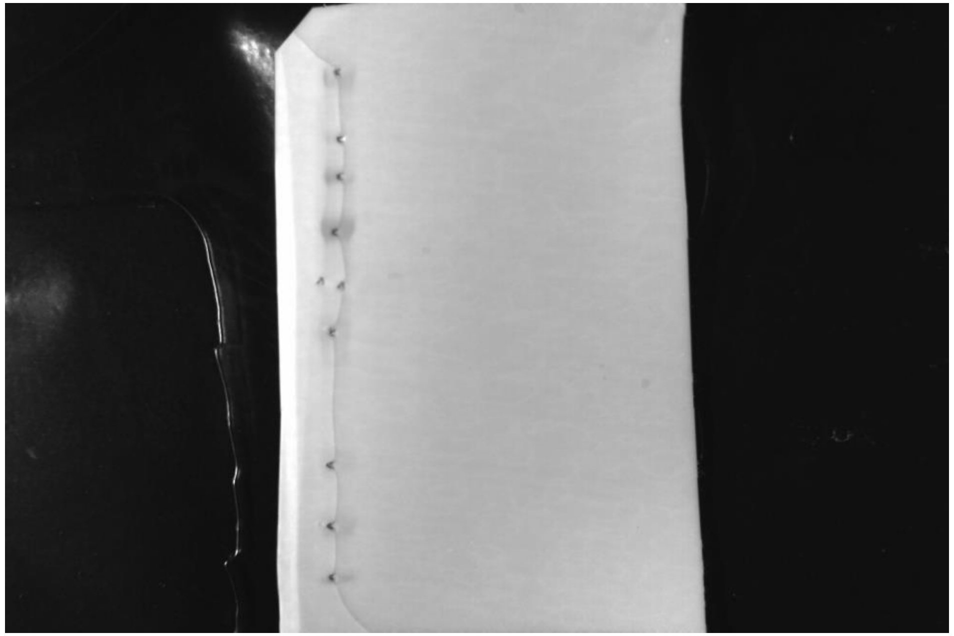

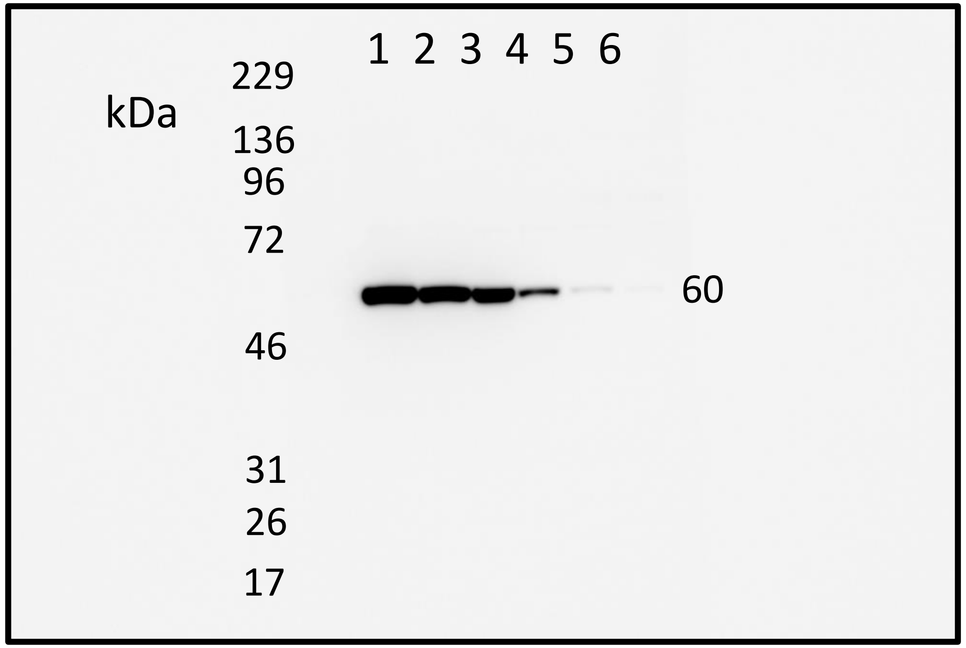

60kDa:100:78:59:13:0.3:0

LM8 24 h treatment AKT

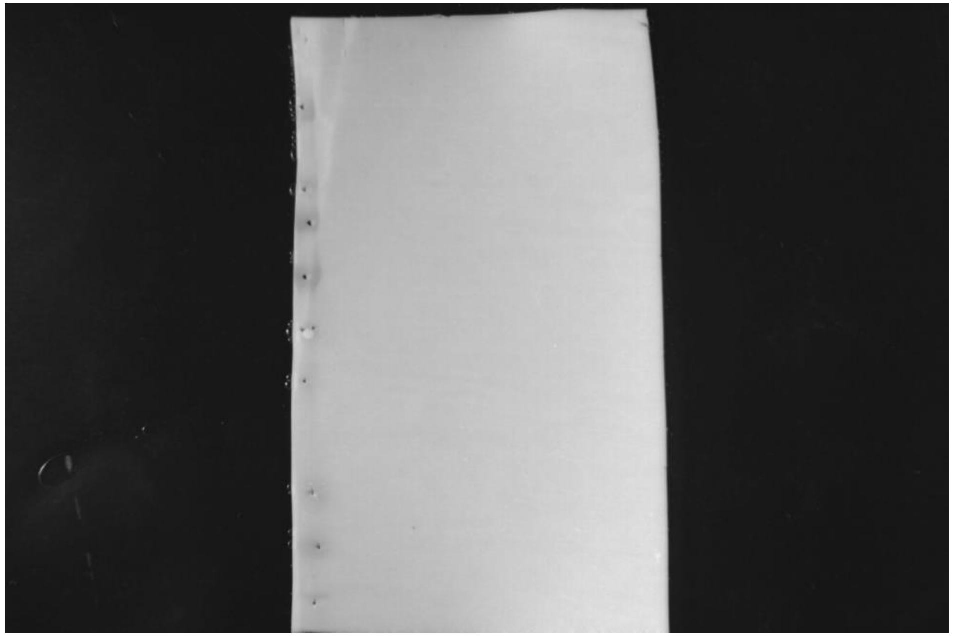

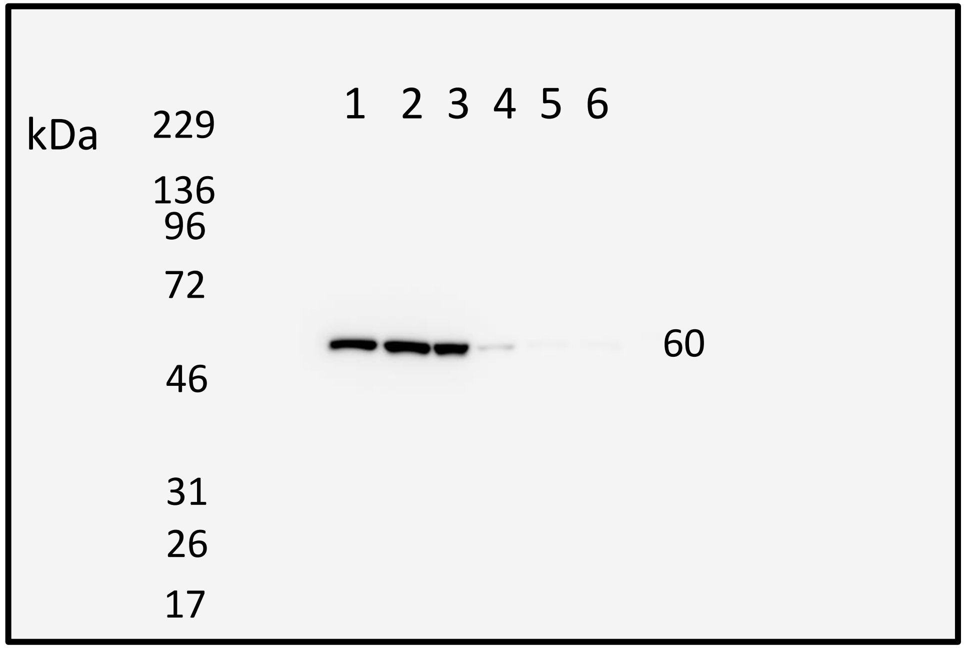

60kDa:100:112:53:2:0:0
